# Elucidating Local Structure and Positional Effect of Dopants in Colloidal Transition Metal Dichalcogenide Nanosheets for Catalytic Hydrogenolysis

**DOI:** 10.1021/acs.jpcc.3c07408

**Published:** 2024-03-06

**Authors:** Steven L. Farrell, Mersal Khwaja, Ingrid J. Paredes, Christopher Oyuela, William Clarke, Noah Osinski, Amani M. Ebrahim, Shlok J. Paul, Haripriya Kannan, Håvard Mo̷lnås, Lu Ma, Steven N. Ehrlich, Xiangyu Liu, Elisa Riedo, Srinivas Rangarajan, Anatoly I. Frenkel, Ayaskanta Sahu

**Affiliations:** † Department of Chemical and Biomolecular Engineering, New York University, Brooklyn, New York 11201, United States; ‡ Department of Materials Science and Chemical Engineering, Stony Brook University, Stony Brook, New York 11794, United States; § Department of Chemical and Biomolecular Engineering, Lehigh University, Bethlehem, Pennsylvania 18015, United States; ∥ National Synchrotron Light Source II, Brookhaven National Laboratory, Upton, New York 11973, United States; ⊥ Chemistry Division, Brookhaven National Laboratory, Upton, New York 11973, United States

## Abstract

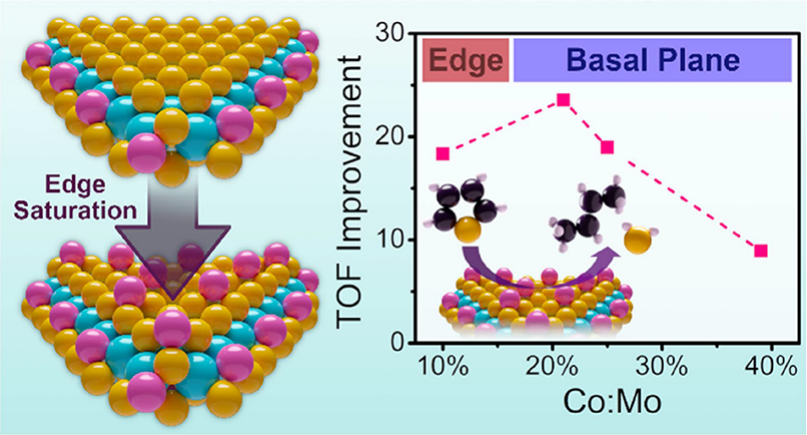

Tailoring nanoscale catalysts to targeted applications is a vital component in reducing the carbon footprint of industrial processes; however, understanding and controlling the nanostructure influence on catalysts is challenging. Molybdenum disulfide (MoS_2_), a transition metal dichalcogenide (TMD) material, is a popular example of a nonplatinum-group-metal catalyst with tunable nanoscale properties. Doping with transition metal atoms, such as cobalt, is one method of enhancing its catalytic properties. However, the location and influence of dopant atoms on catalyst behavior are poorly understood. To investigate this knowledge gap, we studied the influence of Co dopants in MoS_2_ nanosheets on catalytic hydrodesulfurization (HDS) through a well-controlled, ligand-directed, tunable colloidal doping approach. X-ray absorption spectroscopy and density functional theory calculations revealed the nonmonotonous relationship between dopant concentration, location, and activity in HDS. Catalyst activity peaked at 21% Co:Mo as Co saturates the edge sites and begins basal plane doping. While Co prefers to dope the edges over basal sites, basal Co atoms are demonstrably more catalytically active than edge Co. These findings provide insight into the hydrogenolysis behavior of doped TMDs and can be extended to other TMD materials.

## Introduction

Hydrogenolysis (or hydrotreating) is a catalytic process used on an industrial scale in applications such as , among other things, removing sulfur compounds from crude oil to reduce sulfur oxides (SOx) pollution and for valorizing biomass into biofuels by deoxygenation.^1,2^ To reduce the energy demand of hydrogenolysis, tailoring catalysts for specific applications, such as hydrodesulfurization (HDS) and hydrodenitrogenation (HDN) of fuel feedstocks^3^ and hydrodeoxygenation (HDO) of biomass^4^ is paramount. In this work, we focus on HDS, which is presently vital to preventing SOx pollution as the demand for a global shift to more renewable energy sources occurs. SOx emissions from petroleum refining largely contribute to smog formation, especially in densely populated industrial regions, and as such are heavily legislated.^1^ Furthermore, any remaining sulfur in the resulting fuel deactivates precious metal catalysts commonly used for tailpipe emissions control in vehicles. Removing sulfur contaminants prior to refining is regularly achieved through the use of catalytic hydrogenolysis at elevated temperatures and pressures to break down organosulfur compounds into hydrocarbons and hydrogen sulfide gas.^5^

MoS_2_ is of great interest to hydrogenolysis, and to the wider catalysis community, having long been used as a base for catalysts in petroleum refining.^6^ Compared with platinum group metal (PGM) catalysts traditionally used in hydrogenation reactions, MoS_2_ is a desirable substitute since it is precious metal-free and resistant to sulfur poisoning, which renders PGMs ineffective for desulfurization applications.^7^ MoS_2_-based catalysts are used in a variety of applications, including HDS and more recently in electrochemical reactions such as hydrogen evolution.^8−10^ As a member of the class of compounds known as transition metal dichalcogenides (TMD), which take the form MX_2_ (M = transition metal, X = S, Se, or Te), MoS_2_ exhibits covalent bonding in the *x*- and *y*-directions but is bound only by van der Waals forces in the *z*-direction. This crystal structure allows MoS_2_ to stably form single- or few-layer 2D nanosheets with high surface area by a variety of straightforward synthetic methods, which could potentially provide ample active sites for catalysis.^11^ The catalytic activity of MoS_2_, however, is derived primarily from the undercoordinated edge sites and the presence of sulfur vacancies. The morphology and edge structure of MoS_2_, especially at the nanoscale, can be modulated by growth conditions or modified for tunable activity and selectivity in hydrogenolysis reactions.^12−14^ However, the remaining atoms in the basal plane are catalytically inert.^15,16^ A common method for improving activity is to dope MoS_2_ with transition metals, potentially activating the basal plane in addition to the edge sites and, therefore, maximizing the catalytic surface area. Single-atom transition metal dopants, particularly Co and to some extent Ni, have been used to decorate the surface and improve activity among other methods.^17,18^ Previous studies on the synthesis of Co-doped MoS_2_ have proposed or observed a variety of dopant locations, including single metal atoms adsorbing along the edges,^19^ intercalated between layers,^20^ atop the basal plane,^21,22^ or even substituting S or Mo atoms.^23,24^ From these studies, it became apparent that the synthetic approach influences the geometric locations of the dopants. Dopant atoms may thus appear at a specific site or a combination of sites, preventing a clear and direct understanding of the structure–activity relationship in this class of materials. This crucial understanding of dopant location and its impact on subsequent activity will help optimize catalytic processes by targeted synthesis of doped MoS_2_ with specific dopant locations.

Other TMDs, such as NbS_2_ and WS_2_, combined with an array of metal dopants (e.g., Fe, Ni, Cu), have attracted interest for a variety of key reactions and applications,^25−29^ ranging from catalysis to energy storage and optoelectronics. With the growing combinations of TMD and dopant atoms, tunability in dopant concentrations, and heterogeneity among dopant locations, it has become challenging to optimally design TMD catalysts for new and existing applications. To approach this challenge, this work intends to study how dopant location and local structure impact catalytic activity, particularly in systems where a variety of dopant structures coexist, to further elucidate the structure–activity relationship for catalyst design. Furthermore, direct observation of the location of single-atom dopants has also proven challenging, requiring advanced characterization techniques to determine the local structure and distribution across the nanosheet.^28−30^ By determining the atom location accurately and subsequently designing synthetic approaches to control both concentration and location, we can derive a correlation between doping mechanisms and the structure–activity relationship of dopant-TMD systems for optimal catalyst design. In this study, we investigate the doping of MoS_2_ with few atoms of Co for a model system of HDS (thiophene) to elucidate structure–property–activity relationships.

Characterization of nanometer-scale catalysts presents a formidable challenge due to the ensemble-averaging nature of most suitable characterization techniques, such as X-ray diffraction (XRD), energy dispersive X-ray spectroscopy (EDX), and in particular X-ray absorption spectroscopy (XAS).^29,31−35^ In order to accurately study individual Co locations based on XAS, size control is crucial and the MoS_2_ particle size and Co placement distributions must be narrow, which can be afforded by hot-injection colloidal syntheses.^36^ These bottom-up synthetic approaches allow for precise size and shape control of host MoS_2_ sheets, with the only difference between doped and undoped samples being the number of Co dopant atoms. This systematic control of particle morphology allows for the generation of a set of catalysts that are easily comparable across the parameter space.

In the present work, we investigate the activity of colloidally prepared Co-doped MoS_2_ nanosheets for HDS of thiophene, specifically probing the effects of Co loading (concentration) and local structure on the catalytic activity. The MoS_2_ nanosheets are prepared using hot-injection colloidal synthesis, which affords simple preparation of catalysts with a narrow, reproducible size distribution;^37^ doping occurs by a swift injection of the desired amount of Co precursor during synthesis. We demonstrate a correlation between the dopant concentration and the sites where the dopant atoms affix to the surface, primarily employing synchrotron techniques to observe the local structure of finely dispersed Co atoms. This approach couples extensive analysis with XAS at the Co, Mo, and S K-edges with density functional theory (DFT) calculations to develop a complete picture of the Co environment. A multimodal combination of XRD, XAS, and XPS is employed to confirm the finely dispersed state of Co atoms and the lack of Co metal clustering. We then tie this trend to their catalytic activity in HDS, using a batch reactor containing thiophene as a model desulfurization analogue. Previous work has demonstrated the potential of colloidally synthesized Co-MoS_2_ nanosheets as catalysts for HDS,^38^ but no studies have been performed to elucidate the relationship between Co location and its catalytic properties in colloidal MoS_2_ nanosheets, which motivates this work.

Although the focus of this work is on HDS as a model reaction, investigating the dopant local structure can be utilized to predict and design tailored 2D catalysts for other forms of hydrotreatment such as HDO of biomass-derived lignin for sustainable biofuels or the prevention of acid rain by HDN of amine-based organic compounds. We can potentially extend the analytical methods herein to other dopant-TMD and reaction combinations, considering the wide variety of reactions demonstrated to be catalyzed by doped TMDs. By understanding the precise geometry and location in single-atom doped TMDs, and the influence of the dopant position on catalytic activity, we can better design next-generation catalysts for optimal activity.

## Experimental Methods

### Chemicals

Molybdenum(V) chloride (95%), cobalt (ii) chloride (97%), *bis*(trimethylsilyl)sulfide (a.k.a., hexamethyldisilathiane, synthesis grade), oleylamine (70%, technical grade), oleic acid (90%, technical grade), 1-ODE (90%, technical grade), cyclohexane (≥99%, ACS reagent grade), hexane (95%, anhydrous), methanol (99.8% anhydrous), thiophene (≥99%), and *n*-decane (≥99%, synthesis grade) were purchased from Sigma-Aldrich. Acetone (99.8%, extra dry) and 1,2,3,4-tetrahydronaphthalene (a.k.a. tetralin, TCI America, ≥ 97%) were purchased from VWR. All chemicals were used without further purification with the exception of oleylamine and 1-ODE, which were each separately degassed for 1 h by cycling between nitrogen flow and vacuum on a Schlenk line at 80 °C prior to use.

### Colloidal Synthesis of Co-MoS_2_ Nanosheets

Co-MoS_2_ nanosheets were prepared via a solvothermal hot-injection method. In a typical synthesis, 2.0 mmol of MoCl_5_ and 10 mL of previously degassed oleylamine were added to a 50 mL three-neck flask inside a nitrogen-filled glovebox. The flask was attached to a water-filled condenser and Schlenk line and heated to 130 °C and then degassed for 1 h by cycling between nitrogen flow and vacuum. A separate, 25 mL three-neck flask containing 5 mL oleic acid and 0.2, 0.4, 0.6, or 1.0 mmol of CoCl_2_ (depending on the dopant loading to be studied) was also degassed at 130 °C. In the glovebox, 4 mmol of *bis*(trimethylsilyl)sulfide (TMS_2_S, 0.84 mL) was added to 2 mL of previously degassed 1-octadecene (ODE). Under a nitrogen flow, the flask containing the MoCl_5_/oleylamine mixture was raised to 180 °C, and then the TMS_2_S/ODE mixture was swiftly injected into the flask. After 3 min to allow nucleation of MoS_2_ nanosheets, the contents of the CoCl_2_/oleic mixture flask were removed by a glass syringe and swiftly injected into the MoCl_5_/oleylamine flask. The delay between injections is meant to allow the MoS_2_ sheets to nucleate and begin growth first, thus, depleting the S-content in the reaction mixture and preventing the formation of cobalt sulfide. The entire reaction mixture was stirred under nitrogen flow at 180 °C for 30 min, then allowed to cool naturally to room temperature.

After it cooled, the flask was sealed and brought into a nitrogen-filled glovebox. The contents were split among three 50 mL centrifuge tubes. An excess of acetone and methanol were added to each tube. The tubes were briefly sonicated to disperse the mixture and then centrifuged at 9500 rotations per minute (RPM) for 10 min, and the supernatant was discarded afterward. This process was repeated twice more with an additional 5 mL of hexane added to the pellet to disperse the particles before drying under a vacuum. To synthesize Co-free pure MoS_2_ nanosheets, the above procedure is followed, but no CoCl_2_ is added to the oleic acid.

### HDS of Thiophene

The benchmark HDS activity of the synthesized nanosheets was measured in a 316 stainless steel stirred autoclave benchtop reactor from Parr Instruments (Model 4564, 160 mL chamber volume). The reaction mixture was created by dissolving 500 mg of thiophene in 50 mL of 1,2,3,4-tetrahydronaphthalene (tetralin). 500 mg of *n*-decane was also added to the solution as an inert reference. 0.5 mL of solution was removed and analyzed via gas chromatography coupled with mass spectrometry using a Shimadzu QP2010 GC–MS. The sample was analyzed three times to determine the average concentration of thiophene.

The catalyst (20 mg) was then added to the remaining reaction mixture and sonicated for 10 min to fully disperse. The contents were transferred to the reactor and the chamber was purged by filling with 95%N_2_/5%H_2_ gas up to 13.8 bar-g, then vented and repeated twice more. The chamber was then pressurized with 95%N_2_/5%H_2_ gas to 10.3 bar-g and heated to 300 °C with a mixing speed of 320 rpm. The reaction was held at temperature for up to 3 h, then allowed to cool naturally to room temperature and subsequently depressurized. To analyze the remaining thiophene content, 0.5 mL of solution was extracted and filtered to remove the catalyst. The filtrate was then analyzed by gas chromatography–mass spectrometry (GC–MS) following the same procedure as the prereaction mixture sample.

## Computational Methods

All calculations (unless specified otherwise) were carried out for a single-layer freestanding (i.e., unsupported) hexagonal nanoparticle model of MoS_2_ with Mo-edge containing six Mo atoms (including the corner atoms) while the S-edge contains three Mo atoms (including the corner atoms), reflecting the typical truncated triangular shape of a single-layer particle observed in STM experiments.^16^ Two layers of S atoms sandwich the Mo layer, such that they are in the trigonal prismatic positions characteristic of the 2H phase of MoS_2_. All figures in this article with MoS_2_ structures and adsorption configurations show Mo atoms in blue, S atoms in yellow, and Co atoms in pink. For ligands, C atoms are shown in black, O atoms in red, and H atoms in white. We, therefore, suggest that the given model captures the local electronic structures adequately to provide a comparative analysis between different locations along the periphery of the MoS_2_ nanoparticles used in experiments. We further assume that the sulfur edge is 100% S-decorated while the metal edge is 50% S-decorated, consistent with ab initio phase diagrams and STM observations.^39^ A single Co atom was included in the calculations in many cases (in different locations) to represent cobalt decoration of the MoS_2_ basal plane.

The calculations were carried out with VASP,^40,41^ a plane wave periodic DFT code. Generalized gradient approximation and projected augmented wave (PAW) potentials were used with PBE exchange-correlation functional and the D3 Grimme dispersion correction.^42−44^ All calculations were carried out in a box that had at least 10 Å of vacuum between the two images in all directions. Spin polarization is included in all calculations involving the MoS_2_ nanosheet. Plane wave and density wave cutoffs of 400 and 645 eV were used, respectively. A Gaussian smearing of 0.05 eV was used, and the energies were extrapolated to 0 K. Only gamma-point sampling was used, in view of the large dimensions of the supercell. The convergence criterion for geometric relaxation was set to 0.02 eV/Å. The energies computed using VASP are not reported as such; only energies relative to a reference are presented. The binding energy of hydrogen sulfide (H_2_S), BE_H_2_S_, was computed using the following equation:
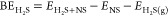
1where  is the energy of H_2_S adsorbed onto the nanosheet, *E*_NS_ is the energy of the free nanosheet, and *E*_H_2_S(g)_ is the energy of H_2_S in the gas phase.

## Results and Discussion

### Catalytic Activity in HDS

**Figure 1:**
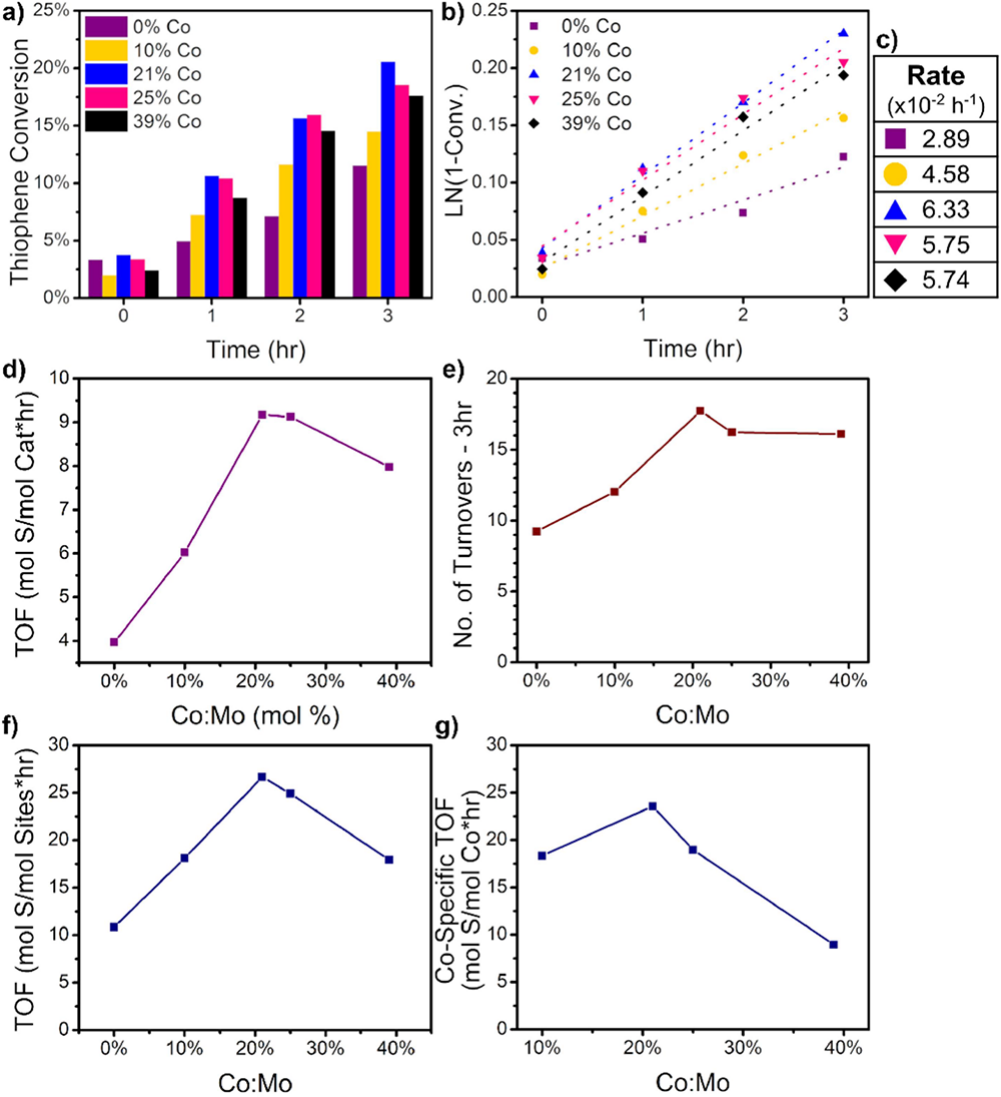
Hydrodesulfurization studies of Co-MoS_2_ catalysts: (a) thiophene conversion over time for various Co-doped MoS_2_ nanosheets, (b) first-order reaction rate kinetics of Co-MoS_2_ nanosheets (dotted lines represent the line of best fit), (c) table of first-order rate constants calculated from 1b, (d) turnover frequency—total mole basis, (e) total turnovers after 3 h—mole basis, (f) turnover frequency—mole sites basis, and (g) improvement rate in TOF—mole Co basis. Solid lines in (d–f) provide a visual guide showing the nonmonotonous trends in sample activity by varying Co concentration. 21% Co:Mo samples exhibited the highest performance in conversion of thiophene, indicating an optimal concentration of Co.

Samples of Co-MoS_2_ were prepared with a range of Co loadings with Co:Mo atomic ratios between 0 and 39% as determined by inductively coupled plasma mass spectroscopy (ICP-MS). HDS of thiophene was carried out in a batch reactor using a mixture of thiophene, *n*-decane (as a reference), and tetralin solvent. The tetralin solvent dispersed the organic-ligand-capped nanosheets and acted as a hydrogen donor, as noted by the presence of naphthalene after HDS (see reaction scheme, Figure S7 in SI). Colloidal synthesized MoS_2_ nanosheets (without Co) showed an increase in activity as compared to bulk MoS_2_, from 6.5 to 11.5% thiophene converted after 3 h (see Figure S8 in Supporting Information, SI). This increase can be attributed to the increase in the active edge surface area stemming from the reduced sheet size in the nanosheets. Figure 1a demonstrates the catalytic performance of Co-MoS_2_ nanosheets with various Co concentrations as a function of the time of reaction. While all Co-MoS_2_ samples show increased activity compared to pure MoS_2_ nanosheets, there is a notable nonmonotonous trend with the highest activity observed for 21% Co-MoS_2_.

Co makes an effective promoter of MoS_2_, increasing the rate of thiophene desulfurization over the cobalt-free catalyst.^6,7,45,46^ The role of Co:Mo ratio has long been investigated in studying HDS catalysts, with some literature reporting an optimum closer to 37–40% Co:Mo.^45,46^ According to Figure 1, there exists an optimal dopant concentration for our system; activity maximized in the range of 21–25% Co:Mo, but decreased as further Co was added (39%). 21% Co:Mo performed the best initially and after three h in HDS, converting 20.6% (103 mg) of thiophene. The data fit to a first-order reaction rate eq (Figure 1b), produced a linear plot whose slope is the reaction rate due to the pseudo-first-order nature of the reaction ascribed to the excess hydrogen. The rate constants of the reaction are listed in Figure 1c. Again, the 21% Co:Mo sample exhibited the highest reaction rate; 25% Co:Mo displayed similar activity, but 39% Co:Mo showed a suppressed reaction rate. Indeed, this is further evident in the turnover frequency (TOF) measured per mole of the catalyst shown in Figure 1d and the number of turnovers in Figure 1e. We have also computed the TOF per unit mole of sites in Figure 1f; this is done by estimating the number of potential active sites, including MoS_2_ edge sites and Co atomic sites without differentiation. The details of this calculation are based on measurements that will be discussed later, including the average nanosheet size measured by transmission electron microscopy and the saturation concentration and location of Co on the surface of MoS_2_. The details of this computation be found in the “Turnover Frequency Computation” section of the SI. Based on this computation, it further clarifies the maxima around 21% Co:Mo in thiophene conversion. However, these methods thus far do not fully demonstrate the role that Co plays in modifying the reaction rate of MoS_2_ catalysts. To grasp this, we must differentiate the intrinsic activity of MoS_2_ from that of Co.

To effectively compare samples by Co content, we took the activity difference between each Co-doped sample and nanoscale MoS_2_ without Co and normalized it to its respective Co concentration, as seen in Figure 1g. We calculated a Co-specific TOF improvement rate using this approach since both Co and the MoS_2_ edges are possible active sites; simply attributing all activity to Co would have artificially increased the TOF per unit Co at lower Co loadings by undercounting active sites. Instead, we calculated the difference in thiophene conversion between each Co-MoS_2_ catalyst with the 0% Co:Mo catalyst and divided it by the Co content (see Section “Turnover Frequency Computation” in SI for calculation details). This approach does not calculate TOF per unit Co atom but rather calculates the relative improvement per unit Co atom by removing the intrinsic activity of MoS_2_ from the comparison. By doing so, we remove from the equation the moles of Mo-edge and basal atoms, the latter of which are inert and dilute the overall TOF per unit mole. This is to provide a better comparison of activity between Co positions, which as we shall show is dependent on the Co loading. Interestingly, the maximum value at 21% Co:Mo implies that the Co atoms in this catalyst is more active than in the 10% Co:Mo catalyst, per unit Co atom. This observation opens the intriguing prospect of not only a concentration effect but also a positional change of Co that increases its per-atom activity, thus requiring an in-depth study of the MoS_2_-dopant structure, which we discuss further in the next section.

### Structure of Co-MoS_2_ Nanosheets

**Figure 2:**
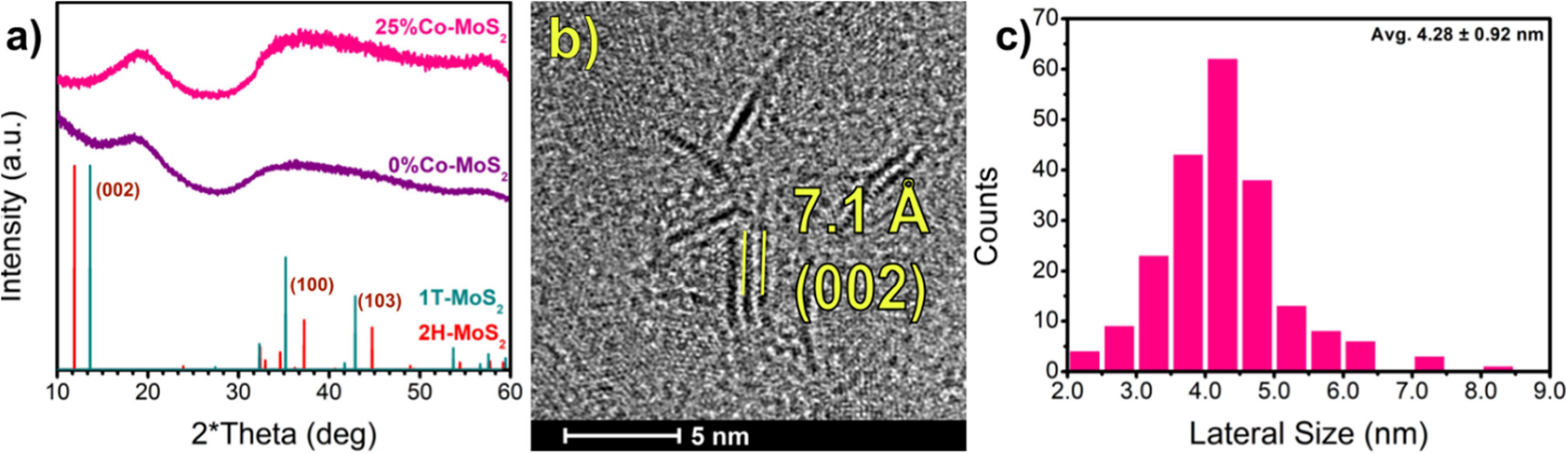
(a) X-ray diffraction patterns of pre-HDS MoS_2_ at various Co loadings. (b) High-resolution transmission electron microscopy 21% Co:Mo after use in HDS, showing the 7.1 Å interlayer distance of the (002) plane and nanosheets do not agglomerate under reaction conditions. Size distribution of the sample is shown in (c) with a sample size of 210 nanosheets.

To determine any structural or morphological differences in the synthesized nanosheets that may suggest this peak in catalytic activity, we first employed powder X-ray diffraction (p-XRD) and high-resolution transmission electron microscopy (HRTEM), the results of which are featured in Figure 2. From XRD, the broad peaks typical of small, amorphous nanostructures due to Scherrer broadening are present and do not necessarily indicate that the nanosheets are amorphous. The XRD patterns were compared with references of the metastable, metallic trigonal 1T and stable, semiconducting hexagonal 2H phase.^47,48^ The reference patterns are taken from Materials Project.^49^ The primary (002) peak is missing from the as-synthesized nanosheets, indicating that there is little to no stacking along the vertical axis, and these are primarily single- to few-layer sheets. Instead, a new peak appears at 19°, which has been observed in prior literature and typically attributed to (004) in 1T-MoS_2_ preparations.^50,51^ The normalized intensities of these broad peaks are made more prominent by the lack of a strong (002) peak. However, it is difficult to fully determine the phase solely from the XRD data due to the lack of defining features.

Although we cannot specify the phase of the fresh catalyst from XRD alone, we can notice other important details about the structure. No diffraction peaks from Co metal or cobalt sulfides appear, suggesting that Co was completely incorporated into the MoS_2_ structure with no phase separation. We also do not observe the formation of any major segregation between Co and Mo at the micrometer scale in scanning electron microscopy–energy dispersive X-ray spectroscopy (SEM–EDX) (see Figure S4 in SI). Using atomic force microscopy (AFM), the measured nanosheets had a height of 20–30 Å, equal to 3–4 MoS_2_ layers (see Figure S5 in SI). The appearance of (100) and (103) peaks in XRD of post-HDS catalysts indicates the structure conformed to the 2H phase during operation (see Figure S2 in SI).^47^ After use in HDS, there is little evidence of agglomeration of the nanosheets as they maintain their nanoscale form with minimal stacking and no agglomeration of Co was observed. Figure S2 also shows the small appearance of the (002) interlayer peak post-HDS. This correlates with the small amount of stacking in HRTEM of the post-HDS catalyst in Figure 2b, which measures to the expected interlayer distance of 7.1 Å. HRTEM images show that the nanosheets tended to be very small even after use in HDS, measuring roughly 4–5 nm in diameter (Figure 2b,c). The population count in Figure 2c is taken from a total of three populations shown in Figures S31–S33 in the SI, which shows the generally homogeneous size distribution of these nanosheets. Thus, while we cannot identify the phase of freshly synthesized MoS_2_ in this manner, we observe a minimal change in the structure by the addition of Co. Post-HDS, the structure is consistent with the 2H phase across all samples, and neither the Co nor the MoS_2_ nanosheets themselves agglomerate. Further characterizations included in the SI comprise SEM–EDX (Figure S4), thermogravimetric analysis (Figure S6), Fourier transform infrared spectroscopy (Figure S29), and additional HRTEM characterization (Figures S30–S33).

**Figure 3:**
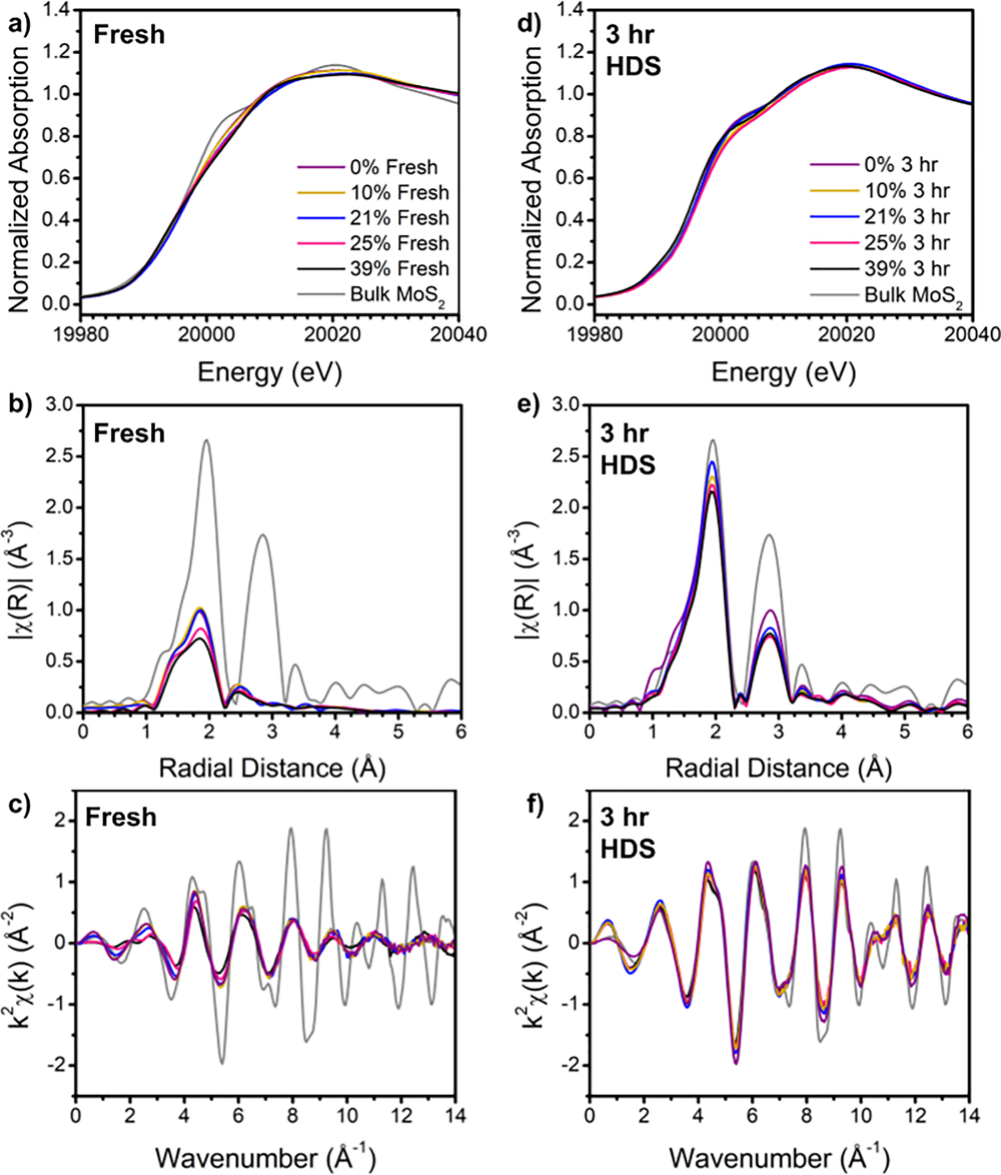
X-ray absorption data collected at the Mo K-edge: (a) XANES of the fresh catalyst, (b) Fourier transform magnitudes of k^2^-weighted EXAFS spectra of the fresh catalyst showin in (c). (d) XANES of the catalyst post-HDS (3 h of use), (e) Fourier transform magnitudes of k^2^-weighted EXAFS spectra of catalyst post-HDS shown in (f).

Given the difficulty in verifying the structure from the broad peaks in XRD and the relatively consistent morphology across the sample space, we employed XAS to observe the finer local structure of the Mo species. As our XRD peaks did not strongly match to any expected MoS_2_ phase, this study allowed us to better understand the initial MoS_2_ structure and phase, if any, as well as the impact of use in HDS on the structure. Figure 3 shows XAS data collected at the Mo K-edge, both for the as-synthesized and post-use samples in HDS, with their X-ray absorption near-edge structure (XANES) and extended X-ray absorption fine structure (EXAFS) in *k-* and *R-*space. Interestingly, the pre-HDS XANES spectra generally did not match the bulk MoS_2_ reference material while the post-HDS spectra did; however, the pre-HDS XANES shows Co influence (particularly 19,998–20,006 eV, Figure 3a). This influence shows relatively better agreement at lower Co loadings than at higher ones, but the general features of bulk MoS_2_ at 20,002 and 20,020 eV are not present in the as-prepared catalysts. Recall that the XRD spectra of the pre-HDS samples also did not match the bulk 2H phase, while the post-HDS spectra did. This mismatch is more apparent in *k-*space (Figure 3c,f) and *R-*space (Figure 3b,e) data, which offer a good agreement with the reference post-HDS but deviate significantly in the fresh material. The post-HDS measurements agreed with the bulk MoS_2_ structure, in which the first *R-*space shell peak is attributed to the Mo–S distance and the second major peak is the Mo–Mo distance. As such, the data fit this structure when it is modeled in *R-*space. The as-synthesized catalyst, however, agreed with the Mo–S peak but exhibited a shortened and reduced second peak. We attribute this structural difference to the *distorted* 1T phase of MoS_2_, in which the Mo–Mo coordination reduces and the interatomic distance shrinks as the crystal distorts to accommodate this metastable state. This distorted phase has been observed in previous literature on colloidal synthesis (and other synthesis methods) of MoS_2_ nanosheets^48,52,53^ and may be ascribed to the reducing environment of the oleylamine ligands. The 1T phase, while metallic and more favorable for catalysis, reverts to the more stable and semiconducting 2H phase at elevated temperatures. Thus, we focus primarily on the structure measured in the post-HDS catalyst as the active species in the reaction. This is confirmed by observing the time-resolved EXAFS of the Mo K-edge (see Figure S12 in the SI), where we observe that the structure rapidly changes from distorted 1T- to 2H-MoS_2_ similar to the bulk reference while heating to the reaction temperature of 300 °C.

In stability testing, the catalyst showed excellent reusability, even after three runs (see Figure S9 in SI). Catalytic activity was preserved, showing no deactivation during use and demonstrating the stability of these catalysts. This observation agrees with the limited stacking of sheets in XRD, the retention of the nanoscale regime observed in the transmission electron micrographs (TEM), and minimal structural change in time-resolved XAS observations (see Figures S10 and S13 in SI).

X-ray photoelectron spectroscopy (XPS) was performed on both fresh and used catalysts to verify the findings in XAS and to further observe the sulfur electronic state, using the two highest Co loadings (25, 39%) to maximize the signal from the Co-edge. Due to the obscuring nature of the amorphous surface ligands, we pretreated these samples with nitrosyl tetrafluoroborate in order to remove the organic ligands (see Section “Ligand Removal Procedure on Nanoscale MoS2” in SI). With ligands present, the signal was muted; this ligand removal treatment was performed only for XPS samples and is not used on any other samples in this work. We did not observe any change in the structure after ligand removal.

**Figure 4:**
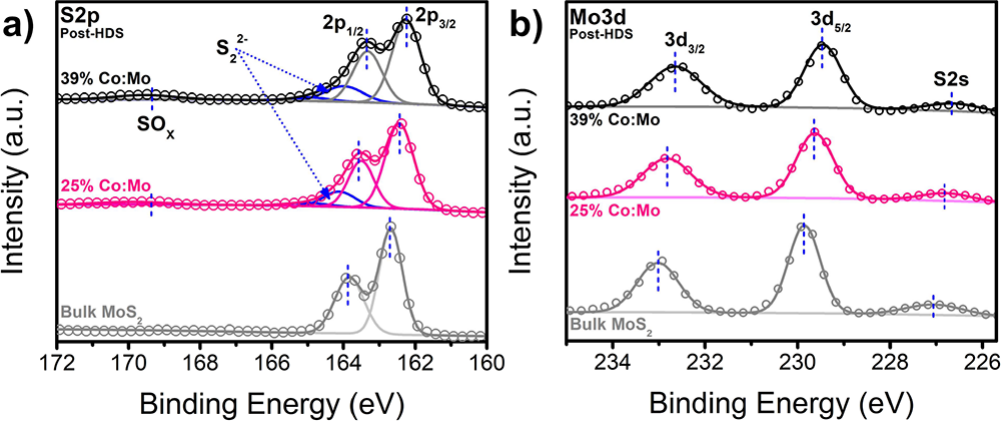
XPS of (a) S-edge and (b) Mo-edge for 25 and 39% Co:Mo post-HDS. Bulk MoS_2_ reference height is multiplied by 0.5 for comparison.

In both fresh and post-HDS catalysts, the S 2p orbital (Figure 4a) shows a slight redshift from bulk MoS_2_. The greater redshift in the fresh S 2p peaks is attributed to the metallic 1T phase and the presence of Co doping. Meanwhile, the shift in the post-HDS catalysts likely arises just from the Co contribution, as further Co addition increases the magnitude of redshift. The ratio of peak height between 2p_1/2_ and 2p_3/2_ is observed higher in the fresh catalyst (Figure S27 in SI) than in the post-HDS catalyst. A similar phenomenon has been observed in prior literature, attributing the increased height of the higher-energy peak to the formation of disulfides (S_2_^2–^).^48,54^ Seo et al. in particular have previously noted these peaks appearing in mono- and few-layer MoS2, with a greater disulfide ratio as the number of layers decreases.^54^ These factors may contribute to the observed apparent peak ratios deviating from the expected spin–orbital splitting ratio of 1:2, which is especially evidenced in the fresh catalyst. Additionally, a slight oxidation peak is present at ∼169 eV, which may arise from residual oxygen- or nitrogen-anchored ligands present on the surface. We also note a redshift in the Mo-edge 3d_5/2_ and 3d_3/2_ peaks (Figure 4b) compared to bulk MoS_2_, and further redshifting as Co loading increases. The fresh catalyst also shows a similar redshift in the Mo-edge (Figure S26 in SI), which indicates that the as-synthesized 2D-TMDs may exhibit the expected metallic distorted 1T phase.^48,55^ By observing the XPS survey scan, we did not detect any additional species that may be present as contaminants, such as chlorine.

We have confirmed the successful synthesis of nanosheets but have not identified any structural changes in the MoS_2_ lattice attributable to the optimal catalytic activity observed at 21% Co:Mo. The distorted metallic 1T phase is not retained under reactor conditions by the addition of Co. XAS and XPS both showed a reduction in the oxidation state of Mo as Co loading increases. As the nonlinear activity was likely derived from the role of Co in the structure, we found it necessary to probe the Co local structure.

### Co Local Structure and Location

**Figure 5:**
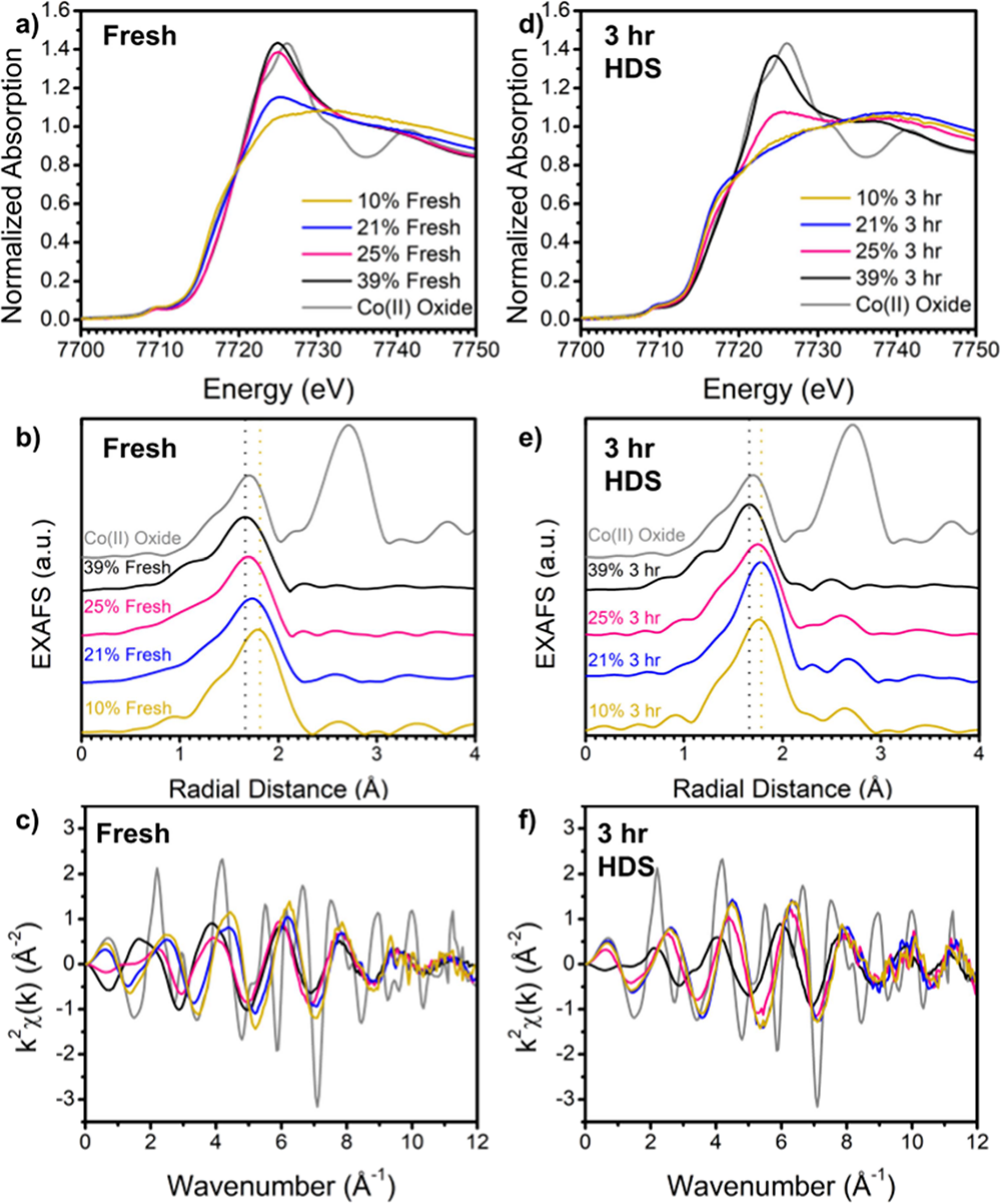
X-ray absorption data collected at the Co K-edge: (a) XANES of the fresh catalyst, (b) Fourier transform magnitudes of k^2^-weighted EXAFS spectra of the fresh catalysts shown in (c). (d) XANES of the catalyst post-HDS (3 h of use), (e) Fourier transform magnitudes of k^2^-weighted EXAFS spectra of catalyst post-HDS shown in (f). A phase transition is observed before and after use particularly at 21 and 25% Co:Mo, with a bond length increase and phase shift in *k-*space at 25% Co:Mo after use.

To study the local bonding environment of Co atoms in Co-MoS_2_, we first extended our XAS analysis to the Co K-edge. First, the Co K-edge XANES (Figure 5a,d) showed a transition between two apparent Co states, as indicated by the blue-shift in absorption energy and increase in white line intensity as the Co concentration increases. The presence of quasi-isosbestic points at 7719 and 7730 eV implies that a transition exists predominantly between only two states. This observation agreed with the shortening of the primary Co–S bond length in EXAFS (Figure 5b,e) as well as the phase shift in k-space (Figure 5c,f), implying a change in the generalized local structure of Co as a function of the Co concentration. In 25 and 39% Co:Mo samples especially, we note that the Co bond length approaches that of Co–O in cobalt(II) oxide. However, while little changed in the Co structure at low Co concentration, the absorption energy redshifted at higher Co concentrations between fresh and post-HDS catalysts. At 21 and 25% Co:Mo, in particular, the absorption energy redshifts post-HDS and the Co bond length increased, in agreement with expected Co–S length than Co–O. With the exception of 39% Co:Mo, samples appeared in-phase in k-space. EXAFS of Co-MoS_2_ did not indicate any Co–Co bonding, confirming that Co was present as isolated atoms and not in clusters. Although the classic CoMoS structure can be synthesized by substituting edge Mo atoms with Co atoms,^56^ we do not expect this case due to the minimal appearance of a second-shell peak, showing that Co atoms were uniformly dispersed with only minimal interaction with Mo atoms.

**Figure 6:**
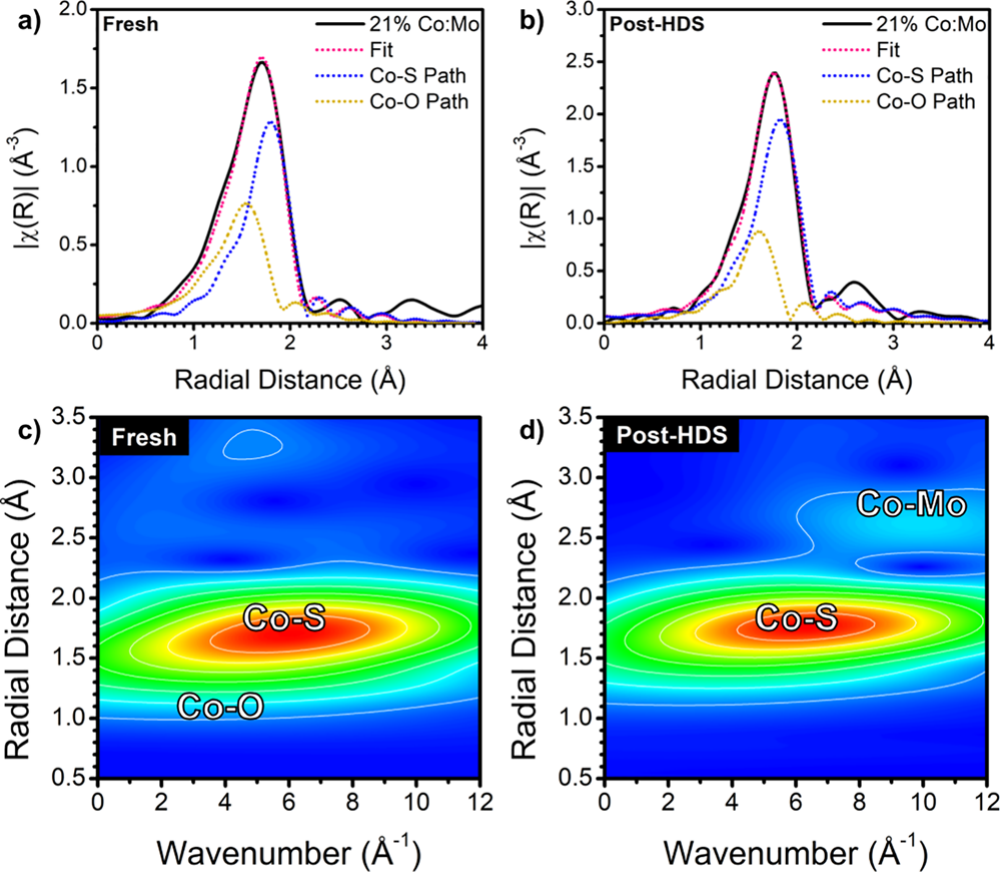
Fourier transform magnitudes of the k^2^-weighted data and fits for the Co K-edge EXAFS of (a) fresh and (b) post-HDS 21% Co:Mo samples fitted with Co–S and Co–O paths. Wavelet transforms of (c) fresh and (d) post-HDS 21% samples.

To further elucidate the origin of the peaks observed in EXAFS, we modeled the data in R-space and performed a wavelet transform (WT) to investigate the Co–S and Co–O contributions. This approach also allowed us to compute the ensemble-averaged bond lengths observed in each sample, which, as shown in Figure 5b,e, vary with Co concentration. WTs were calculated using methods adapted from Muñoz et al.^57^ Employing WTs and modeling the EXAFS data allow differentiation of the Co bonding species, coordination, and bond length. We see by comparing the WTs of fresh 21% Co-MoS_2_ with literature and cobalt oxide (see Figure S24 in SI) that, although the bond length in EXAFS was closer to Co–O than Co–S, there is a combined contribution of O and S present.^58,59^ This is denoted in Figure 6c by the two features at 4 and 6 Å^–1^ (radial distances of 1.3 and 1.7 Å), respectively. The Co–O feature is more pronounced in catalysts of higher Co loading, such as that seen in 25% Co-MoS_2_ (Figure S25 in the SI). Post-HDS, the Co–O contribution to the bond lengths was reduced and a new feature appeared at 9 Å^–1^. This feature was also present in other samples post-HDS except for 10% Co-MoS_2_. This peak did not match Co–Co bonding during EXAFS modeling, and we instead attribute it to the Co–Mo path expected in Co doping atop the basal plane.^58^ To further elucidate the structure, we modeled the EXAFS data by individual species contributions (Figure 6a,b). Both Co–O and Co–S contributed to the fitting of the first shell for all of the species. However, the ratio of Co–O to Co–S coordination and their subsequent bond lengths varied as a factor of Co concentration, in both fresh and post-HDS samples. At low Co concentrations, while the Co–S contribution dominated; the Co–O contribution increased as the amount of Co increased. As evidenced by the isosbestic point in the Co XANES region, Co transitions between states of Co–S and Co–O dominance. We believe that the Co–O contribution originated from the ligands present on these nanosheets; catalysts prepared and measured in an air-free environment had a structure similar to samples measured in air. While it is difficult to differentiate between neighboring atoms of similar atomic number (O, N) in EXAFS modeling, we presume oleic acid to be the dominant ligand species on Co owing to their abundance and presence with CoCl_2_ during synthesis, likely proceeding to the formation of a Co-oleate complex prior to precursor injection in the reaction mixture. However, Co–S was dominant in all catalysts after use in HDS, even while the structure was maintained, especially at high Co concentrations. Furthermore, the appearance of the Co–Mo feature in the second shell leads us to believe that Co was affixed to the basal plane. Co–S coordination was greater than the expected value of three for surface-bound Co, which we attribute to the sulfidation of Co under HDS conditions. Detailed tables of fitting parameters and additional R-space and k-space fit plots are included in Tables S1–S3 and Figures S14–S23 in the SI.

**Figure 7:**
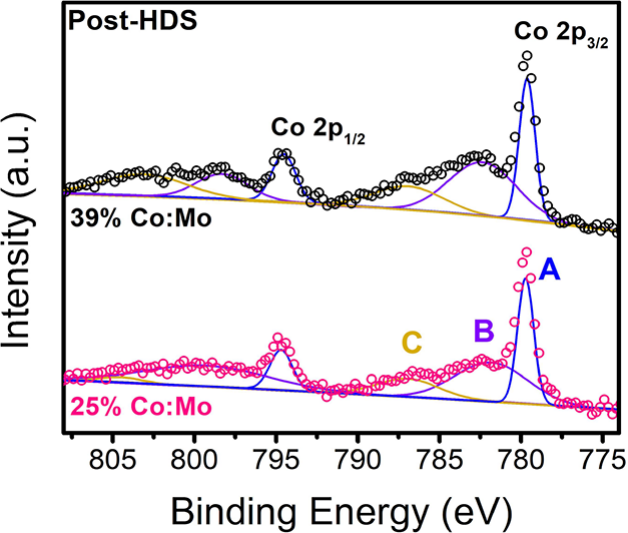
Fitted Co XPS of post-HDS 25 and 39% Co:Mo. (A) CoMoS phase, ∼779 eV; (B) Co oxides, ∼782 eV; (C) saturation peaks.

To further probe the state of Co incorporated into MoS_2_, we employed XPS of the Co 2p orbital. Due to the high dilution of Co atoms on the surface of MoS_2_, only the two highest Co loadings were measured to maximize the signal. By observing the Co 2p orbital, we further elucidated the Co electronic structure observed in the nanosheets. The peak shapes for the fresh catalysts were broad and low in intensity, attributed to the presence of a mixture of Co^2+^ and Co^3+^ states by comparison to literature spectra (Figure S28, SI).^38,48,60−62^ This is potentially the result of the remaining Co-oleate complexes present on the surface of the fresh catalyst. The post-HDS sample (Figure 7), however, presents a much sharper peak (denoted “A”) at ∼779.6 eV, suggesting primarily a Co^3+^ state. The Co^2+^ contribution was still slightly present in the tailing peak at ∼783 eV (“B”). The shift in contribution to primarily Co^3+^ is consistent with Co bound to the sulfur surface, as has been previously reported, particularly in relating the adsorption of single Co atoms on the surface of MoS_2_.^21,38,48,63,64^ The absence of metallic Co (∼778 eV) indicates that no Co metal nanoparticle clusters appear to form post-HDS. This supports the lack of Co–Co bonding observed in XAS and WT (Figure 6), as well as the lack of Co metal peaks observed in XRD (Figure 2a).

**Figure 8:**
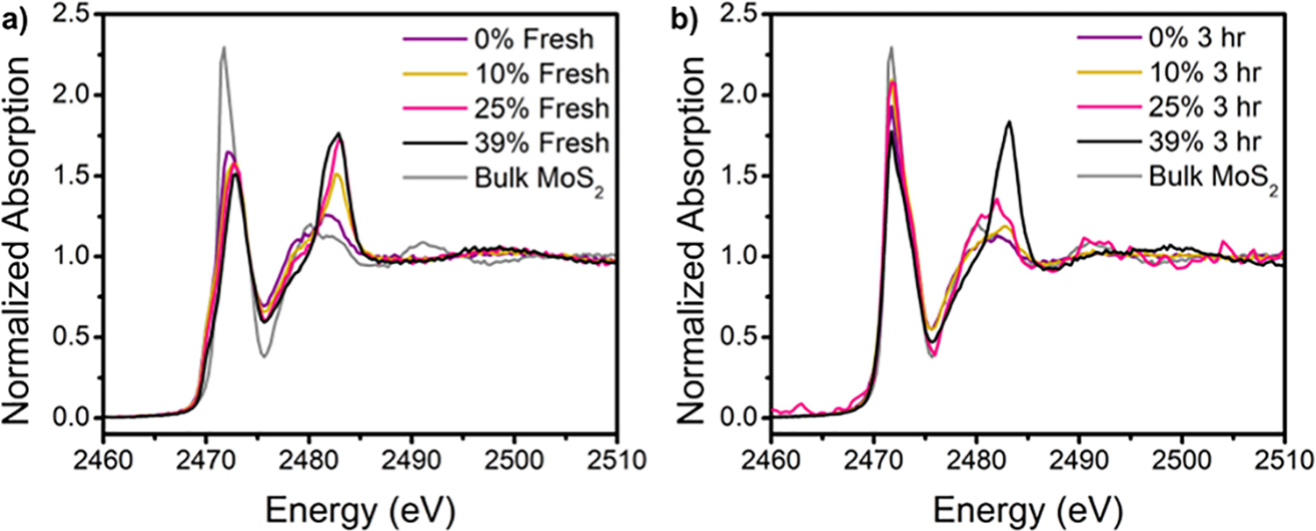
Sulfur K-edge XANES of (a) fresh and (b) post-HDS material.

Up to this point, we have experimentally observed the shifting influence of O and S as Co-oleate complex morphs into Co atoms on the surface of MoS_2_. However, while we can identify the presence of Co-oleate and MoS_2_ before use in HDS as well as the influence of Co addition on the Mo state, we have yet to show the indication that Co-oleate attaches to the sulfur surface during the initial synthesis. Figure 3a reveals no change in the oxidation state of sulfur, and it is difficult to discern from XPS alone how Co and Co-oleate influence the sulfur local structure. To study this, we employed XAS at the sulfur K-edge to probe its local environment (Figure 8). At the S K-edge XANES of fresh material, the first peak at ∼2472 eV was generally redshifted from bulk MoS_2_, but blueshifted among the nanoscale samples as Co loading increased. This peak can be attributed to the S^2–^ state present in MoS_2_. We assign this peak shift and broadening to the presence of the 1T phase, as previously reported.^65^ An additional peak begins to appear as Co loading increases at ∼2484 eV. This peak is present with a high level of oxidation of sulfur, up to S^6+^. This is likely due to the presence of oleate-liganded Co complexes attaching to the surface as we initially hypothesized.

The catalysts post-HDS align much more clearly with the bulk MoS_2_ reference (*vide supra*). The secondary peak disappears for 10 and 21% Co:Mo and is significantly reduced for 25% Co:Mo. However, this complex is still present in 39% Co:Mo. Additionally, the peak location no longer shifts as a function of the Co loading. If this secondary peak indeed arises from the Co-oleate complex, it suggests the existence of a crowding effect. At higher Co loadings, not all Co atoms fully attach to the surface, and some may remain as a Co-oleate complex, precluding active sites. The lack of Co influence on the peak shift in the post-HDS catalyst further supports the notion that Co does not play any influence on the structural phase of MoS_2_. Additionally, at high Co loading, these oleate complexes are still present and are detrimental to the catalytic activity. This implies that these Co-oleate complexes may remain on the surface even during HDS if there is not enough space to support more Co atoms on the basal surface, thus impeding the activity.

In summary, the doping of MoS_2_ with Co plays a significant role in the overall catalyst structure and the mechanism by which Co atoms attach to MoS_2_. Furthermore, the catalytic activity is tuned by tweaking the Co dopant concentration. At low concentrations, the structure of Co observed in fresh and post-HDS catalysts shows little difference and suggests the direct formation of Co_*x*_MoS_2_. Meanwhile, at higher concentrations, Co-oleate complexes are observed, suggesting that the first atoms of Co (i.e., at low concentrations) fix to a different location than Co atoms at higher concentrations. These complexes interact directly with the surface of MoS_2_, either through the Co–S bond, van der Waals forces between Co-oleate and the MoS_2_ basal plane, or both. Post-HDS, catalysts exhibit similar structures regardless of Co concentration, indicating that the Co-oleate complexes are transformed to Co_*x*_MoS_2_. To shed more light on the observed differences between Co concentrations, we performed DFT calculations to compare what ought to be expected for this system with what was observed experimentally, and the driving factors for dopant location and for charge transfer between Co and Mo. By studying the underlying mechanisms, we can better develop the rules for the design of doped TMD catalysts.

### DFT Calculations

**Figure 9:**
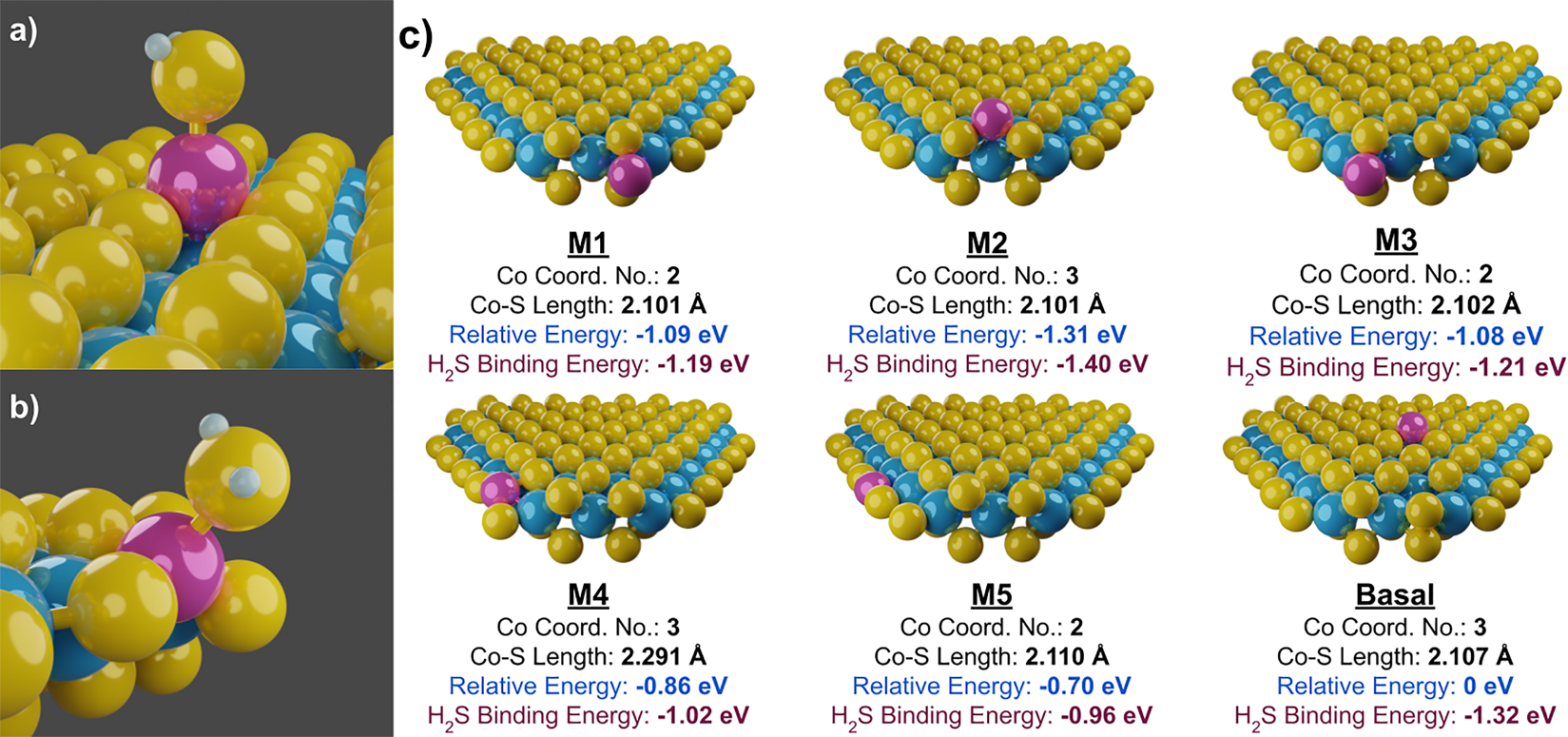
Predicted H_2_S adsorption structures for (a) basal Co and (b) M4 Co positions, and (c) expected Co dopant locations with relevant computed DFT results (Co–S coordination, Co–S bond length, the energy of Co at different positions relative to Co located on the basal plane, and H_2_S binding energy).

Through DFT calculations, we first examined the location and preference for binding sites of the Co atoms that decorate the MoS_2_ nanosheets. The 2H phase of MoS_2_ is used for this computation, as it is the stable, active catalyst structure that supports Co atoms under reaction conditions. By computing a variety of positions for a single Co atom along the Mo-edge, S-edge, on the corners, and atop the basal plane, we determined the general preference for Co atom location. The energy of each position was set relative to the Co atom located on the basal plane. Examining the relative energies, we observed that Co has a strong preference for the corner sites of the MoS_2_ slab, particularly along the S-edge (Figure 9c). This is followed by edge-doping along the metal edge, which has fewer S atoms. These positions are all favored over the basal plane, indicating that Co, in general, would prefer to bind to the nanosheet edges. Although Co atoms will first dope the edges, the limited edge area and minimal corner sites available relative to the size of the nanosheets implies that these sites may saturate quickly as the Co:Mo ratio increases, thus allowing Co to begin doping the basal plane past a certain concentration threshold.

We then took these computed structures and measured their interaction with H_2_S as an analogue material for S-adsorption (see structures in Figure S35 in the SI). H_2_S was chosen in place of thiophene both to simplify the calculations and to observe how readily the H_2_S product desorbs after the reaction. While basal Co is the least stable structure, it adsorbs H_2_S well compared to edge-doped sites, surpassed only by the sparing corner sites. Interestingly, the remaining sites tend to follow a trend converse to basal Co, where decreasing H_2_S adsorption correlates to decreasing Co-MoS_2_ stability. This implies that not only would Co atop the inert basal plane create new active sites on MoS_2_, but the sites would themselves be more active at adsorbing sulfur. This creates a trade-off: the preference of H_2_S to adsorb to basal Co would lead to a slower desorption of H_2_S products. The difference between 21 and 25% Co:Mo HDS activity may be attributed to the greater initial formation of H_2_S poisoning the additional active basal Co sites of the latter. This propensity for H_2_S to bind to the surface of Co also agrees with the excess Co–S coordination found with EXAFS modeling (see Table S3 in the SI), implying the Co is further sulfided under reaction conditions.

### Local Structure of Co Atoms

Combining the structural and electronic computations with our experimental observations, we can develop a better model of how Co loading influences the Co location and attachment mechanism on MoS_2_ for this system. From our TEM measurements (Figure 2b), we calculate that the nanosheet edges comprise approximately 32% of the total area, assuming a truncated triangular morphology (see Figure S34 in the SI for calculation details). If the edges were to be completely doped with Co without basal plane coverage, this would result in a Co:Mo ratio of ∼32%. However, we note both structural and activity changes as low as 21%, indicating edge saturation occurs even before the entire edge has been filled. We attribute this nominally to steric hindrances introduced by oleate ligands on Co, which act as directing agents to prevent crowding of Co along the edges and to activate basal plane doping at lower Co:Mo ratios. Additionally, electrostatic repulsions between adjacent Co atoms along the edges may play a role in the saturation of the edge at lower ratios than what is expected from simple geometric calculations. We demonstrate using XAS and XPS (Figures 3 and 4) that the Mo oxidation state reduces with increasing Co concentration and a local charge state change which would make nearby doping with more Co difficult. It is likely a complex combination of factors, but we can conclude that the dopant saturation limit of the nanosheet edges is far less than the number of possible edge sites. In our calculations, we also consider a hexagonal structure (see Table S4 in SI), although we do not observe this in TEM.

**Figure 10:**
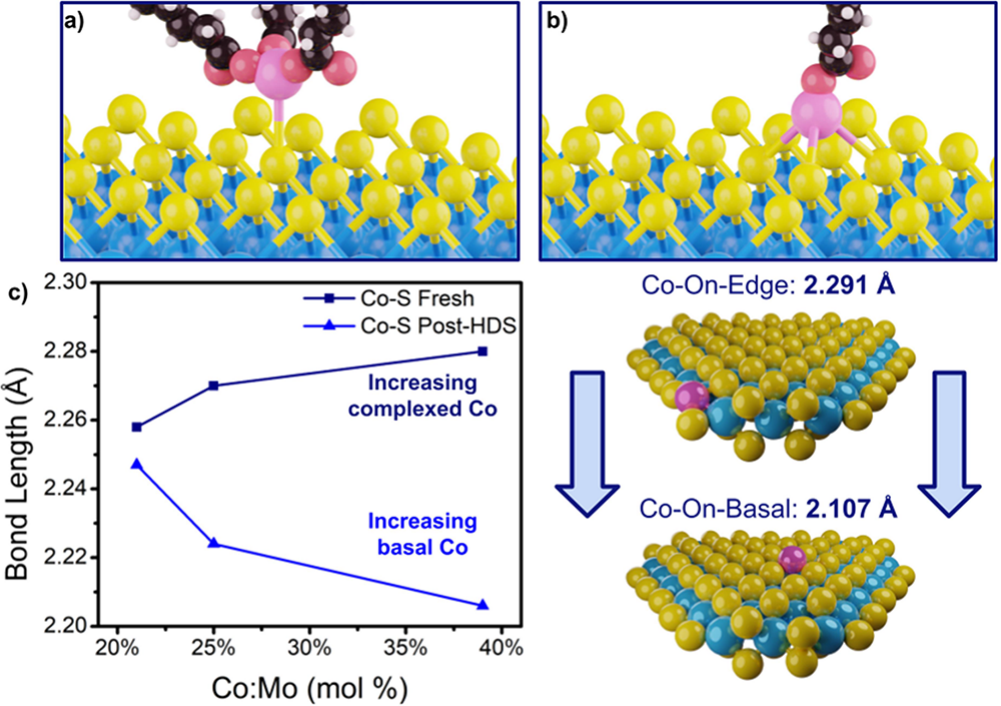
3D models depicting (a) Co-oleate complex attaching to basal plane and (b) Co single-atom site affixed to basal plane. (c) EXAFS modeling results at the Co *K-*edge for the Co–S path length of varying Co loadings before and after use in HDS, compared to modeled lengths from DFT calculations.

Based on these findings, we propose that Co prefers to adsorb onto the edge of MoS_2_, which does not add new active sites and only replaces the active edge sites. Based on the average nanosheet size, however, we calculate that at Co:Mo atomic ratios greater than 16%, the edges become saturated. Any additional Co then partially binds to the basal plane as Co-oleate complexes, which under the reaction conditions are significantly reduced to Co atoms atop the basal plane. A similar mechanism has been proposed by Hong et al. for ligand-directed Co doping of WS_2_, forming organocobalt molecules on the basal plane that are further reduced by 300 °C sulfidation.^66^ This is evident in both the increase in Co–S coordination and the trends in Co–S radial distance observed in FT-EXAFS (Figure 10c); Co–S length increases as more Co-oleate complexes attach to the surface but decreases as more Co fixes to the basal plane. However, Figure 8a implies that Co-oleate is interacting with sulfur, whether through surface bonding between S and O or through van der Waals forces keeping Co-oleate on the surface. Additionally, we see agreement between measured bond lengths and those predicted by DFT, as average Co–S length trends downward with the increase in basal-fixed Co atoms.

We additionally propose that there is a performance limit through this doping practice as activity decreases beyond 21% Co:Mo. It is likely difficult, given steric and electronic hindrances, for a large amount of Co to populate the basal plane. Therefore, we see that while 21% Co:Mo performs best by the addition of new active sites on the basal plane, adding further Co (25, 39% Co:Mo) does not improve and in fact decreases. This explains the presence of higher oxygenation of Co observed in the post-HDS catalyst—the excess Co can only partly bind to the surface and is complexed with oleic acid ligands (the source of the Co–O bond observed). These ligands add steric hindrance to the reaction mechanism, thus, inhibiting further improvements. Crowding the basal plane with Co atoms reduces their dispersion, which is known to inhibit reaction kinetics in single-atom catalysts.^67^ It should be noted that these ratios are dependent upon sheet size; for larger sheets, more Co would be necessary to saturate the nanosheet edges but the basal plane would have a higher crowding tolerance.

Overall, this positional effect of Co concentration explains the increased per-atom activity of the 21% Co:Mo catalyst relative to the others. Basal-doped atoms below a crowding threshold are more active than edge-doped atoms, which, in turn, are more active than undoped sites. Additionally, there is a trade-off for basal Co atoms, as their strong H_2_S affinity may limit product desorption. This helps to outline the rules governing doping of ultrasmall MoS_2_ nanosheets with Co—to fully activate the basal plane, edge-doping must be overcome. While this is less of an issue for larger nanosheets in the 100+ nm range, maximizing active sheet surface area using nanosheets in the 5 nm regime will require optimization to overcome this threshold.

## Conclusions

Colloidal MoS_2_ nanosheets doped in situ with various loadings of single Co atoms have been successfully synthesized to study the structure–activity relationship among Co local structure, positional effect, and HDS activity. While the as-synthesized nanosheets exist in a metastable distorted 1T phase, during reaction conditions, they transform to the more stable 2H bulk phase. Using spectroscopy techniques, we have determined that nanosheet size and Co concentration each play a crucial role in directing the location of Co and its subsequent catalytic behavior. Co prefers to adsorb along the nanosheet edges; once a saturation point is reached, adsorption on the basal plane becomes favorable. Basal-doped Co atoms are more active than their edge-doped counterparts but are prone to surface crowding, yielding optimal activity around 21% Co:Mo. Thus, to activate the basal plane of MoS_2_ and create more active Co species, the threshold of edge-doping must be overcome. We also observe that the edge saturation point of MoS_2_ is less than the available number of edge sites, possibly dictated by steric or electrostatic hindrances. While Co-doped MoS_2_ edges are more catalytically active than undoped edges, the Co atoms on the basal plane are demonstrably more active than edge-doped Co. This optimum and the details of these findings provide insight into how doped TMDs behave in hydrogenolysis and can inform the design of next-generation catalyst materials tailored to respective applications. While these results are specific to Co-doped MoS_2_, the analyses we demonstrated are a guideline that can be extended to other 2D TMD materials and dopants to help realize new phenomena in catalytic activity.

## Supporting Information Available

Supplemental X-ray diffraction data; scanning electron microscopy; atomic force microscopy; thermogravimetric analysis; expected reaction scheme in HDS; bulk MoS_2_ activity and catalyst stability testing data; supplemental X-ray absorption spectroscopy data, EXAFS peak fitting, and wavelet transform data; fresh catalyst X-ray photoelectron spectroscopy; Fourier transform infrared spectroscopy; supplemental high-resolution transmission electron microscopy and population counts; and details on computational models and site count estimations (PDF)

## References

[ref1] Tanimu A.; Alhooshani K. Advanced Hydrodesulfurization Catalysts: A Review of Design and Synthesis. Energy Fuels 2019, 33 (4), 2810–2838. 10.1021/acs.energyfuels.9b00354.

[ref2] Jin W.; Pastor-Pérez L.; Shen D.; Sepúlveda-Escribano A.; Gu S.; Ramirez Reina T. Catalytic Upgrading of Biomass Model Compounds: Novel Approaches and Lessons Learnt from Traditional Hydrodeoxygenation - a Review. ChemCatChem. 2019, 11 (3), 924–960. 10.1002/cctc.201801722.

[ref3] Klimov O. V.; Nadeina K. A.; Vatutina Y. V.; Stolyarova E. A.; Danilova I. G.; Gerasimov E. Y.; Prosvirin I. P.; Noskov A. S. CoMo/Al_2_O_3_ hydrotreating catalysts of diesel fuel with improved hydrodenitrogenation activity. Catal. Today 2018, 307, 73–83. 10.1016/j.cattod.2017.02.032.

[ref4] Cao J.; Zhang Y.; Wang L.; Zhang C.; Zhou C. Unsupported MoS_2_-Based Catalysts for Bio-Oil Hydrodeoxygenation: Recent Advances and Future Perspectives. Front. Chem. 2022, 10, 928806 10.3389/fchem.2022.928806. 35783206 PMC9247250

[ref5] Vrinat M. L. The kinetics of the hydrodesulfurization process - a review. Appl. Catal. 1983, 6 (2), 137–158. 10.1016/0166-9834(83)80260-7.

[ref6] Moses P. G.; Hinnemann B.; Topso̷e H.; No̷rskov J. K. The effect of Co-promotion on MoS2 catalysts for hydrodesulfurization of thiophene: A density functional study. J. Catal. 2009, 268 (2), 201–208. 10.1016/j.jcat.2009.09.016.

[ref7] Zonnevylle M. C.; Hoffman R.; Harris S. Thiophene Hydrodesulfurization on MoS2 Theoretical Aspects. Surf. Sci. 1988, 199, 320–360. 10.1016/0039-6028(88)90415-3.

[ref8] Chianelli R. R.; Berhault G.; Raybaud P.; Kasztelan S.; Hafner J.; Toulhoat H. Periodic trends in hydrodesulfurization: in support of the Sabatier principle. Appl. Catal., A 2002, 227, 83–96. 10.1016/S0926-860X(01)00924-3.

[ref9] Furimsky E. Role of MoS_2_ and WS_2_ in Hydrodesulfurization. Catal. Rev.-Sci. Eng. 1980, 22 (3), 371–400. 10.1080/03602458008067538.

[ref10] Sun Y.; Alimohammadi F.; Zhang D.; Guo G. Enabling Colloidal Synthesis of Edge-Oriented MoS_2_ with Expanded Interlayer Spacing for Enhanced HER Catalysis. Nano Lett. 2017, 17 (3), 1963–1969. 10.1021/acs.nanolett.6b05346. 28186766

[ref11] Zhang X.; Lai Z.; Tan C.; Zhang H. Solution-Processed Two-Dimensional MoS_2_ Nanosheets: Preparation, Hybridization, and Applications. Angew. Chem., Int. Ed. Engl. 2016, 55 (31), 8816–8838. 10.1002/anie.201509933. 27329783

[ref12] Schweiger H.; Raybaud P.; Kresse G.; Toulhoat H. Shape and Edge Sites Modifications of MoS_2_ Catalytic Nanoparticles Induced by Working Conditions: A Theoretical Study. J. Catal. 2002, 207 (1), 76–87. 10.1006/jcat.2002.3508.

[ref13] Baubet B.; Girleanu M.; Gay A.-S.; Taleb A.-L.; Moreaud M.; Wahl F.; Delattre V.; Devers E.; Hugon A.; Ersen O.; et al. Quantitative Two-Dimensional (2D) Morphology–Selectivity Relationship of CoMoS Nanolayers: A Combined High-Resolution High-Angle Annular Dark Field Scanning Transmission Electron Microscopy (HR HAADF-STEM) and Density Functional Theory (DFT) Study. ACS Catal. 2016, 6 (2), 1081–1092. 10.1021/acscatal.5b02628.

[ref14] Lauritsen J. V.; Nyberg M.; Vang R. T.; Bollinger M. V.; Clausen B. S.; Topso̷e H.; Jacobsen K. W.; Lægsgaard E.; No̷rskov J. K.; Besenbacher F. Chemistry of one-dimensional metallic edge states in MoS_2_ nanoclusters. Nanotechnology 2003, 14, 385–389. 10.1088/0957-4484/14/3/306.

[ref15] Yin Y.; Han J.; Zhang Y.; Zhang X.; Xu P.; Yuan Q.; Samad L.; Wang X.; Wang Y.; Zhang Z.; et al. Contributions of Phase, Sulfur Vacancies, and Edges to the Hydrogen Evolution Reaction Catalytic Activity of Porous Molybdenum Disulfide Nanosheets. J. Am. Chem. Soc. 2016, 138 (25), 7965–7972. 10.1021/jacs.6b03714. 27269185

[ref16] Salazar N.; Rangarajan S.; Rodriguez-Fernandez J.; Mavrikakis M.; Lauritsen J. V. Site-dependent reactivity of MoS_2_ nanoparticles in hydrodesulfurization of thiophene. Nat. Commun. 2020, 11 (1), 4369 10.1038/s41467-020-18183-4. 32868769 PMC7459117

[ref17] Liu R.; Fei H.-L.; Ye G.-L. Recent advances in single metal atom-doped MoS_2_ as catalysts for hydrogen evolution reaction. Tungsten 2020, 2 (2), 147–161. 10.1007/s42864-020-00045-7.

[ref18] Zhao Y.; Jiang W. J.; Zhang J.; Lovell E. C.; Amal R.; Han Z.; Lu X. Anchoring Sites Engineering in Single-Atom Catalysts for Highly Efficient Electrochemical Energy Conversion Reactions. Adv. Mater. 2021, 33 (41), e2102801 10.1002/adma.202102801. 34477254

[ref19] Lauritsen J. V.; Vang R. T.; Besenbacher F. From atom-resolved scanning tunneling microscopy (STM) studies to the design of new catalysts. Catal. Today 2006, 111 (1–2), 34–43. 10.1016/j.cattod.2005.10.015.

[ref20] Chen Z.; Liu C.; Liu J.; Li J.; Xi S.; Chi X.; Xu H.; Park I. H.; Peng X.; Li X.; et al. Cobalt Single-Atom-Intercalated Molybdenum Disulfide for Sulfide Oxidation with Exceptional Chemoselectivity. Adv. Mater. 2020, 32 (4), e1906437 10.1002/adma.201906437. 31777990

[ref21] Liu G.; Robertson A. W.; Li M. M.; Kuo W. C. H.; Darby M. T.; Muhieddine M. H.; Lin Y. C.; Suenaga K.; Stamatakis M.; Warner J. H.; et al. MoS_2_ monolayer catalyst doped with isolated Co atoms for the hydrodeoxygenation reaction. Nat. Chem. 2017, 9 (8), 810–816. 10.1038/nchem.2740. 28754945

[ref22] Lau T. H. M.; Lu X.; Kulhavy J.; Wu S.; Lu L.; Wu T. S.; Kato R.; Foord J. S.; Soo Y. L.; Suenaga K.; et al. Transition metal atom doping of the basal plane of MoS_2_ monolayer nanosheets for electrochemical hydrogen evolution. Chem. Sci. 2018, 9 (21), 4769–4776. 10.1039/C8SC01114A. 29910927 PMC5975547

[ref23] Park S.; Park J.; Abroshan H.; Zhang L.; Kim J. K.; Zhang J.; Guo J.; Siahrostami S.; Zheng X. Enhancing Catalytic Activity of MoS_2_ Basal Plane S-Vacancy by Co Cluster Addition. ACS Energy Lett. 2018, 3 (11), 2685–2693. 10.1021/acsenergylett.8b01567.

[ref24] Dai X.; Du K.; Li Z.; Liu M.; Ma Y.; Sun H.; Zhang X.; Yang Y. Co-Doped MoS_2_ Nanosheets with the Dominant CoMoS Phase Coated on Carbon as an Excellent Electrocatalyst for Hydrogen Evolution. ACS Appl. Mater. Interfaces 2015, 7 (49), 27242–27253. 10.1021/acsami.5b08420. 26599427

[ref25] Zhang J.; Du C.; Dai Z.; Chen W.; Zheng Y.; Li B.; Zong Y.; Wang X.; Zhu J.; Yan Q. NbS_2_ Nanosheets with M/Se (M = Fe, Co, Ni) Codopants for Li(+) and Na(+) Storage. ACS Nano 2017, 11 (10), 10599–10607. 10.1021/acsnano.7b06133. 28945352

[ref26] Zhao X.; Zhang X.; Xue Z.; Chen W.; Zhou Z.; Mu T. Fe nanodot-decorated MoS_2_ nanosheets on carbon cloth: an efficient and flexible electrode for ambient ammonia synthesis. J. Mater. Chem. A 2019, 7 (48), 27417–27422. 10.1039/C9TA09264A.

[ref27] Sharma M. D.; Mahala C.; Modak B.; Pande S.; Basu M. Doping of MoS_2_ by ″Cu″ and ″V″: An Efficient Strategy for the Enhancement of Hydrogen Evolution Activity. Langmuir 2021, 37 (16), 4847–4858. 10.1021/acs.langmuir.1c00036. 33844924

[ref28] Rosentsveig R.; Yadgarov L.; Feldman Y.; Shilstein S.; Popovitz-Biro R.; Visic B.; Sedova A.; Cohen S. R.; Li Y.; Frenkel A. I.; et al. Doping of Fullerene-Like MoS_2_ Nanoparticles with Minute Amounts of Niobium. Part. Part. Syst. Charact. 2017, 35 (3), 1700165 10.1002/ppsc.201700165.

[ref29] Yadgarov L.; Rosentsveig R.; Leitus G.; Albu-Yaron A.; Moshkovich A.; Perfilyev V.; Vasic R.; Frenkel A. I.; Enyashin A. N.; Seifert G.; et al. Controlled doping of MS_2_ (M = W, Mo) nanotubes and fullerene-like nanoparticles. Angew. Chem., Int. Ed. Engl. 2012, 51 (5), 1148–1151. 10.1002/anie.201105324. 22213621

[ref30] Qin R.; Liu P.; Fu G.; Zheng N. Strategies for Stabilizing Atomically Dispersed Metal Catalysts. Small Methods 2018, 2 (1), 1700286 10.1002/smtd.201700286.

[ref31] Frenkel A. I.; Wang Q.; Sanchez S. I.; Small M. W.; Nuzzo R. G. Short range order in bimetallic nanoalloys: An extended X-ray absorption fine structure study. J. Chem. Phys. 2013, 138 (6), 064202 10.1063/1.4790509. 23425464

[ref32] Frenkel A. I. Applications of extended X-ray absorption fine-structure spectroscopy to studies of bimetallic nanoparticle catalysts. Chem. Soc. Rev. 2012, 41 (24), 8163–8178. 10.1039/c2cs35174a. 22833100

[ref33] Menard L. D.; Xu H.; Gao S.-P.; Twesten R. D.; Harper A. S.; Song Y.; Wang G.; Douglas A. D.; Yang J. C.; Frenkel A. I.; et al. Metal core bonding motifs of monodisperse icosahedral Au_13_ and larger Au monolayer-protected clusters as revealed by X-ray absorption spectroscopy and transmission electron microscopy. J. Phys. Chem. B 2006, 110 (30), 14564–14573. 10.1021/jp060740f. 16869556

[ref34] Glasner D.; Frenkel A. I. Geometrical Characteristics of Regular Polyhedra: Application to EXAFS Studies of Nanoclusters. AIP Conf. Proc. 2007, 882, 746–748. 10.1063/1.2644651.

[ref35] Routh P. K.; Marcella N.; Frenkel A. I. Speciation of Nanocatalysts Using X-ray Absorption Spectroscopy Assisted by Machine Learning. J. Phys. Chem. C 2023, 127 (12), 5653–5662. 10.1021/acs.jpcc.3c00571.

[ref36] Scimeca M. R.; Mattu N.; Paredes I. J.; Tran M. N.; Paul S. J.; Aydil E. S.; Sahu A. Origin of Intraband Optical Transitions in Ag_2_Se Colloidal Quantum Dots. J. Phys. Chem. C 2021, 125 (31), 17556–17564. 10.1021/acs.jpcc.1c05371.

[ref37] Mo̷lnås H.; Russ B.; Farrell S. L.; Gordon M. P.; Urban J. J.; Sahu A. n-Type doping of a solution processed p-type semiconductor using isoelectronic surface dopants for homojunction fabrication. Appl. Surf. Sci. 2022, 590, 153089 10.1016/j.apsusc.2022.153089.

[ref38] Guo K.; Ding Y.; Yu Z. One-step synthesis of ultrafine MoNiS and MoCoS monolayers as high-performance catalysts for hydrodesulfurization and hydrodenitrogenation. Appl. Catal., B 2018, 239, 433–440. 10.1016/j.apcatb.2018.08.041.

[ref39] Gro̷nborg S. S.; Salazar N.; Bruix A.; Rodriguez-Fernandez J.; Thomsen S. D.; Hammer B.; Lauritsen J. V. Visualizing hydrogen-induced reshaping and edge activation in MoS_2_ and Co-promoted MoS_2_ catalyst clusters. Nat. Commun. 2018, 9 (1), 2211 10.1038/s41467-018-04615-9. 29880841 PMC5992198

[ref40] Kresse G.; Furthmüller J. Efficiency of ab-initio total energy calculations for metals and semiconductors using a plane-wave basis set. Comput. Mater. Sci. 1996, 6 (1), 15–50. 10.1016/0927-0256(96)00008-0.

[ref41] Kresse G.; Furthmüller J. Efficient iterative schemes for ab initio total-energy calculations using a plane-wave basis set. Phys. Rev. B 1996, 54, 11169 10.1103/PhysRevB.54.11169. 9984901

[ref42] Kresse G.; Joubert D. From ultrasoft pseudopotentials to the projector augmented-wave method. Phys. Rev. B 1999, 59, 1758 10.1103/PhysRevB.59.1758.

[ref43] Perdew J. P.; Kieron B.; Ernzerhof Matthias Generalized Gradient Approximation Made Simple. Phys. Rev. Lett. 1996, 77, 3865 10.1103/PhysRevLett.77.3865. 10062328

[ref44] Grimme S.; Antony J.; Ehrlich S.; Krieg H. A consistent and accurate ab initio parametrization of density functional dispersion correction (DFT-D) for the 94 elements H-Pu. J. Chem. Phys. 2010, 132 (15), 154104 10.1063/1.3382344. 20423165

[ref45] van Haandel L.; Smolentsev G.; van Bokhoven J. A.; Hensen E. J. M.; Weber T. Evidence of Octahedral Co–Mo–S Sites in Hydrodesulfurization Catalysts as Determined by Resonant Inelastic X-ray Scattering and X-ray Absorption Spectroscopy. ACS Catal. 2020, 10 (19), 10978–10988. 10.1021/acscatal.0c03062.

[ref46] Al-Zeghayer Y. S.; Sunderland P.; Al-Masry W.; Al-Mubaddel F.; Ibrahim A. A.; Bhartiya B. K.; Jibril B. Y. Activity of CoMo/γ-Al_2_O_3_ as a catalyst in hydrodesulfurization: effects of Co/Mo ratio and drying condition. Appl. Catal., A 2005, 282 (1–2), 163–171. 10.1016/j.apcata.2004.12.021.

[ref47] Bai J.; Zhao B.; Zhou J.; Si J.; Fang Z.; Li K.; Ma H.; Dai J.; Zhu X.; Sun Y. Glucose-Induced Synthesis of 1T-MoS_2_/C Hybrid for High-Rate Lithium-Ion Batteries. Small 2019, 15 (14), e1805420 10.1002/smll.201805420. 30848553

[ref48] Kwon I. S.; Debela T. T.; Kwak I. H.; Park Y. C.; Seo J.; Shim J. Y.; Yoo S. J.; Kim J. G.; Park J.; Kang H. S. Ruthenium Nanoparticles on Cobalt-Doped 1T’ Phase MoS_2_ Nanosheets for Overall Water Splitting. Small 2020, 16 (13), e2000081 10.1002/smll.202000081. 32147958

[ref49] Jain A.; Ong S. P.; Hautier G.; Chen W.; Richards W. D.; Dacek S.; Cholia S.; Gunter D.; Skinner D.; Ceder G.; et al. Commentary: The Materials Project: A materials genome approach to accelerating materials innovation. APL Mater. 2013, 1, 011002 10.1063/1.4812323.

[ref50] Xiang T.; Fang Q.; Xie H.; Wu C.; Wang C.; Zhou Y.; Liu D.; Chen S.; Khalil A.; Tao S.; et al. Vertical 1T-MoS_2_ nanosheets with expanded interlayer spacing edged on a graphene frame for high rate lithium-ion batteries. Nanoscale 2017, 9 (21), 6975–6983. 10.1039/C7NR02003A. 28524923

[ref51] Zhang Y.; Kuwahara Y.; Mori K.; Louis C.; Yamashita H. Hybrid phase 1T-2H-MoS_2_ with controllable 1T concentration and its promoted hydrogen evolution reaction. Nanoscale 2020, 12 (22), 11908–11915. 10.1039/D0NR02525A. 32467961

[ref52] Huang Y.; Sun Y.; Zheng X.; Aoki T.; Pattengale B.; Huang J.; He X.; Bian W.; Younan S.; Williams N.; et al. Atomically engineering activation sites onto metallic 1T-MoS_2_ catalysts for enhanced electrochemical hydrogen evolution. Nat. Commun. 2019, 10, 982 10.1038/s41467-019-08877-9. 30816110 PMC6395606

[ref53] Liu Q.; Fang Q.; Chu W.; Wan Y.; Li X.; Xu W.; Habib M.; Tao S.; Zhou Y.; Liu D.; et al. Electron-Doped 1T-MoS_2_ via Interface Engineering for Enhanced Eletrocatalytic Hydrogen Evolution. Chem. Mater. 2017, 29 (11), 4738–4744. 10.1021/acs.chemmater.7b00446.

[ref54] Seo B.; Jung G. Y.; Sa Y. J.; Jeong H. Y.; Cheon J. Y.; Lee H. J.; Kim H. Y.; Kim J. C.; Shin H. S.; Kwak S. K.; et al. Monolayer-Precision Synthesis of Molybdenum Sulfide Nanoparticles and Their Nanoscale Size Effects in the Hydrogen Evolution Reaction. ACS Nano 2015, 9 (4), 3728–3739. 10.1021/acsnano.5b00786. 25794552

[ref55] Liu Q.; Li X.; He Q.; Khalil A.; Liu D.; Xiang T.; Wu X.; Song L. Gram-Scale Aqueous Synthesis of Stable Few-Layered 1T-MoS_2_: Applications for Visible-Light-Driven Photocatalytic Hydrogen Evolution. Small 2015, 11 (41), 5556–5564. 10.1002/smll.201501822. 26332270

[ref56] Gandubert A. D.; Krebs E.; Legens C.; Costa D.; Guillaume D.; Raybaud P. Optimal promoter edge decoration of CoMoS catalysts: A combined theoretical and experimental study. Catal. Today 2008, 130 (1), 149–159. 10.1016/j.cattod.2007.06.041.

[ref57] Muñoz M.; Argoul P.; Farges F. O. Continuous Cauchy wavelet transform analyses of EXAFS spectra: A qualitative approach. Am. Mineral. 2003, 88 (4), 694–700. 10.2138/am-2003-0423.

[ref58] Li Z.; Li C.; Chen J.; Xing X.; Wang Y.; Zhang Y.; Yang M.; Zhang G. Confined synthesis of MoS_2_ with rich co-doped edges for enhanced hydrogen evolution performance. J. Energy Chem. 2022, 70, 18–26. 10.1016/j.jechem.2022.01.001.

[ref59] Huang J.; Hao M.; Mao B.; Zheng L.; Zhu J.; Cao M. The Underlying Molecular Mechanism of Fence Engineering to Break the Activity-Stability Trade-Off in Catalysts for the Hydrogen Evolution Reaction. Angew. Chem., Int. Ed. Engl. 2022, 61 (10), e202114899 10.1002/anie.202114899. 34931747

[ref60] Lykhach Y.; Piccinin S.; Skála T.; Bertram M.; Tsud N.; Brummel O.; Camellone M. F.; Beranová K.; Neitzel A.; Fabris S.; Prince K. C.; et al. Quantitative Analysis of the Oxidation State of Cobalt Oxides by Resonant Photoemission Spectroscopy. J. Phys. Chem. Lett. 2019, 10 (20), 6129–6136. 10.1021/acs.jpclett.9b02398. 31553619

[ref61] Chen H.; Falling L. J.; Kersell H.; Yan G.; Zhao X.; Oliver-Meseguer J.; Jaugstetter M.; Nemsak S.; Hunt A.; Waluyo I.; et al. Nat. Commun. 2023, 14, 6689 10.1038/s41467-023-42301-7. 37898599 PMC10613203

[ref62] Song W.; Nie T.; Lai W.; Yang W.; Jiang X. Tailoring the morphology of Co-doped MoS_2_ for enhanced hydrodeoxygenation performance of *p-*cresol. CrystEngComm 2018, 20, 4069–4074. 10.1039/C8CE00510A.

[ref63] Li J.; Peng Y.; Qian X.; Lin J. Few-layer Co-doped MoS_2_ nanosheets with rich active sites as an efficient cocatalyst for photocatalytic H2 production over CdS. Appl. Surf. Sci. 2018, 452, 437–442. 10.1016/j.apsusc.2018.05.021.

[ref64] Song W.; Zhou S.; Hu S.; Lai W.; Lian Y.; Wang J.; Yang W.; Wang M.; Wang P.; Jiang X. Surface Engineering of CoMoS Nanosulfide for Hydrodeoxygenation of Lignin-Derived Phenols to Arenes. ACS Catal. 2019, 9 (1), 259–268. 10.1021/acscatal.8b03402.

[ref65] Cai L.; He J.; Liu Q.; Yao T.; Chen L.; Yan W.; Hu F.; Jiang Y.; Zhao Y.; Hu T.; et al. Vacancy-Induced Ferromagnetism of MoS_2_ Nanosheets. J. Am. Chem. Soc. 2015, 137 (7), 2622–2627. 10.1021/ja5120908. 25641111

[ref66] Hong W.; Meza E.; Li C. W. Controlling the Co–S coordination environment in Co-doped WS_2_ nanosheets for electrochemical oxygen reduction. J. Mater. Chem. A 2021, 9 (35), 19865–19873. 10.1039/D1TA02468J.

[ref67] Chukwu E.; Molina L.; Rapp C.; Morales L.; Jin Z.; Karakalos S.; Wang H.; Lee S.; Zachman M. J.; Yang M. Crowded supported metal atoms on catalytically active supports may compromise intrinsic activity: A case study of dual-site Pt/α-MoC catalysts. Appl. Catal. B: Environ. 2023, 329, 122532 10.1016/j.apcatb.2023.122532.

