# Supplementary material for: Elucidating Local Structure and Positional Effect of Dopants in Colloidal Transition Metal Dichalcogenide Nanosheets for Catalytic Hydrogenolysis
Source: J Phys Chem C Nanomater Interfaces. 2024 Mar 6;128(11):4470–82. doi: 10.1021/acs.jpcc.3c07408 (PMC10961832; doi:10.1021/acs.jpcc.3c07408)
Supplement: Supplementary file 1 — jp3c07408_si_001.pdf [file jp3c07408_si_001.pdf]

Elucidating Local Structure and Positional Effect of Dopants in Colloidal Transition Metal Dichalcogenide Nanosheets for Catalytic Hydrogenolysis

Steven L. Farrell[1], Mersal Khwaja[1], Ingrid J. Paredes[1], Christopher Oyuela[1], William Clarke[1], Noah Osinski[1], Amani M. Ebrahim[2], Shlok J. Paul[1], Haripriya Kannan[1], Håvard Mølnås[1], Lu Ma[4], Steven N. Ehrlich[4], Xiangyu Liu[1], Elisa Riedo[1], Srinivas Rangarajan[3], Anatoly I. Frenkel[2,5]*, and Ayaskanta Sahu[1]**

[1]Department of Chemical and Biomolecular Engineering, New York University, Brooklyn, New York 11201, USA.

[2]Department of Materials Science and Chemical Engineering, Stony Brook University, Stony Brook, New York 11794, USA.

[3]Department of Chemical and Biomolecular Engineering, Lehigh University, Bethlehem, PA 18015, USA.

[4]National Synchrotron Light Source II, Brookhaven National Laboratory, Upton, New York 11973, USA.

[5]Chemistry Division, Brookhaven National Laboratory, Upton, New York 11973, USA.

**Corresponding authors: srr516@lehigh.edu; anatoly.frenkel@stonybrook.edu; asahu@nyu.edu*

Supporting Information (SI)

Characterization Methods

X-ray Diffraction (XRD)

Samples were characterized with XRD using a Bruker AXS D8 Discover GADDS Microdiffractometer at New York University's Shared Instrument Facility. All samples prepared by drop-casting from cyclohexane and drying on clean 1-cm^2 glass slides, and were measured with a Cu Kα source.

Fourier Transform Infrared Spectroscopy (FT-IR)

FT-IR was performed using a Thermo Scientific Nicolet 6700 FT-IR. Samples were prepared by drop-casting from cyclohexane and drying on clean CaF_2 crystals.

Atomic Force Microscopy (AFM)

AFM measurements were performed using a Bruker MultiMode 8 AFM operating in tapping mode. Flattening and z- scale adjustment of AFM images was performed with Gwyddion software.[1]

Thermogravimetric Analysis (TGA)

TGA was performed using a TA instruments TGA550. The sample was heated in ultrapure nitrogen from 23°C to 100°C at 10°C/min followed by a 15-minute isothermal hold. The sample was then ramped to 400°C at 20°C/min and held at this temperature for 70 minutes.

High Resolution Transmission Electron Microscopy (HRTEM)

HRTEM was performed using a 200 kV FEI Titan Themis Scanning TEM. Samples were prepared by drop-casting dilute samples of nanosheets in cyclohexane (fresh samples) or dimethylformamide (post-HDS) onto carbon coated copper grids and drying at 80°C.

Scanning Electron Microscopy-Energy Dispersive X-ray Spectroscopy (SEM-EDX)

SEM-EDX was performed using a Zeiss Gemini Ultra-55 Analytical Field Emission Scanning Electron Microscope. Samples were prepared by drop-casting on 1 cm^2 silicon substrates. EDX was performed targeting Co, Mo, and S compositions.

X-ray Photoelectron Spectroscopy (XPS)

XPS for examining Co, Mo, and S was performed using a Physical Electronics Versaprobe II XPS. Spectra were collected using an Al Kα source set to 49.4 W and 14.87 keV (250 meV resolution) with a 200 μm beam diameter. The survey pass energy was set to 117.40 eV, while the elemental pass energies were set to 29.35 eV. Samples were prepared by drop-casting nanosheet suspensions onto glass substrates and drying at 70°C for 10 min. Prior to analysis, spectra were corrected by shifting the C1s peak to 284.8 eV.

X-ray Absorption Spectroscopy (XAS)

Samples were measured at Brookhaven National Laboratory using the National Synchrotron Light Source-II (NSLS-II) facility's Quick X-ray Absorption and Scattering (QAS, 7-BM) and Tender Energy X-ray Absorption Spectroscopy (TES, 8-BM) beamlines. For hard X-ray energy measurements at QAS, samples were smeared onto clear adhesive tape, folded up to 8

times, and measured at the Co and Mo K-edges in fluorescence and transmission modes, respectively. At the TES beamline, the samples were smeared onto single-layer, non-adhesive 1-cm^2 Kapton films and measured at the S *K*-edge. The collected XAS data were then analyzed using the Demeter software package; XANES and EXAFS were analyzed in ATHENA, while modeling of the EXAFS data for Co and Mo K-edges was performed in ARTEMIS.[2] Parameters used in the fits include an amplitude factor of 0.76 (determined by fitting Co foil) and a *k*-weighting of 2. The R-range was 1.0-2.3 Å, and the *k*-range was 2.0-12.0 $Å^{-1}$ (dk = 2). At the Co edge, Co-S and Co-O were modeled iteratively and checked for stability. Wavelet transforms were performed using a modification of the code originally published by Muñoz et al.[3] Cauchy order was set to 200, and the R-space distance was set from 0.2 to 6.0 Å (no. of intervals = 200).

Gas Chromatography-Mass Spectroscopy (GC-MS)

Samples taken from the reaction mixture were analyzed using a GC-2030 gas chromatograph and GCMS-QP2020 NX gas chromatograph-mass spectrometer from Shimadzu. A 0.2-μL volume of analyte was injected at a column temperature of 40°C using a Shimadzu AOC-20i autosampler, then after one minute the column temperature increased to 250°C over 10 min. Prior to analysis, a calibration curve of the area ratio between thiophene and the n-decane reference was prepared using solutions of known concentrations. The concentrations of thiophene in the mixture before and after reaction were measured using the area under the identified peaks in the chromatograph, and the percent conversion of thiophene was calculated for each reaction run as follows:

$$X_{Thiophene} = \frac{C_{Thiophene,Before} - C_{Thiophene,After}}{C_{Thiophene,Before}}$$

GC-MS measurements were taken three times to establish an average value.

Turnover Frequency Computation

Per our geometric structure computation (Table S4 in the SI), we estimate an average of 36 edge site atoms for truncated triangular sheets. Combining this with our DFT and XAS observations of, and assuming decorated Co cannot sit next to another decorated Co (per XAS results and steric considerations), we compute that a Co:Mo ratio of 16.2% is the saturation point for the nanosheet edges. Assuming that Co covers an Mo site on the edge but creates a new site on the basal plane, we therefore can compute the number of sites per nanosheet, as used in Figure 1f, as follows:

If Co:Mo ≤ 16.2%: $N_{sites} = N_{edge\ Mo}$,

If Co:Mo > 16.2%: $N_{sites} = N_{edge\ Mo} + N_{total\ Mo} * (X_{Co:Mo} - 0.162)$

The quantity $N_{edge\ Mo}$ refers to the number of Mo atoms along a single nanosheet's perimeter. $N_{total\ Mo}$ refers to the total number of Mo atoms present in a single nanosheet. This is based on the assumption that a Co atom on the edge blocks one Mo atom, thus the number of edge sites does not change. However, any Co in excess of the saturation point (16.2%) would likely adsorb to the inert basal plane, meaning it creates an additional site.

The turnover frequency per unit Co in Figure 1g of the main text is calculated as follows:

$$TOF = \frac{N_{Thio_{CoMoS_2}} - N_{Thio_{MoS_2}}}{N_{Co} * t}$$

$N_{Thio_{CoMoS_2}}$ is the number of moles of thiophene converted using a Co-doped MoS_2 catalyst. $N_{Thio_{MoS_2}}$ is the number of moles of thiophene converted using nanoscale MoS_2 (0% Co:Mo). N_{Co} is the number of moles of Co present in the Co-doped MoS_2 catalyst. t is the time of the reaction, in hours.

Ligand Removal Procedure on Nanoscale MoS_2

The procedure for the removal of ligands from our nanosheets is adapted from the work of Meeree Kim et al.[4] As the ligand-removed particles were only used for measurement in X-ray photoelectron spectroscopy (XPS), small quantities were used.

In a typical ligand removal sequence, 20 mg of as-synthesized MoS_2 nanosheets are added to a vial inside a nitrogen-filled glovebox, along with 40 mg of nitrosyl tetrafluoroborate ($NOBF_4$), 4 mL of chloroform, and a stir bar. The vial is sealed, sonicated for 10 min, then placed on a stir plate and allowed to stir at 700 RPM for 60 min at ambient temperature.

Inside the glovebox, the ligand-free particles are cleaned twice by addition of 10 mL of hexane and 10 mL toluene and centrifuging at 9500 RPM for 10 min to remove any remaining long-chain organic ligands and excess chloroform. The particles are then cleaned a further two times by the addition of 5 mL dimethylformamide (DMF) and 15 mL toluene to remove excess $NOBF_4$, again centrifuging as above. The particles are then dried under vacuum overnight. After the ligand removal, particles no longer dispersed in hexane but easily dispersed in DMF.

X-ray Diffraction

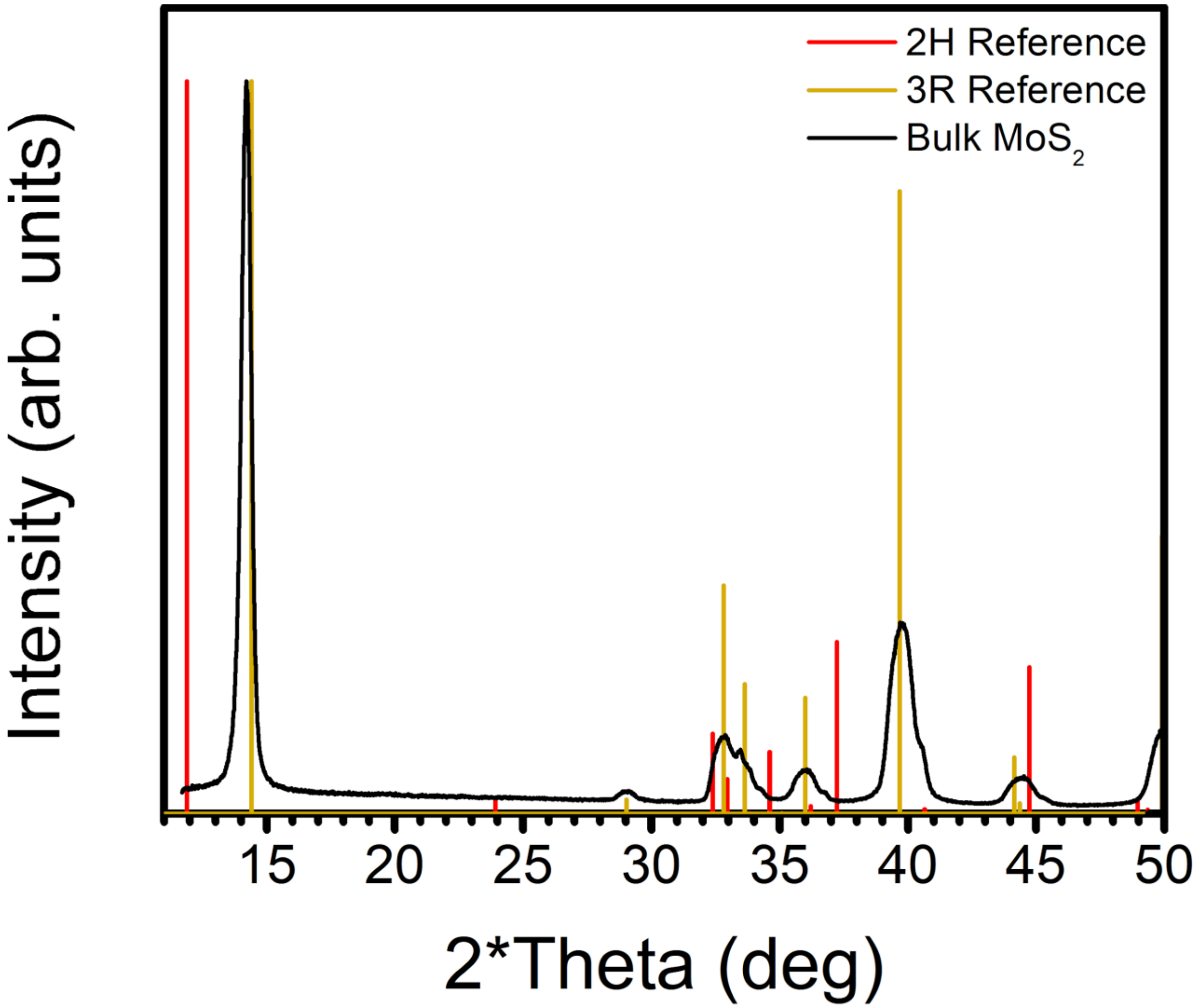

Figure S1. X-ray diffraction (XRD) pattern of bulk MoS_2, which matches more closely to the 3R reference pattern than 2H. Both 3R and 2H display hexagonal arrangement within each layer, but differ in interlayer alignment. Reference peaks are from Materials Project.[5]

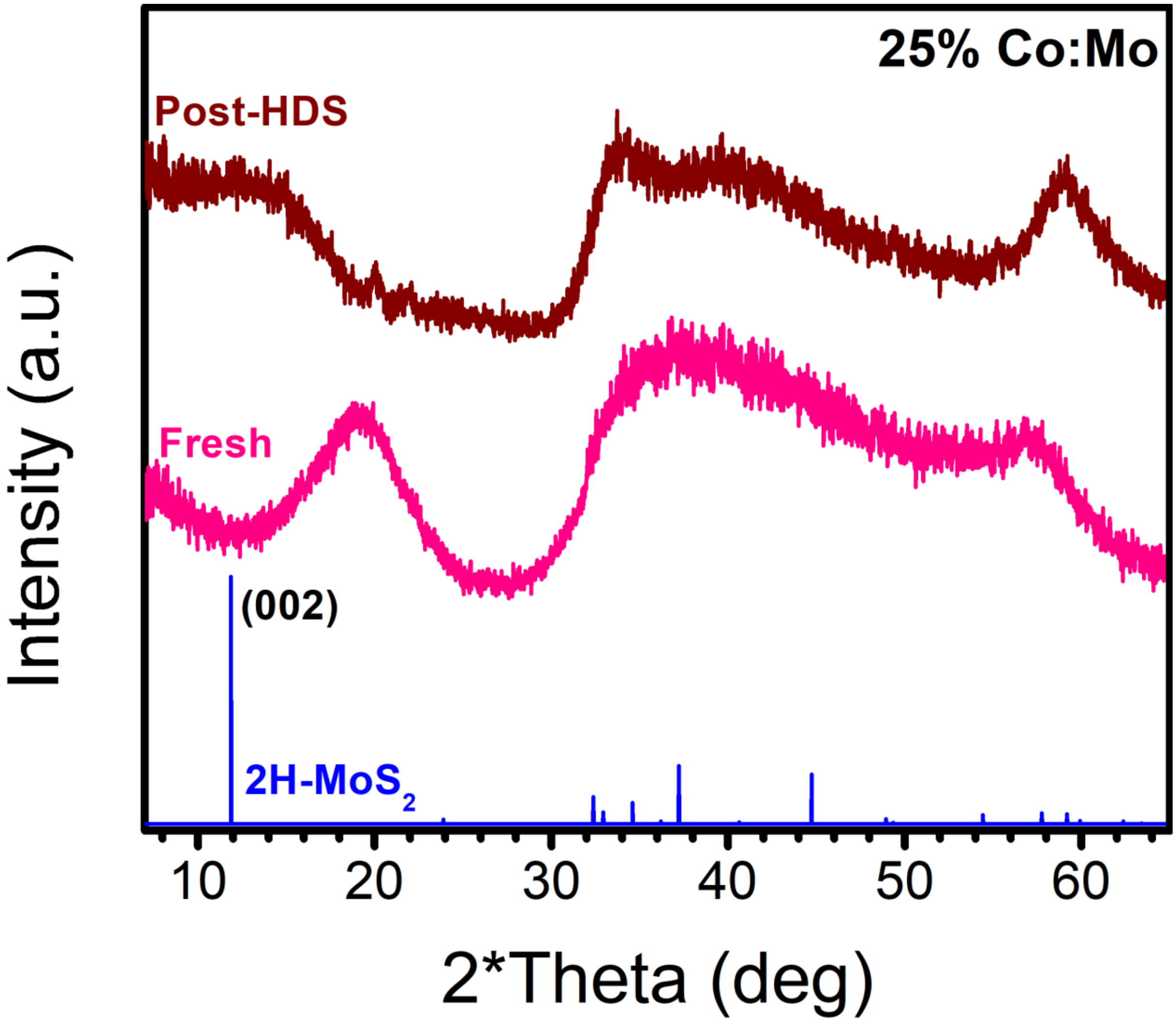

Figure S2. X-ray diffraction patterns of nanoscale MoS_2 (0% Co:Mo), before and after use in HDS. Here we note the slight change in structure, particularly the appearance of peaks at 33 and 40 degrees, indicative of a structural change. The slight appearance of the (002) peak in the post-HDS pattern correlates to the small amount of stacking observed in HRTEM. Reference peaks are from Materials Project.[5]

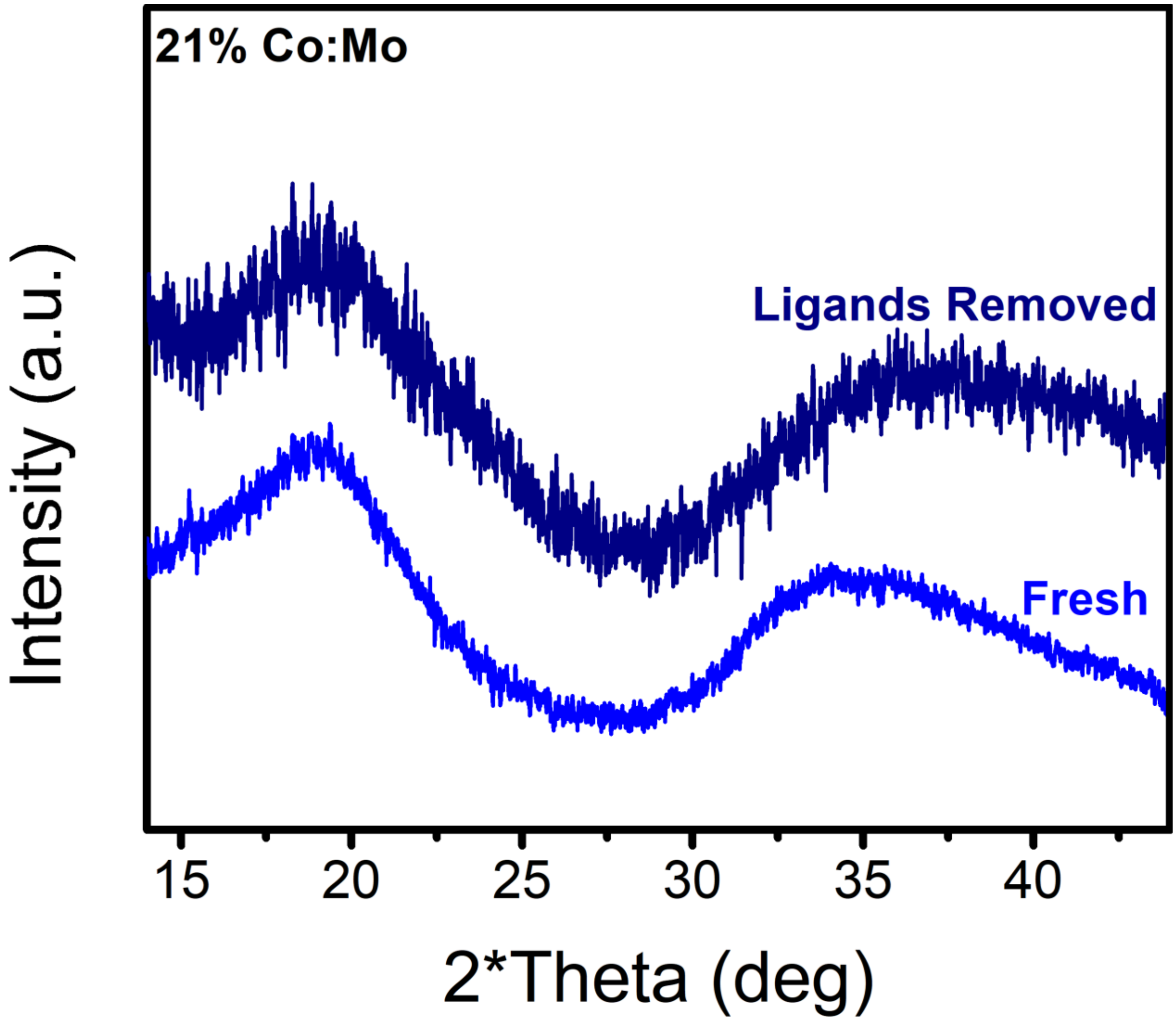

Figure S3. X-ray diffraction patterns of Co-MoS_2 (21% Co:Mo), before and after ligand removal. We do not observe any reasonable change to the structure to indicate a loss of the distorted 1T phase or which might imply a change in the expected XPS results.

Energy Dispersive X-ray Spectroscopy

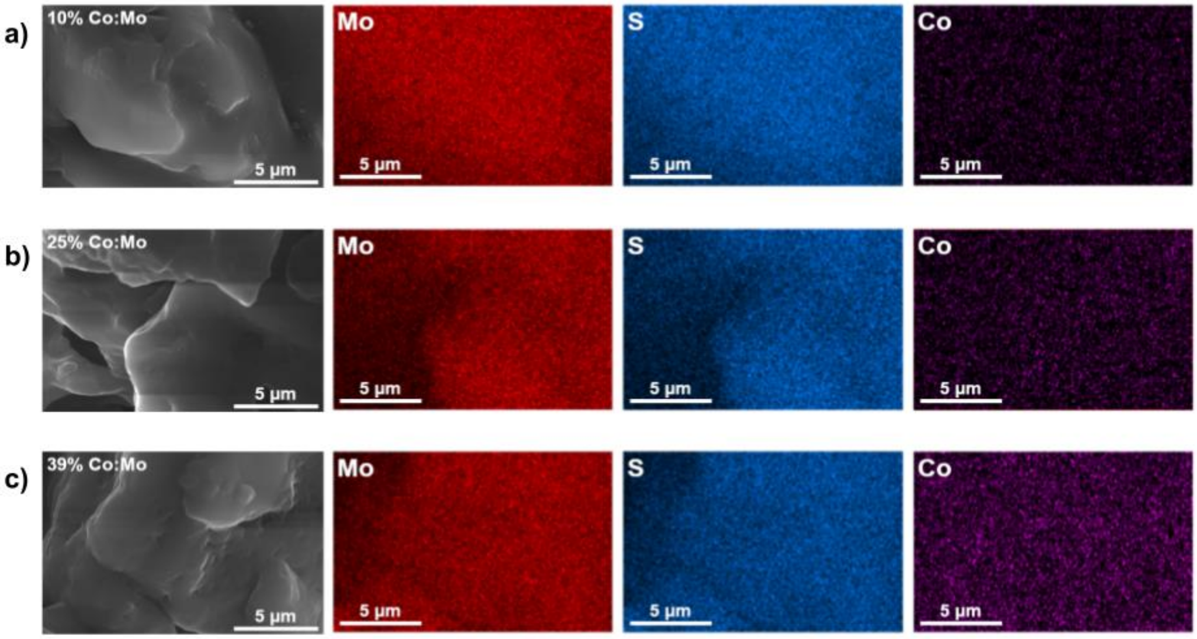

Figure S4. Scanning electron microscopy and energy dispersive x-ray spectroscopy (SEM-EDX) of fresh a) 10% Co:Mo, b) 25% Co:Mo, and c) 39% Co:Mo samples. Signals were collected at Lα (Mo) and Kα (Co, S) edges. Co shows an increase in signal intensity as Co concentration increases and is well-dispersed.

Atomic Force Microscopy

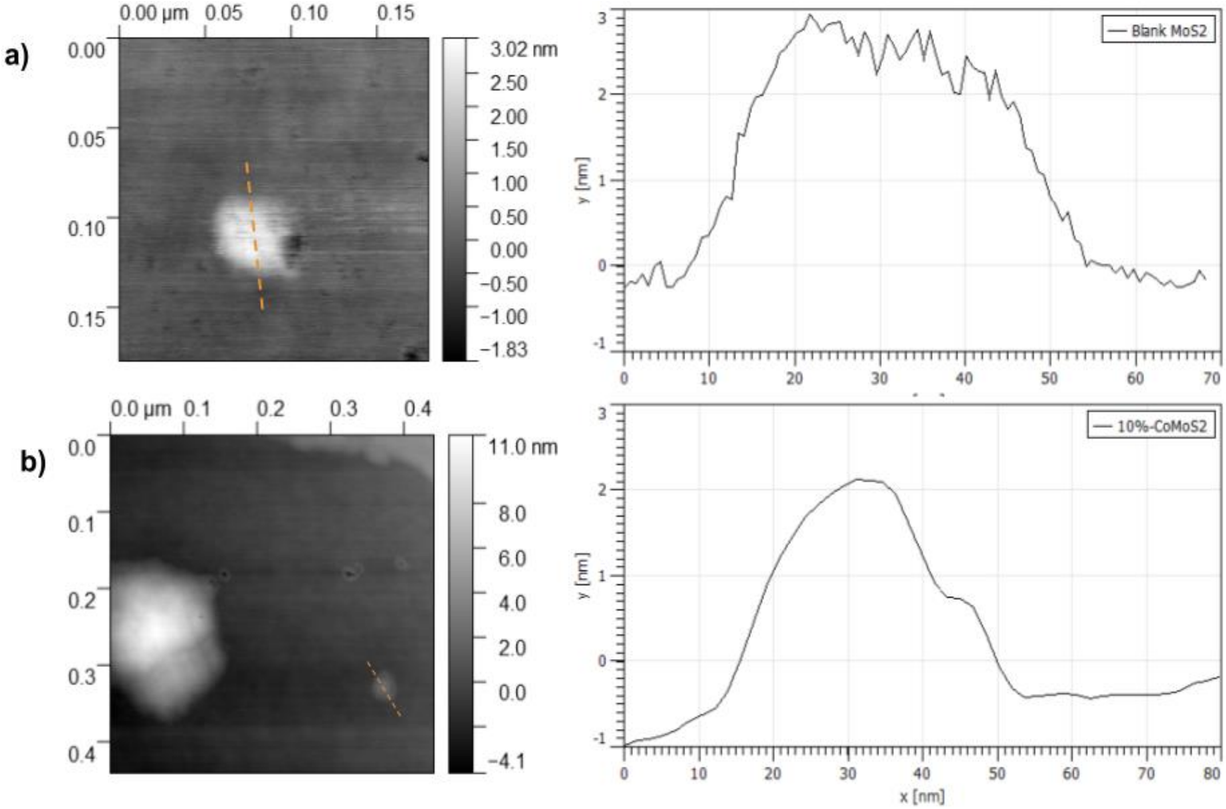

Figure S5. Atomic force microscopy (AFM) topography imaging of a) 0% and b) 10% Co:Mo nanosheets, showing 2-3 nm thicknesses or roughly 3-4 layers of MoS_2. The line scans for the corresponding profiles are indicated by the dotted orange line.

Thermogravimetric Analysis

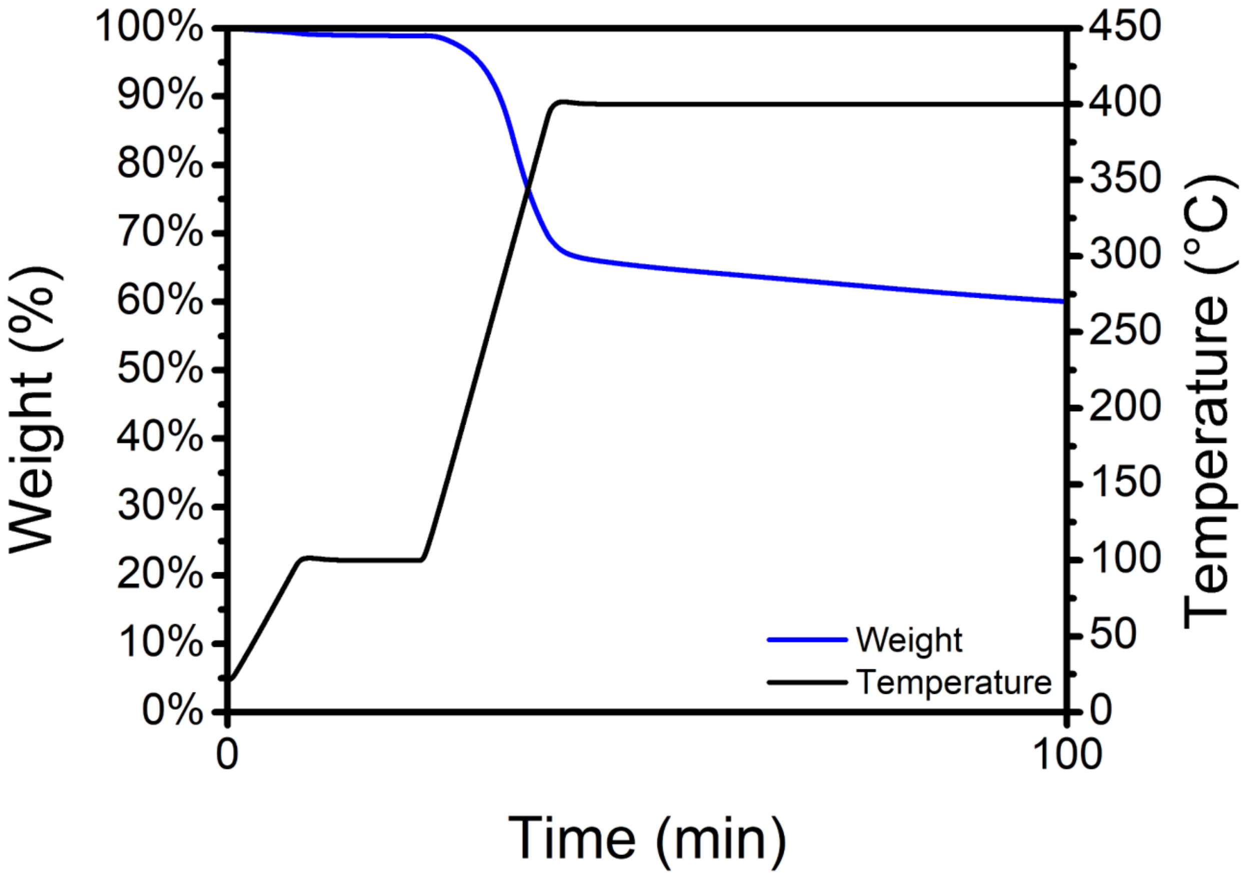

Figure S6. Thermogravimetric profile of fresh 21% Co:Mo sample. The oleylamine and oleic ligands evaporate at temperatures above 360°C. 67% of mass remained after the ligand evaporation step, yielding a ligand weight of 33% by mass.

Reaction Scheme in Hydrodesulfurization

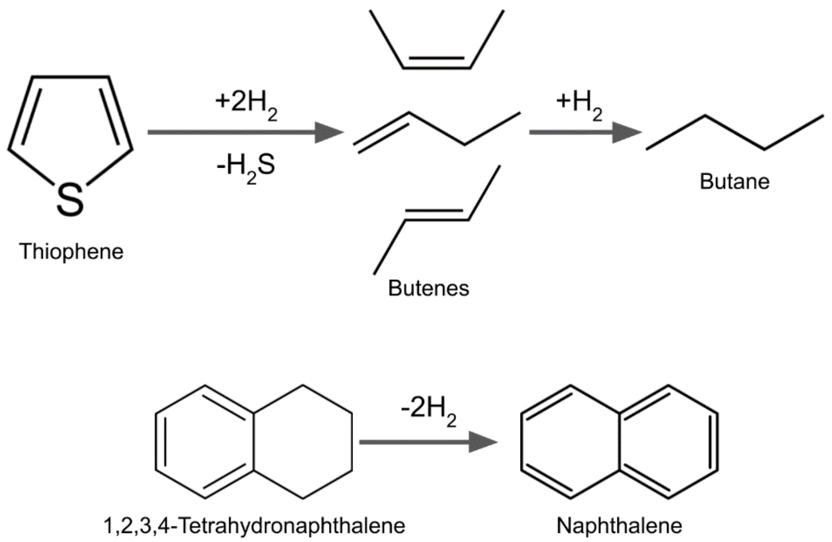

Figure S7. Expected reaction scheme under HDS conditions. Thiophene is expected to decompose into butenes, which may be further hydrogenated into butanes. 1,2,3,4-Tetrahydronapthalene (Tetralin) acts as both a solvent for the reaction and a hydrogen donor, as it forms naphthalene during the reaction. Decomposition of thiophene also produces hydrogen sulfide.

Bulk MoS_2 in Hydrodesulfurization

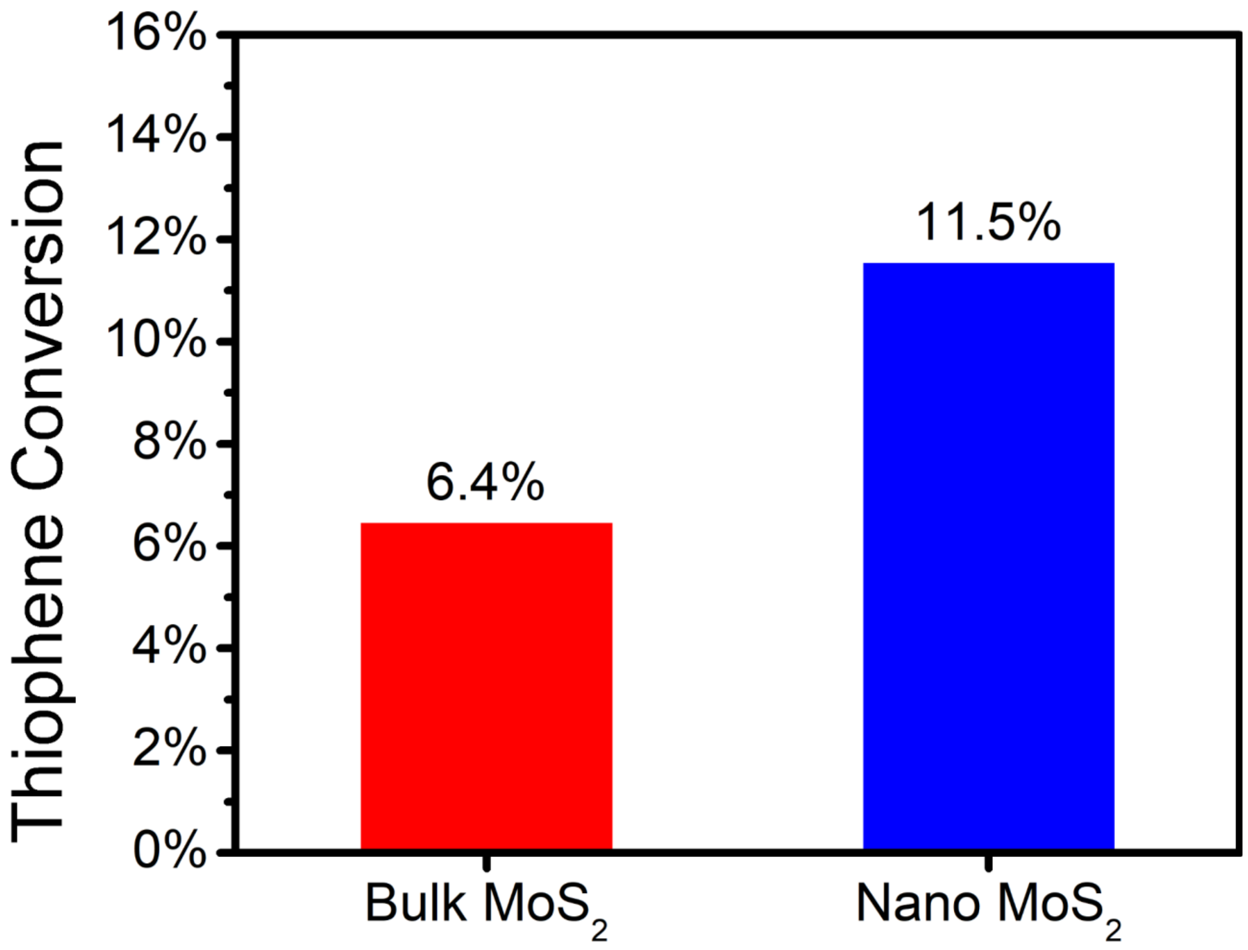

Figure S8. Thiophene conversions of bulk and nanoscale (0% Co:Mo) MoS_2 after three hours HDS. The increase in activity at the nanoscale is likely due to the decreased sheet size, which increases the number of active edge sites.

Catalyst Stability Testing

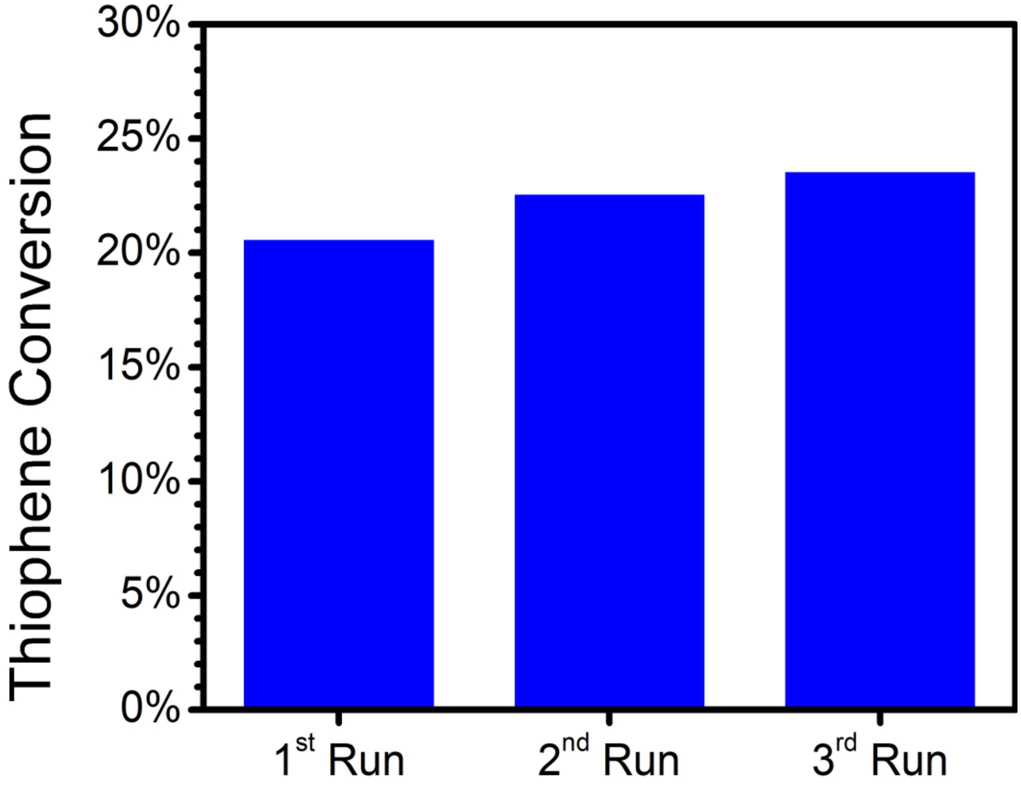

Figure S9. Reuse of 21% Co:Mo catalyst in HDS across three runs. The increase in activity between 1st and 2nd run is likely due to the partial loss of ligands (or presence of Co-on-basal prefixed rather than Co-Oleate during heat-up step).

Time-Resolved XAS

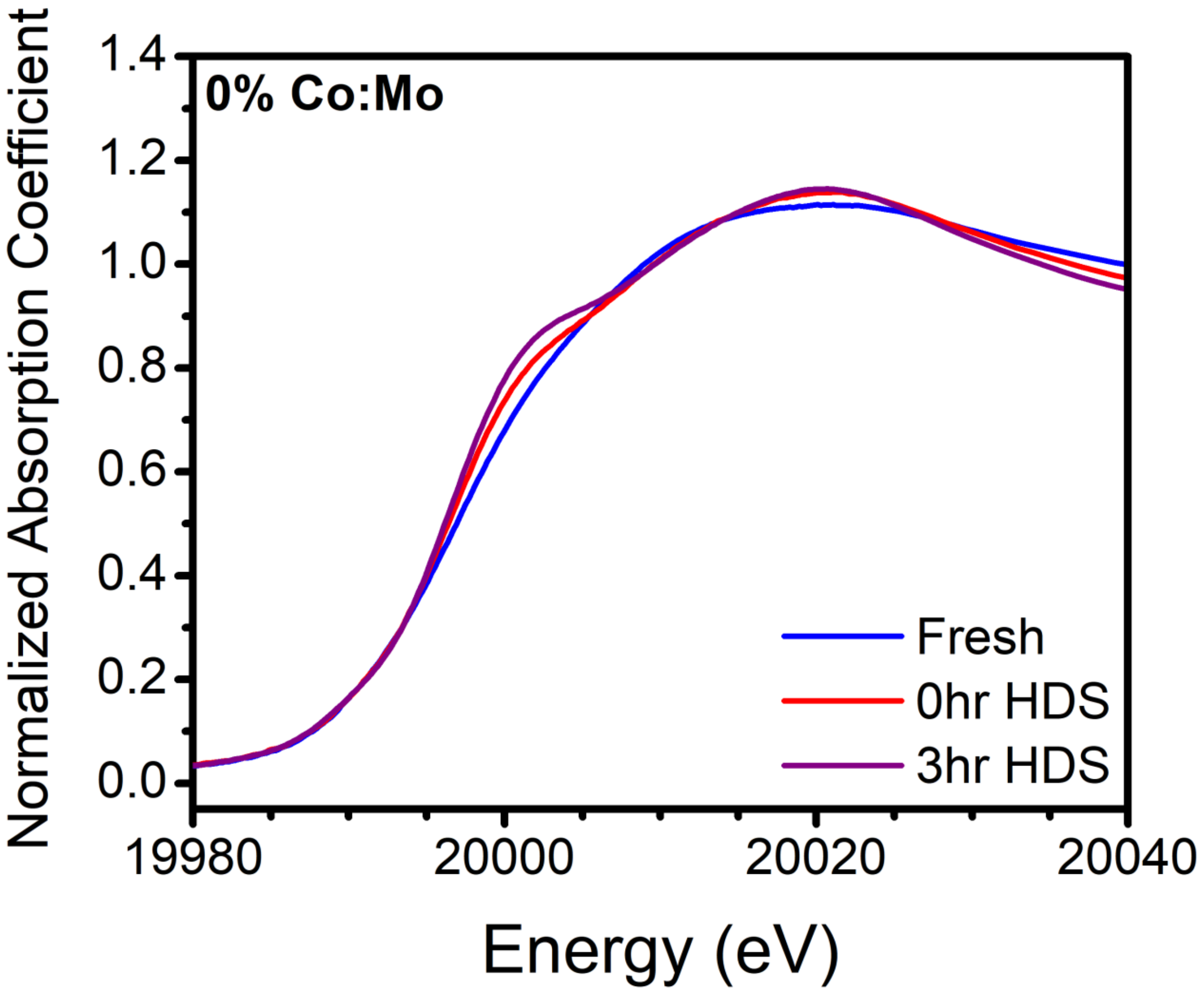

Figure S10. Ex situ Mo K-edge XANES of 0% Co:Mo samples fresh, after heating to 300°C in reaction conditions (0hr HDS), and after three hours use in HDS (3hr HDS). The “0hr HDS” sample denotes catalyst that was pressurized and heated to reactor conditions (150 PSIG, 300°C) and then cooled after reaching temperature.

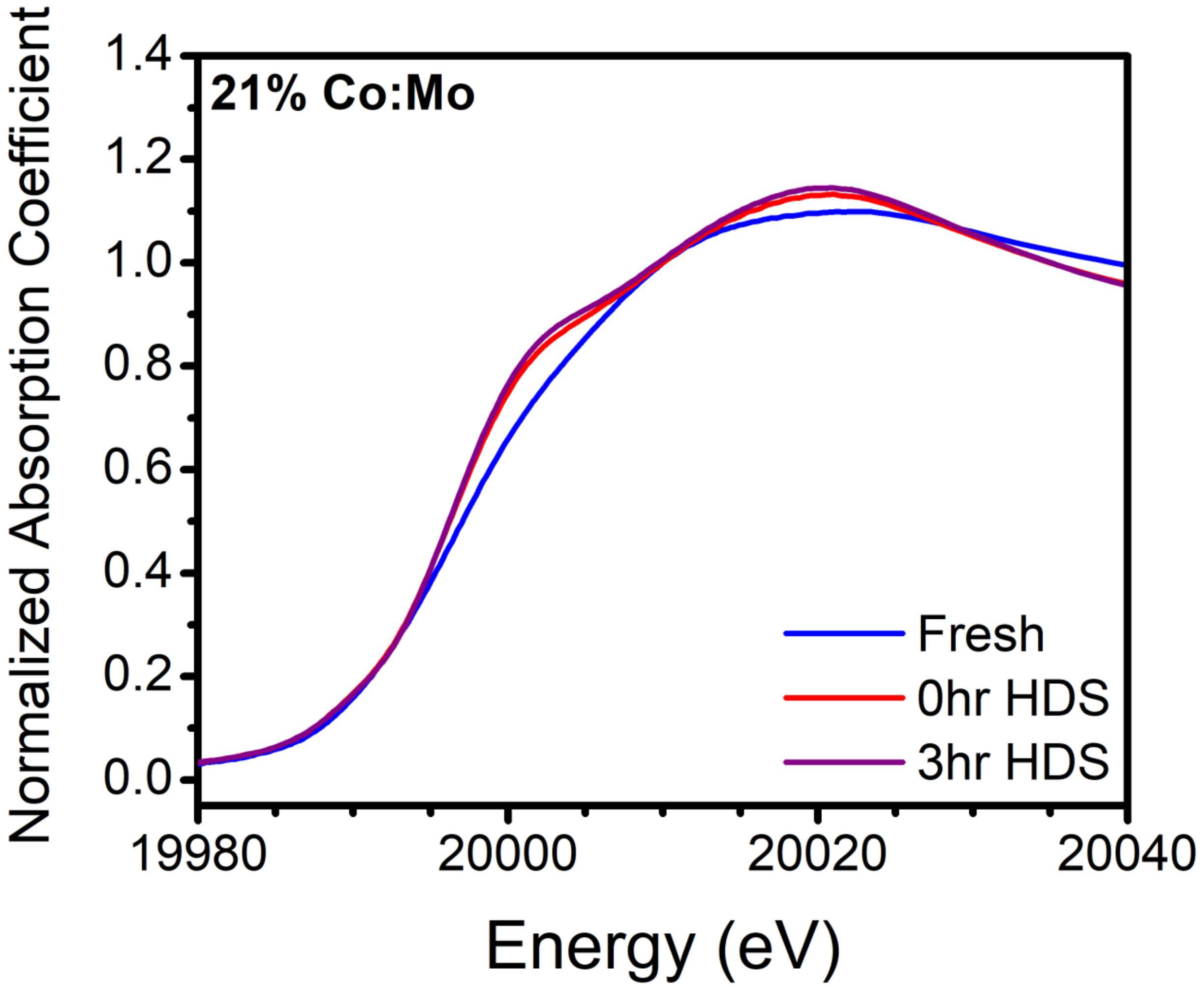

Figure S11. Ex situ Mo K-edge XANES of 21% Co:Mo samples fresh, after heating to 300°C in reaction conditions (0hr HDS), and after three hours use in HDS (3hr HDS). As observed in the 0% Co:Mo sample, the phase change occurs during the heatup to 300°C and as such the active catalyst is in the 2H phase for the duration of the reaction.

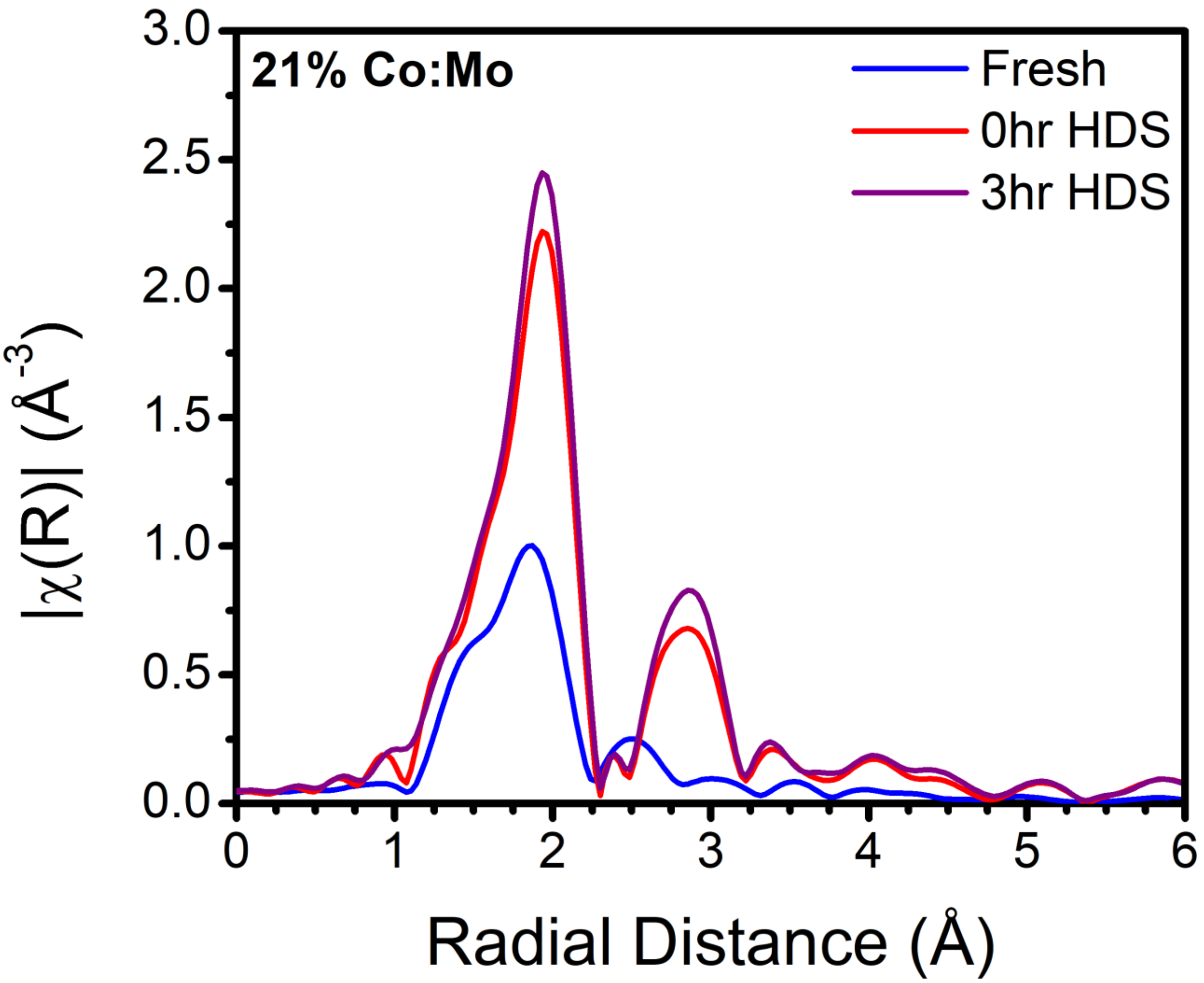

Figure S12. Ex situ Mo K-edge EXAFS of 21% Co:Mo samples fresh, after heating to 300°C in reaction conditions (0hr HDS), and after three hours use in HDS (3hr HDS). The phase change occurs during the heat up to 300°C and as such the active catalyst is in the 2H phase for the duration of the reaction.

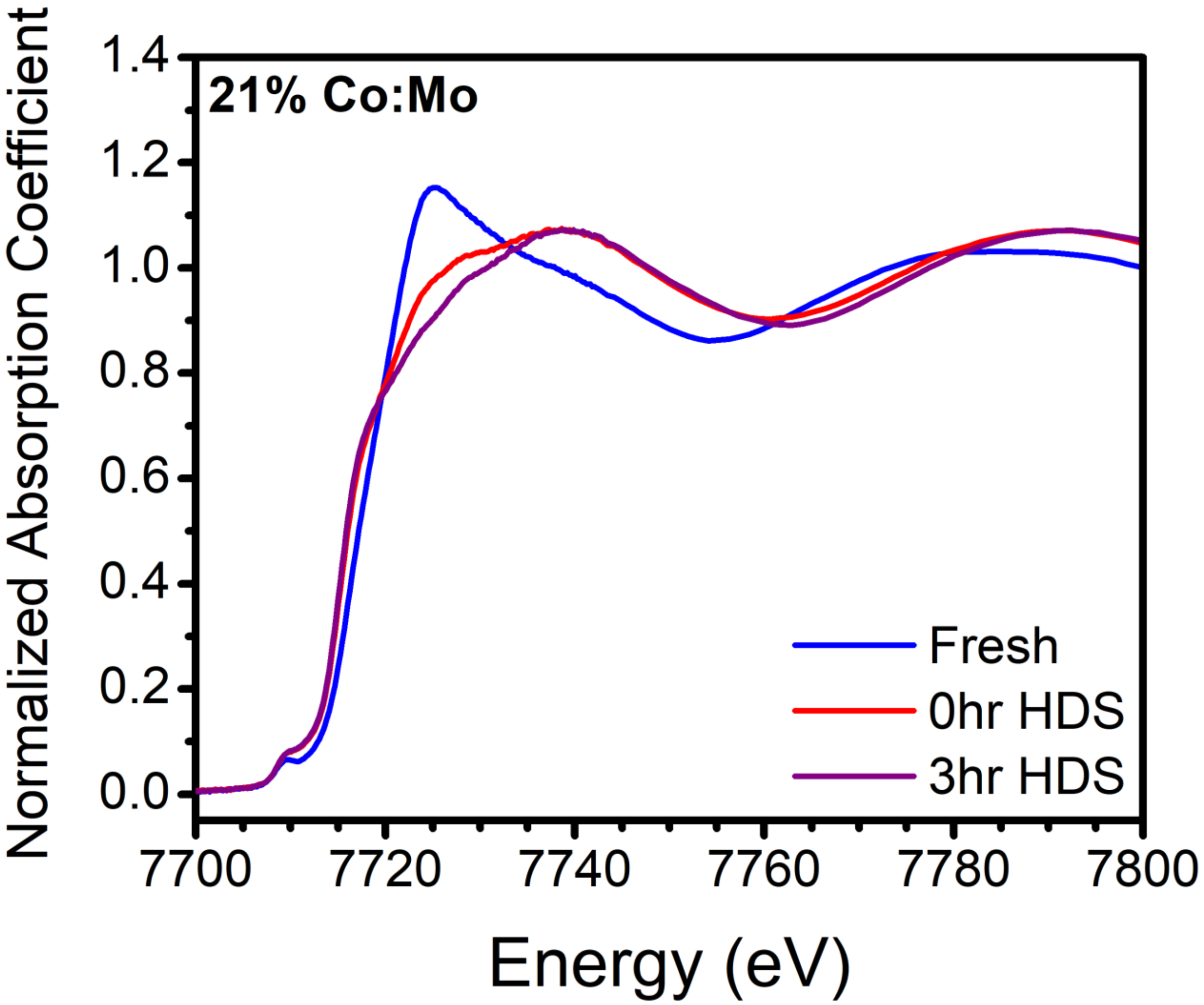

Figure S13. Ex situ Co K-edge XANES of 21% Co:Mo samples fresh, after heating to 300°C in reaction conditions (0hr HDS), and after three hours use in HDS (3hr HDS). The cobalt state changes dramatically before (fresh) and after (0hr HDS) heatup to 300°C. This is attributed to the affixing of Co atoms from Co-Oleate to the basal plane and the removal of excess oleate ligands.

EXAFS Modeling

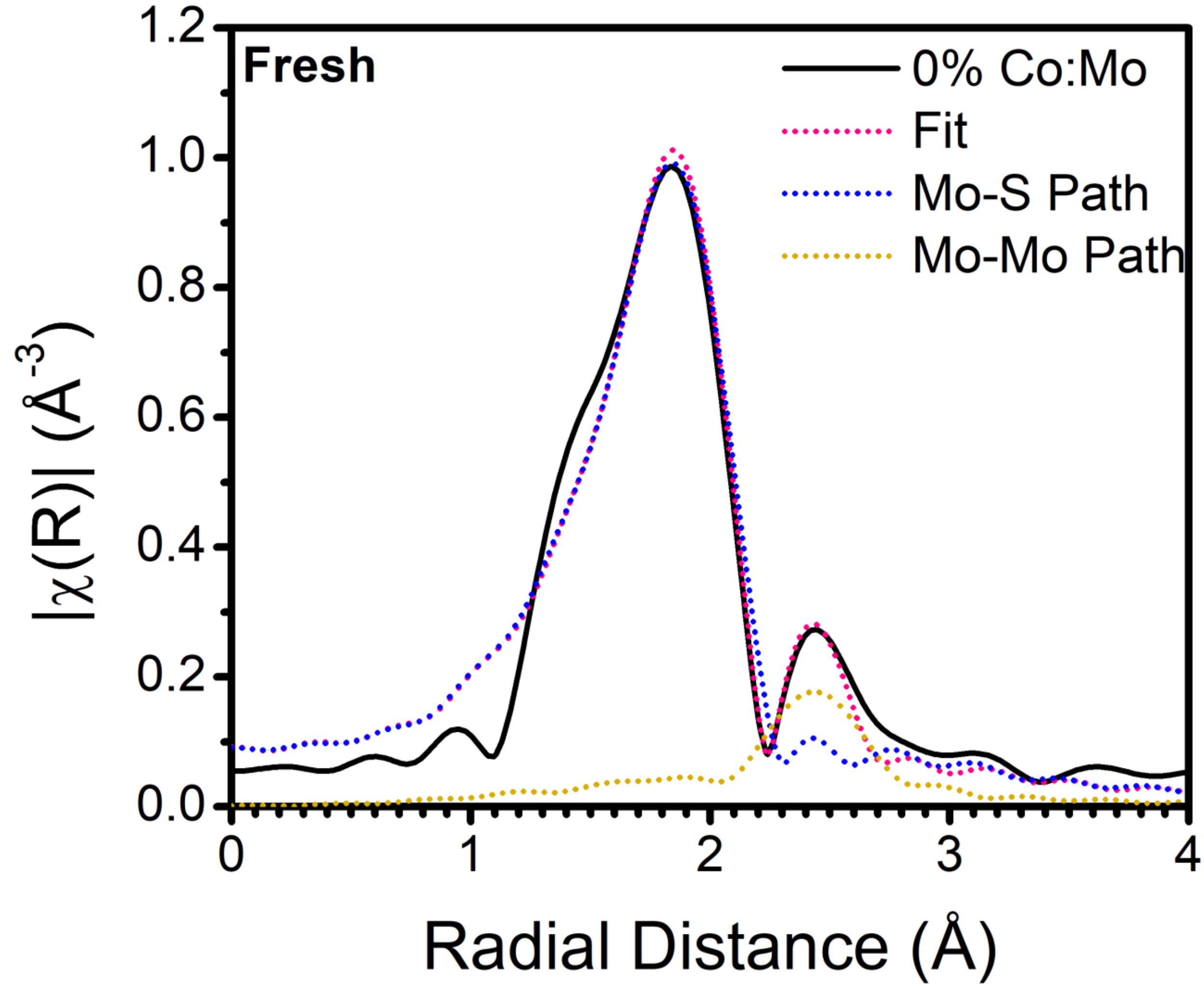

Figure S14. Fitting of fresh, as-synthesized nanoscale MoS_2 Mo K-edge EXAFS in Artemis, showing the truncated Mo-Mo path contribution and fit to the 1T'-MoS_2 structure, as observed in literature.[6,7]

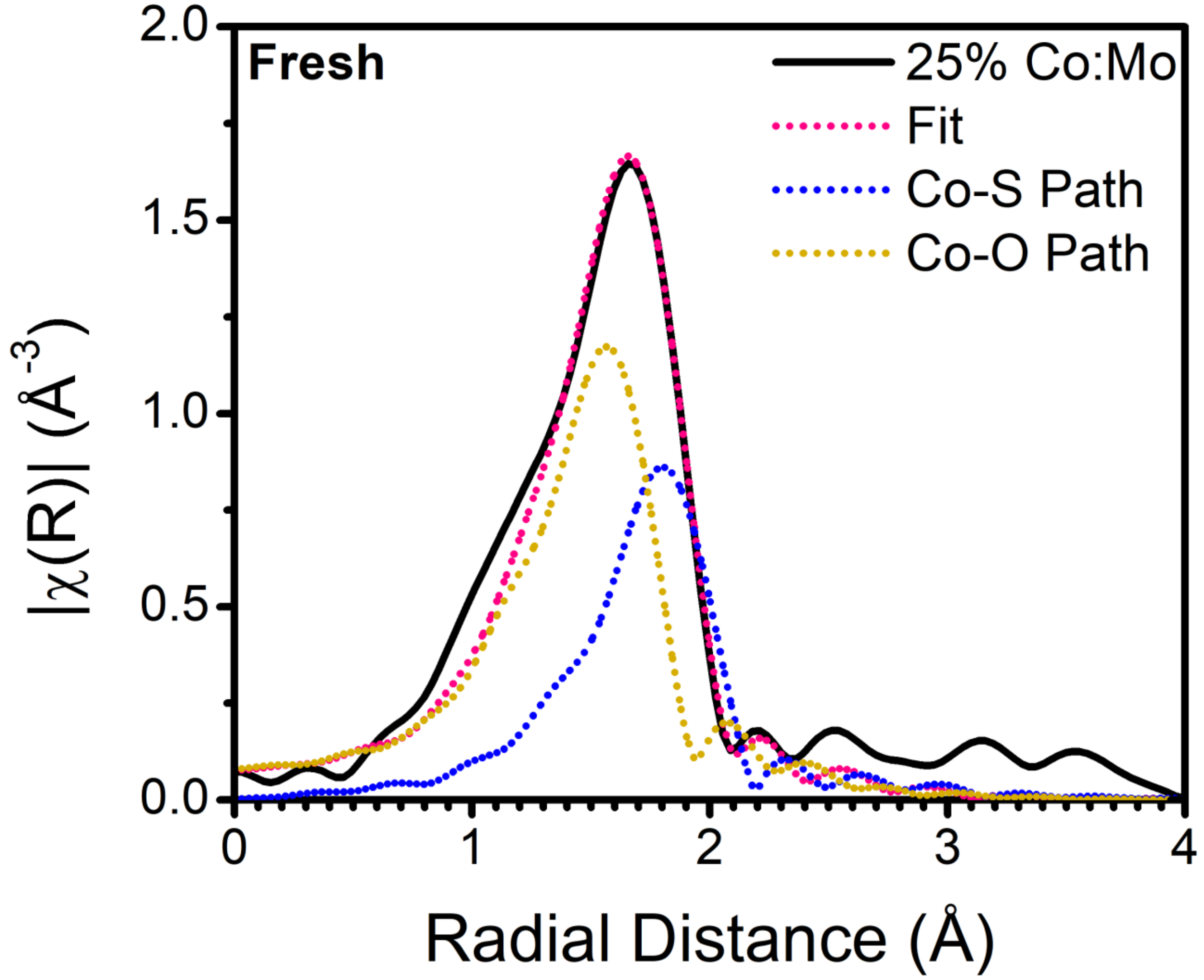

Figure S15. Co K-edge Artemis fitting of 25% Co:Mo fresh catalyst EXAFS showing both Co-S and Co-O pathways.

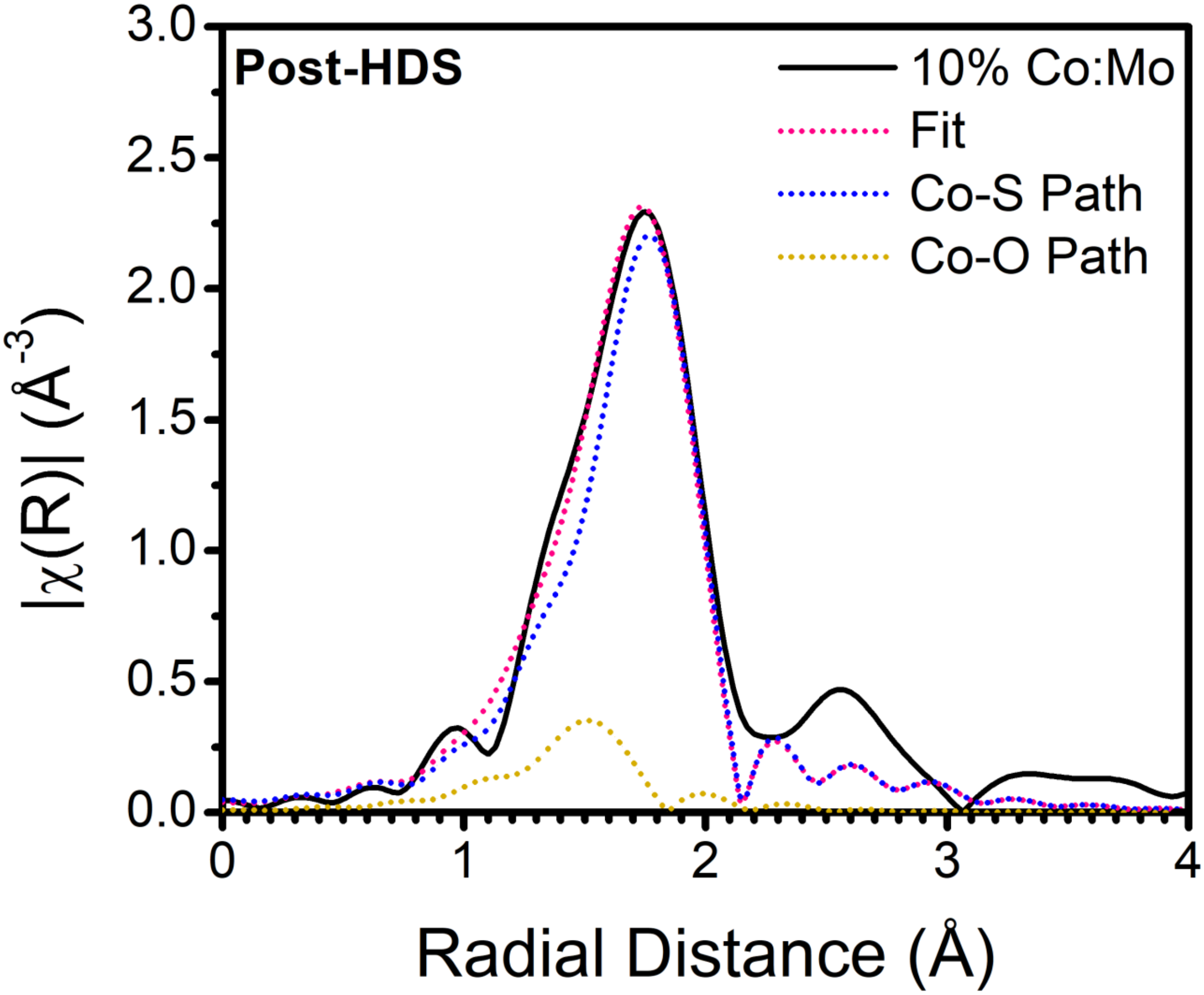

Figure S16. Co K-edge Artemis fitting of 10% Co:Mo post-HDS catalyst EXAFS showing both Co-S and Co-O pathways.

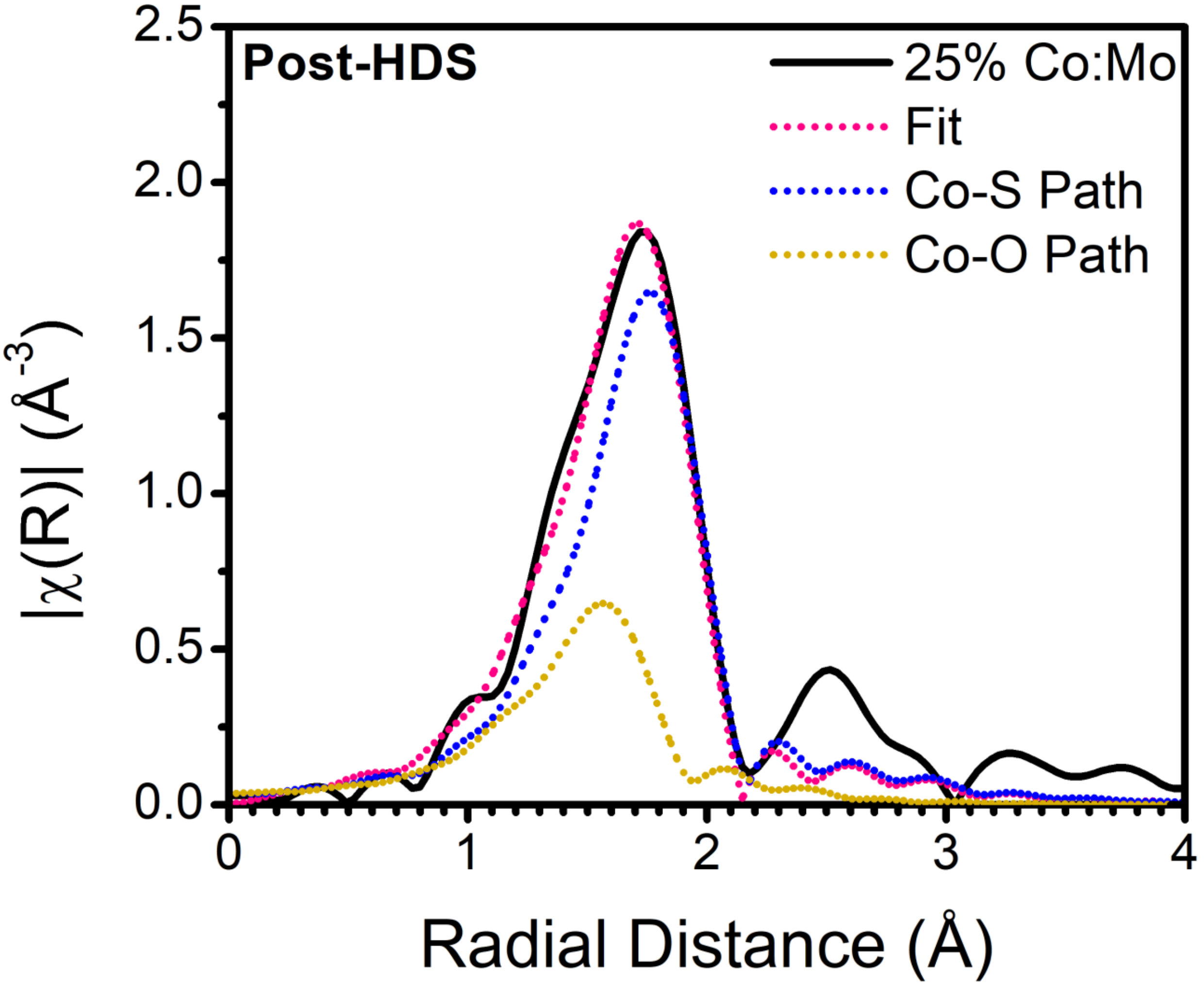

Figure S17. Co K-edge Artemis fitting of 21% Co:Mo post-HDS catalyst EXAFS showing both Co-S and Co-O pathways.

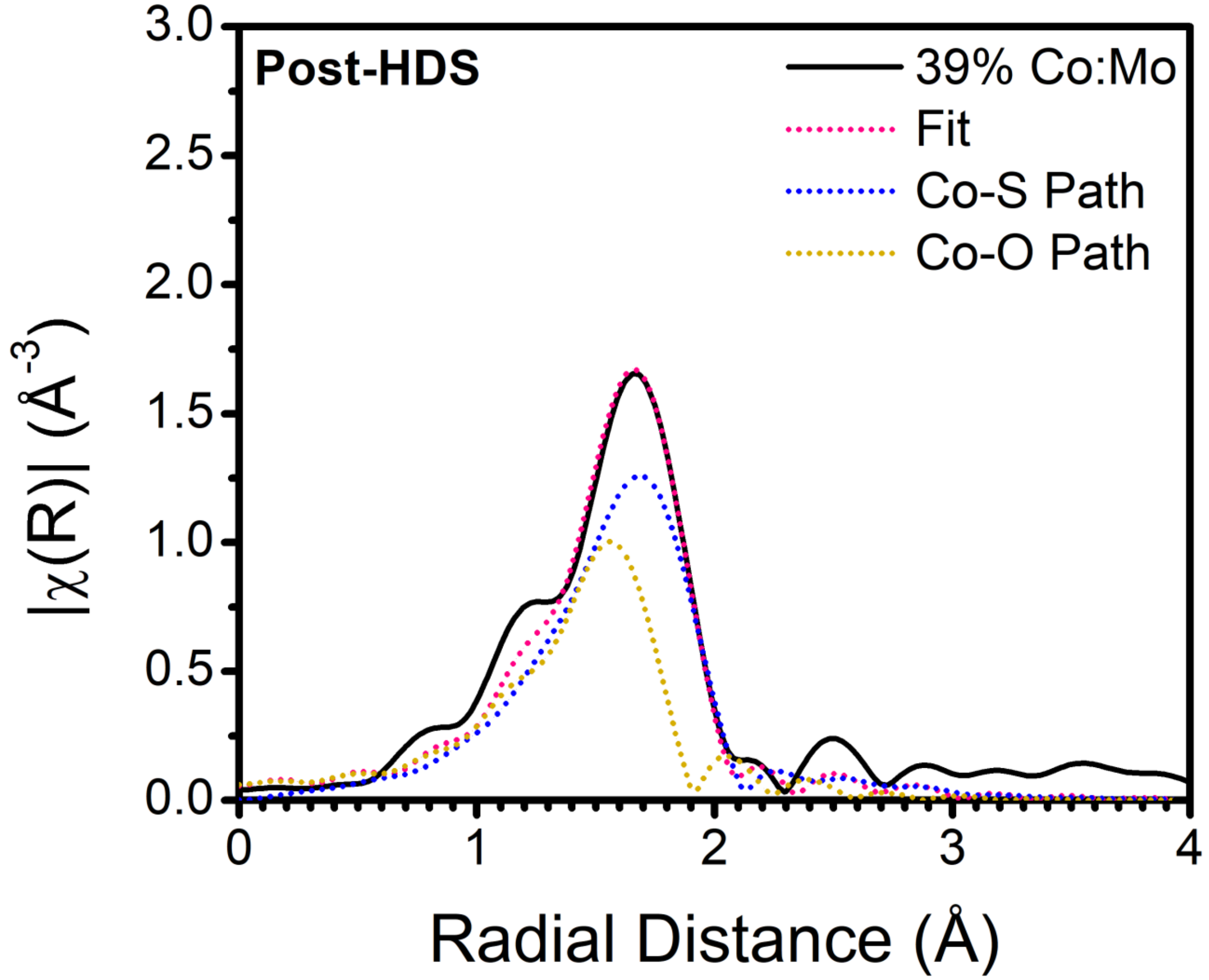

Figure S18. Co K-edge Artemis fitting of 39% Co:Mo post-HDS catalyst EXAFS showing both Co-S and Co-O pathways.

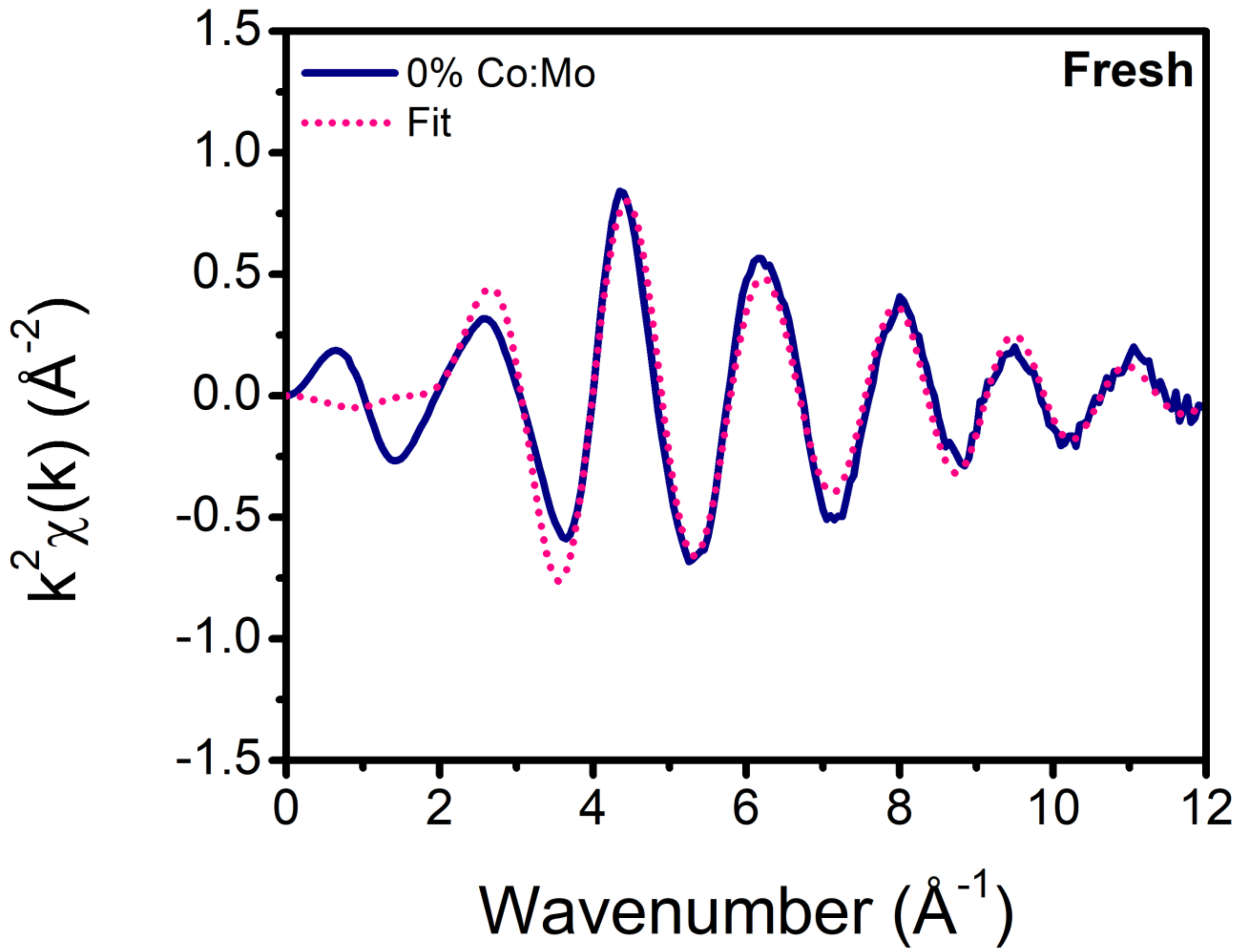

Figure S19. Artemis fitting in k-space of the Mo K-edge on nanoscale MoS_2.

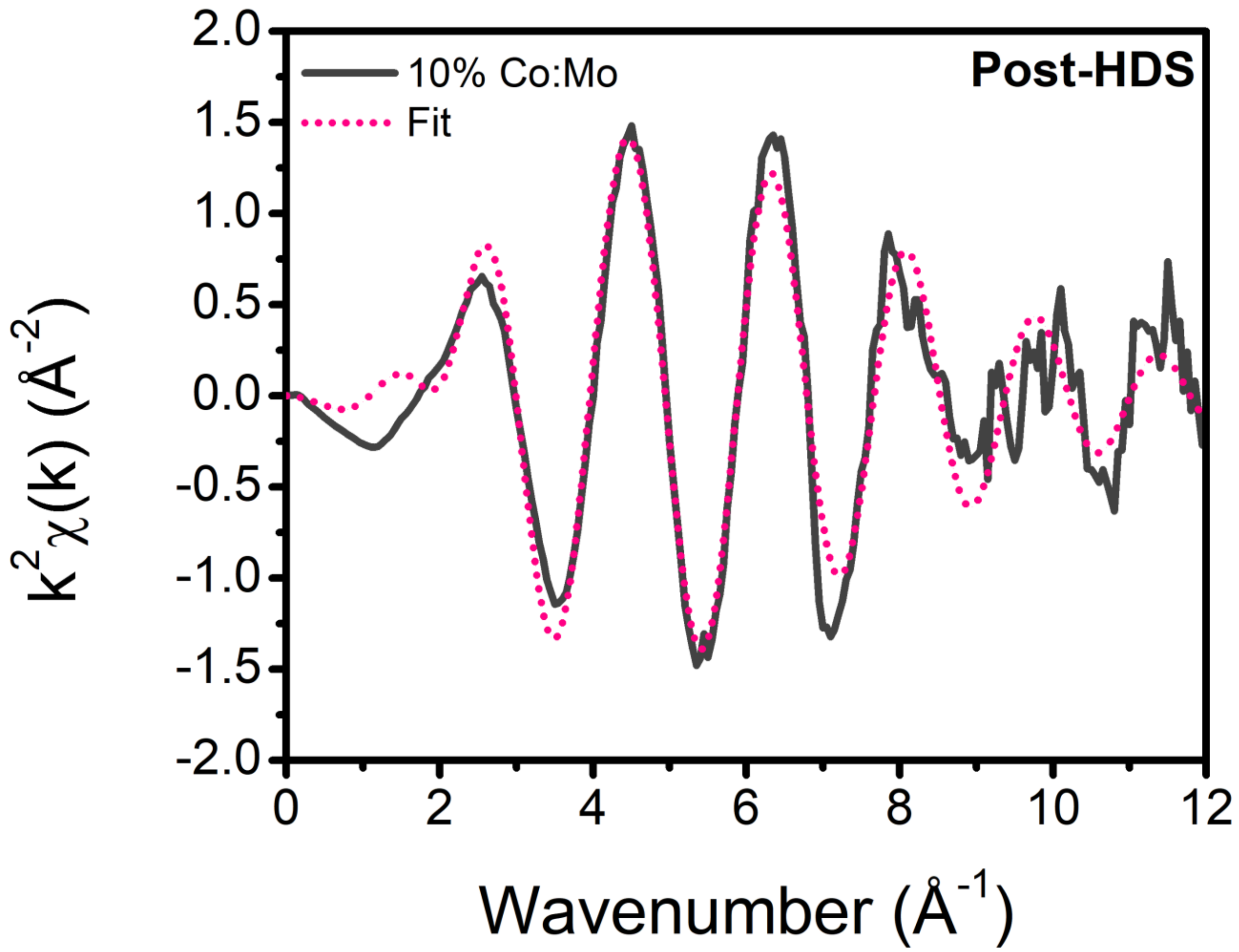

Figure S20. Artemis fitting in k-space of the Co K-edge on the 10% Co:Mo post-HDS catalyst.

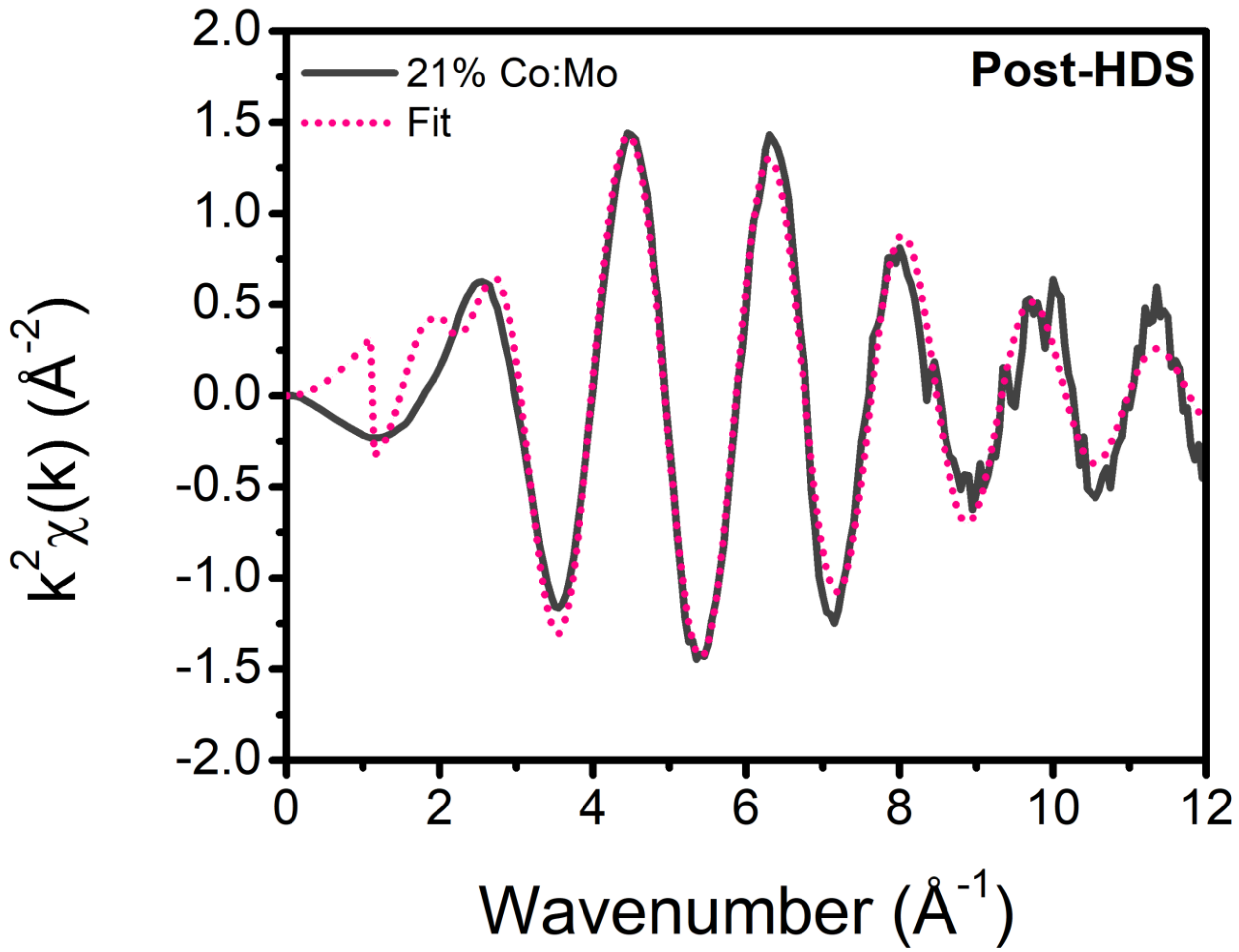

Figure S21. Artemis fitting in k-space of the Co K-edge on the 21% Co:Mo post-HDS catalyst.

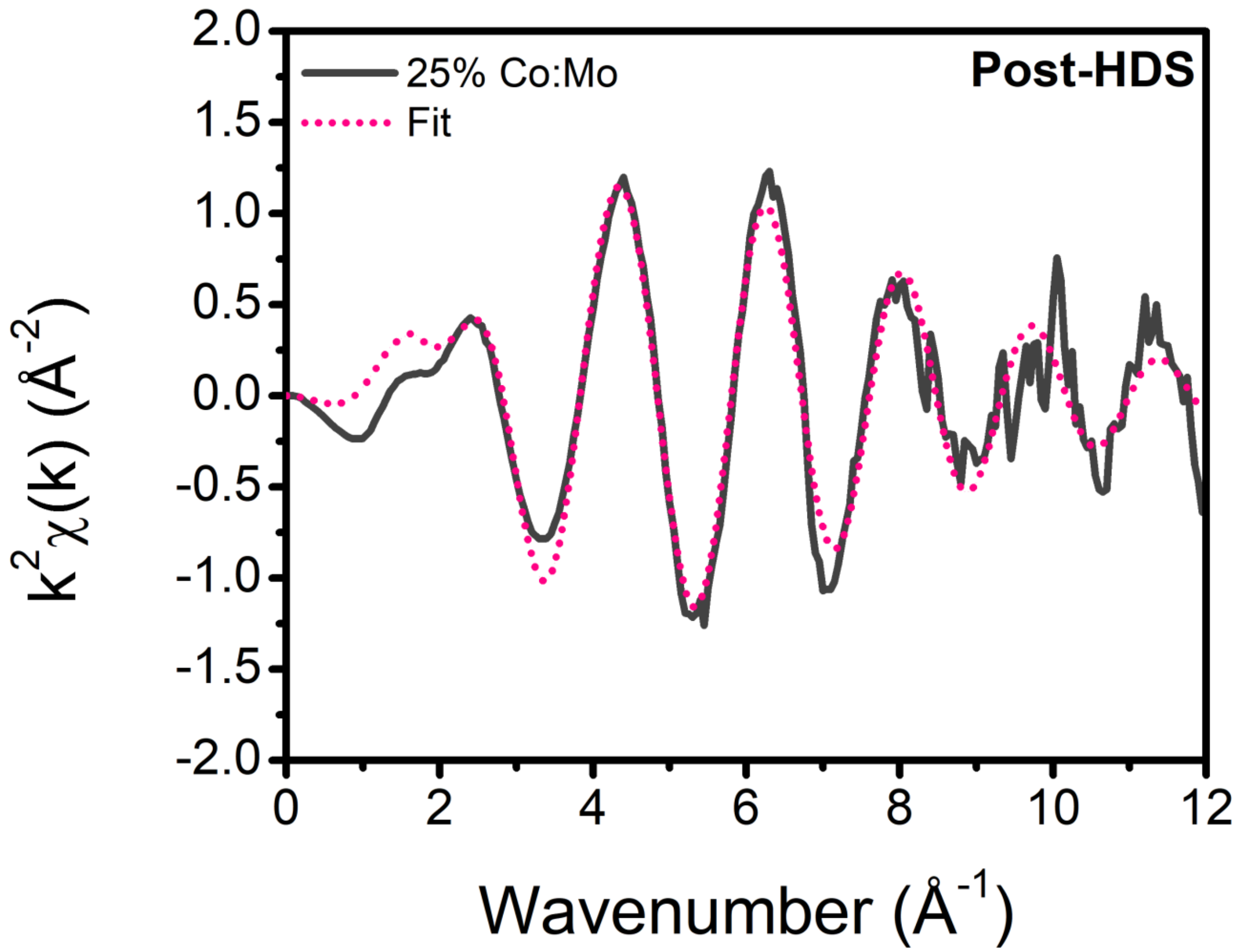

Figure S22. Artemis fitting in k-space of the Co K-edge on the 25% Co:Mo post-HDS catalyst.

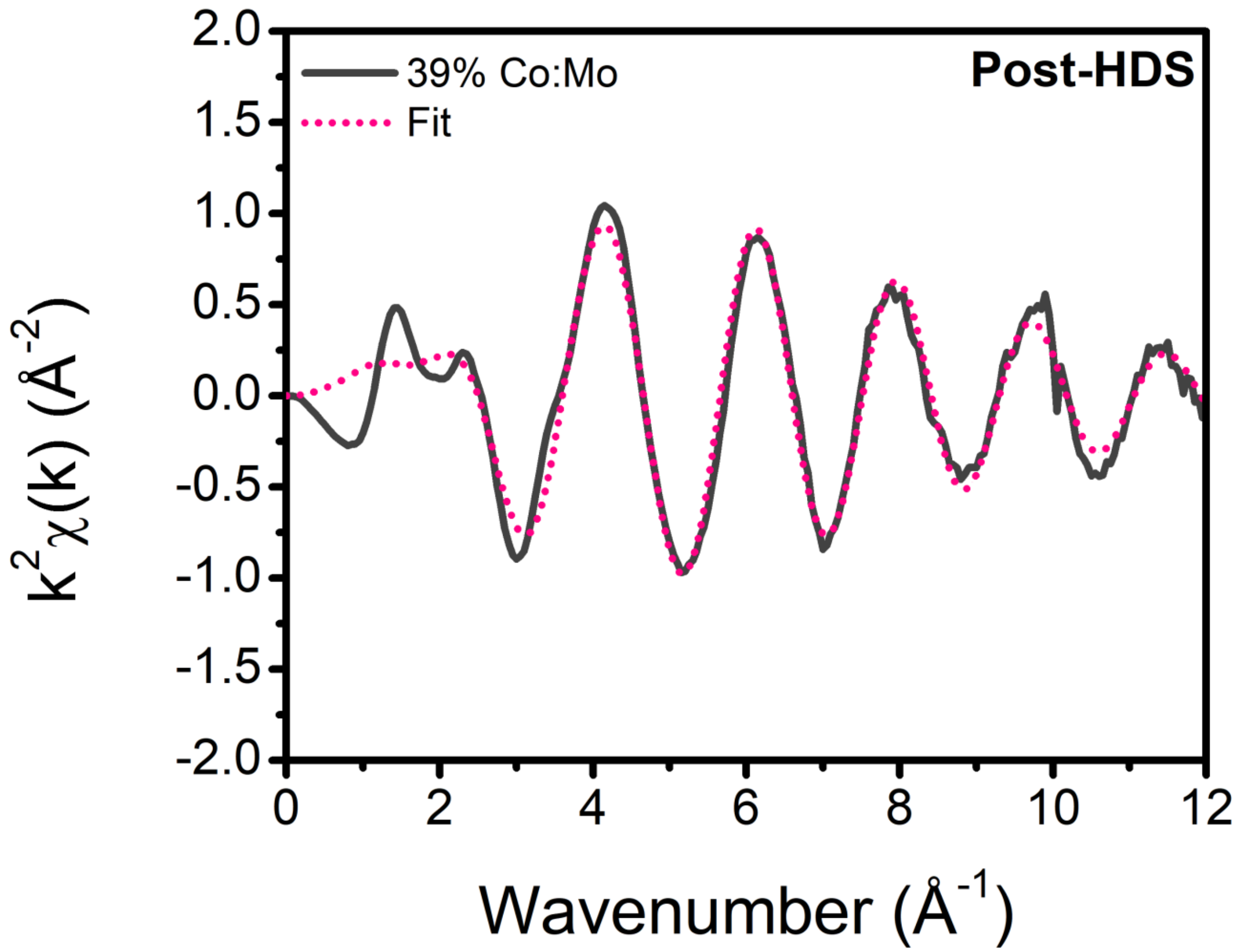

Figure 23. Artemis fitting in k-space of the Co K-edge on the 39% Co:Mo post-HDS catalyst.

Table S1. Results and best fit parameters of EXAFS modeling at the Mo K-edge for the different Co:Mo catalysts post-HDS.

Co:Mo	Path	Coord.[a]	R (Å)	σ^2 (Å^2)	ΔE_0 (eV)	R-Factor
0% (Fresh)	Mo-S	4.6(3)	2.389(6)	0.009(1)	-1.5(5)	1.89%
	Mo-Mo	1.5(6)	2.73(3)	0.012(6)		
0%	Mo-S	6.6(5)	2.405(6)	0.0033(7)	1.4(8)	0.91%
	Mo-Mo	3.4(4)	3.161(8)	0.0033(5)		
10%	Mo-S	6.0(2)	2.408(5)	0.0031(7)	2.3(7)	0.65%
	Mo-Mo	3.0(4)	3.166(8)	0.004(2)		
21%	Mo-S	6.5(2)	2.409(4)	0.0033(6)	1.7(6)	0.54%
	Mo-Mo	3.3(4)	3.167(6)	0.004(2)		
25%	Mo-S	5.9(4)	2.407(6)	0.0035(8)	2.4(8)	0.88%
	Mo-Mo	2.5(4)	3.164(9)	0.0033(6)		
39%	Mo-S	5.6(4)	2.409(6)	0.0031(7)	1.5(8)	0.83%
	Mo-Mo	2.6(4)	3.164(8)	0.0034(6)		

a- Coordination Number

Table S2. Results and best fit parameters of EXAFS modeling at the Co K-edge for the different Co:Mo fresh catalysts.

Co:Mo	Path	Coord.	R (Å)	σ^2 (Å^2)	ΔE_0 (eV)	R-Factor
10%	Co-S	4.5(6)	2.253(6)	0.004(1)	-3.6(7)	2.04%
	Co-O	0.6(5)	1.94(4)	0.000(8)		
21%	Co-S	2.6(2)	2.258(5)	0.004(1)	-1.3(4)	0.81%
	Co-O	2.5(3)	2.02(1)	0.005(2)		
25%	Co-S	1.8(2)	2.27(1)	0.004(1)	-2(3)	0.39%
	Co-O	4.1(3)	2.045(4)	0.006(1)		
39%	Co-S	1.1(2)	2.28(1)	0.004(1)	-0(2)	0.42%
	Co-O	4.2(3)	2.056(4)	0.005(1)		

Table S3. Results and best fit parameters of EXAFS modeling at the Mo K-edge for the different Co:Mo catalysts post-HDS.

Co:Mo	Path	Coord.	R (Å)	σ^2 (Å^2)	ΔE_0 (eV)	R-Factor
10%	Co-S	4.5(7)	2.221(7)	0.005(2)	0(2)	2.47%
	Co-O	0.6(6)	1.95(4)	0.000(9)		
21%	Co-S	3.9(3)	2.247(3)	0.0045(7)	5(1)	0.32%
	Co-O	1.9(2)	2.021(7)	0.003(2)		
25%	Co-S	3.8(5)	2.224(6)	0.006(1)	0(2)	1.50%
	Co-O	2.6(8)	2.04(2)	0.008(5)		
39%	Co-S	4.0(5)	2.206(5)	0.009(2)	-6(2)	0.93%
	Co-O	2.9(4)	2.047(6)	0.002(1)		

Wavelet Transforms

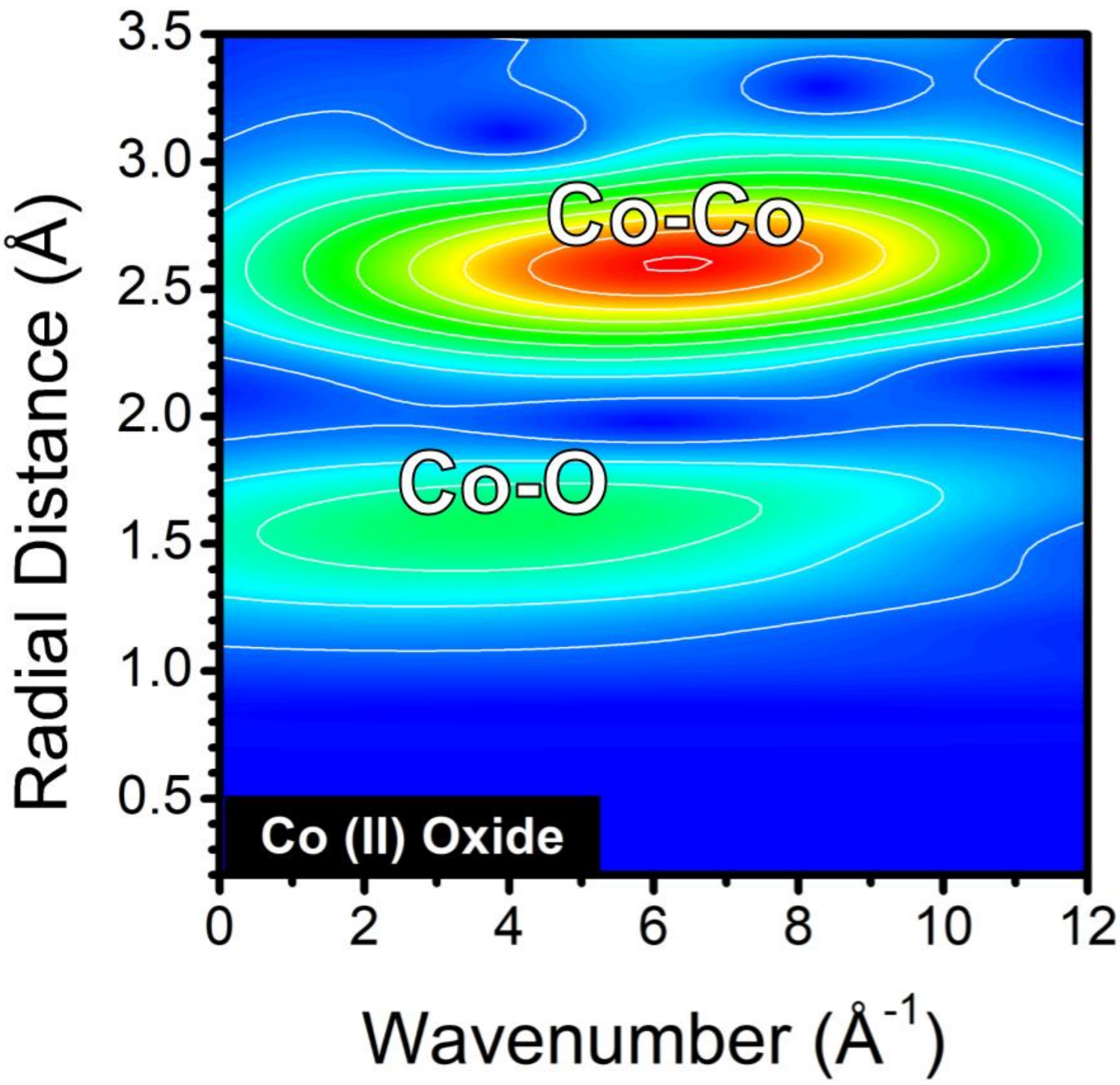

Figure S24. Co K-edge wavelet transform (WT) of cobalt (II) oxide reference material, with Co-O and Co-Co paths.

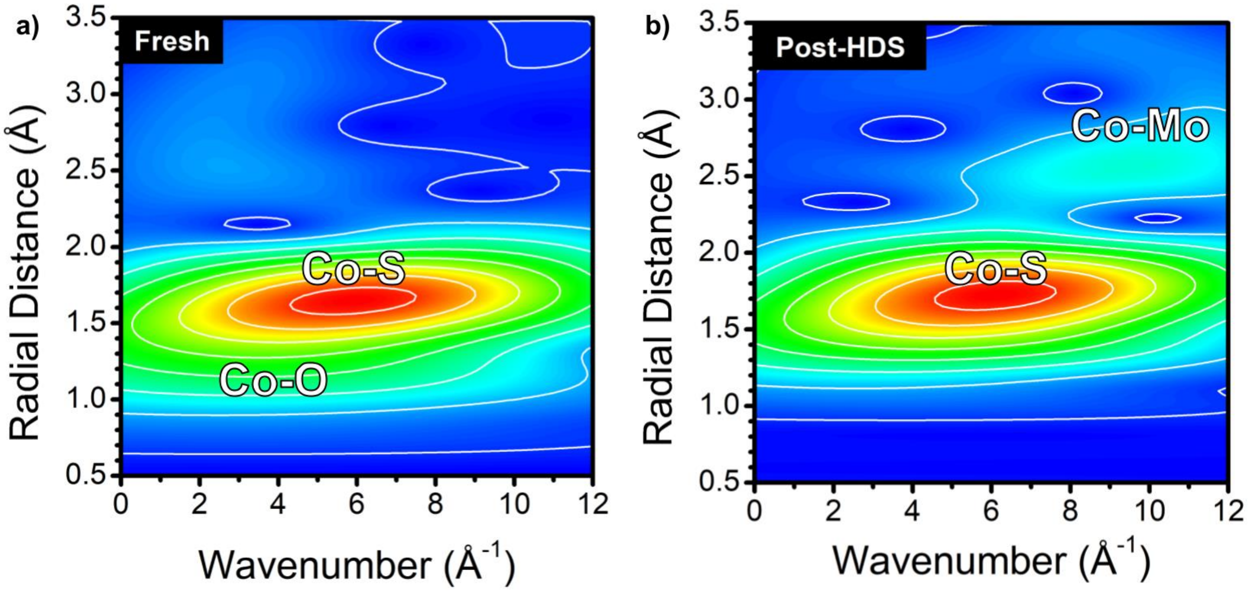

Figure S25. Co K-edge WT of the 25% Co:Mo catalyst fresh (a) and post-HDS (b). The stronger influence of oxidation (Co-O) is more obvious in this compared with 21% Co:Mo (Figure 6c in the main text), as seen by the stronger features at lower radial distance.

X-ray Photoelectron Spectroscopy

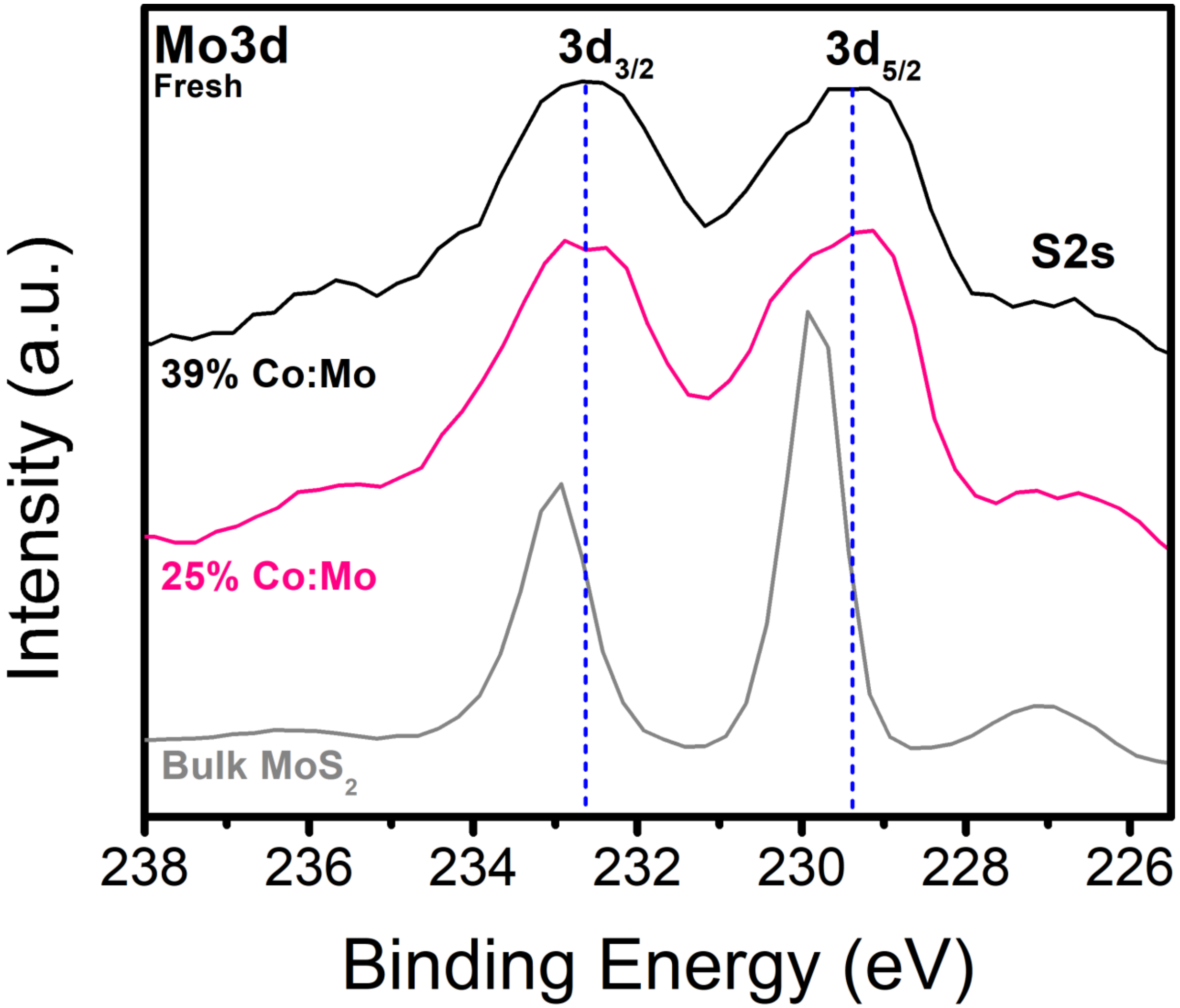

Figure S26. Mo 3d orbital XPS of fresh catalyst (ligands removed) compared with bulk MoS_2 reference. Spectra are normalized for comparison.

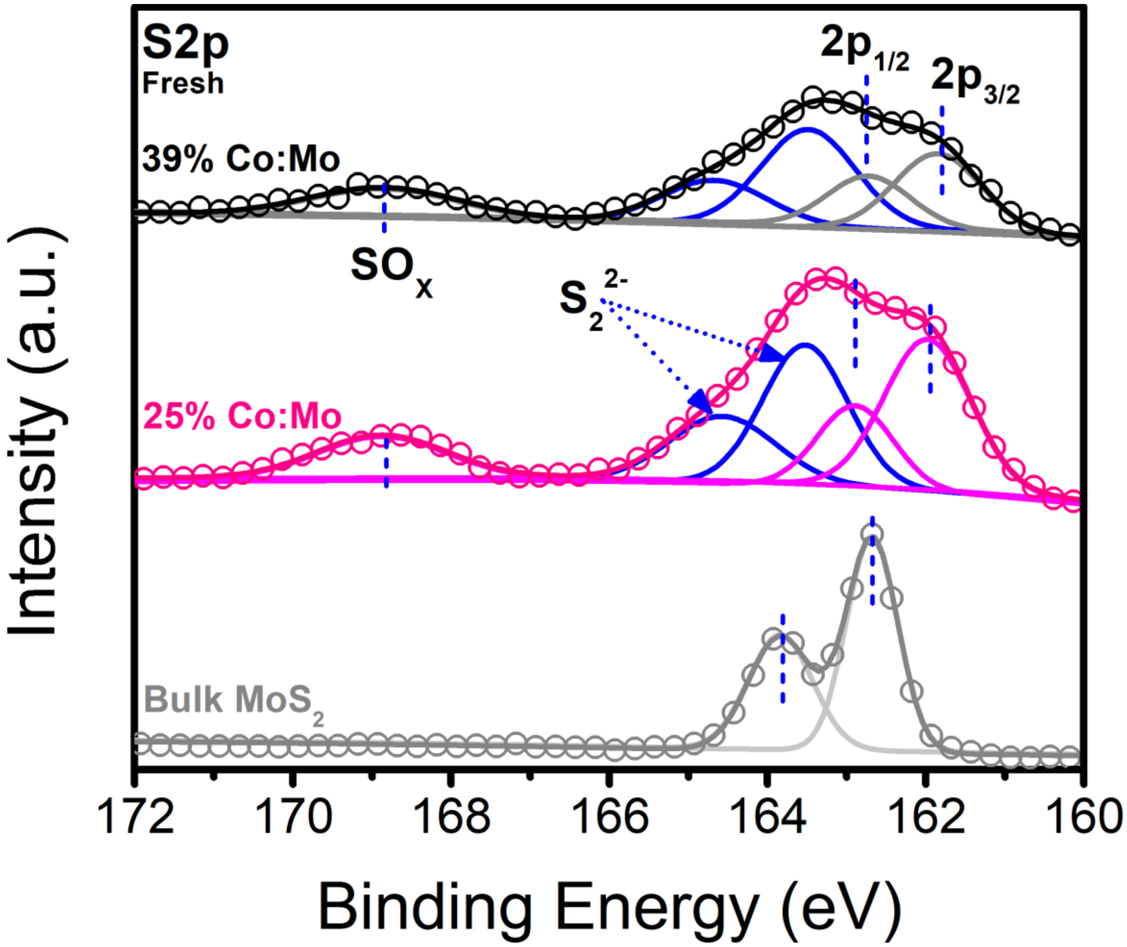

Figure S27. S 2p orbital XPS of fresh catalyst (ligands removed) compared with bulk MoS_2 reference. Spectra are normalized for comparison.

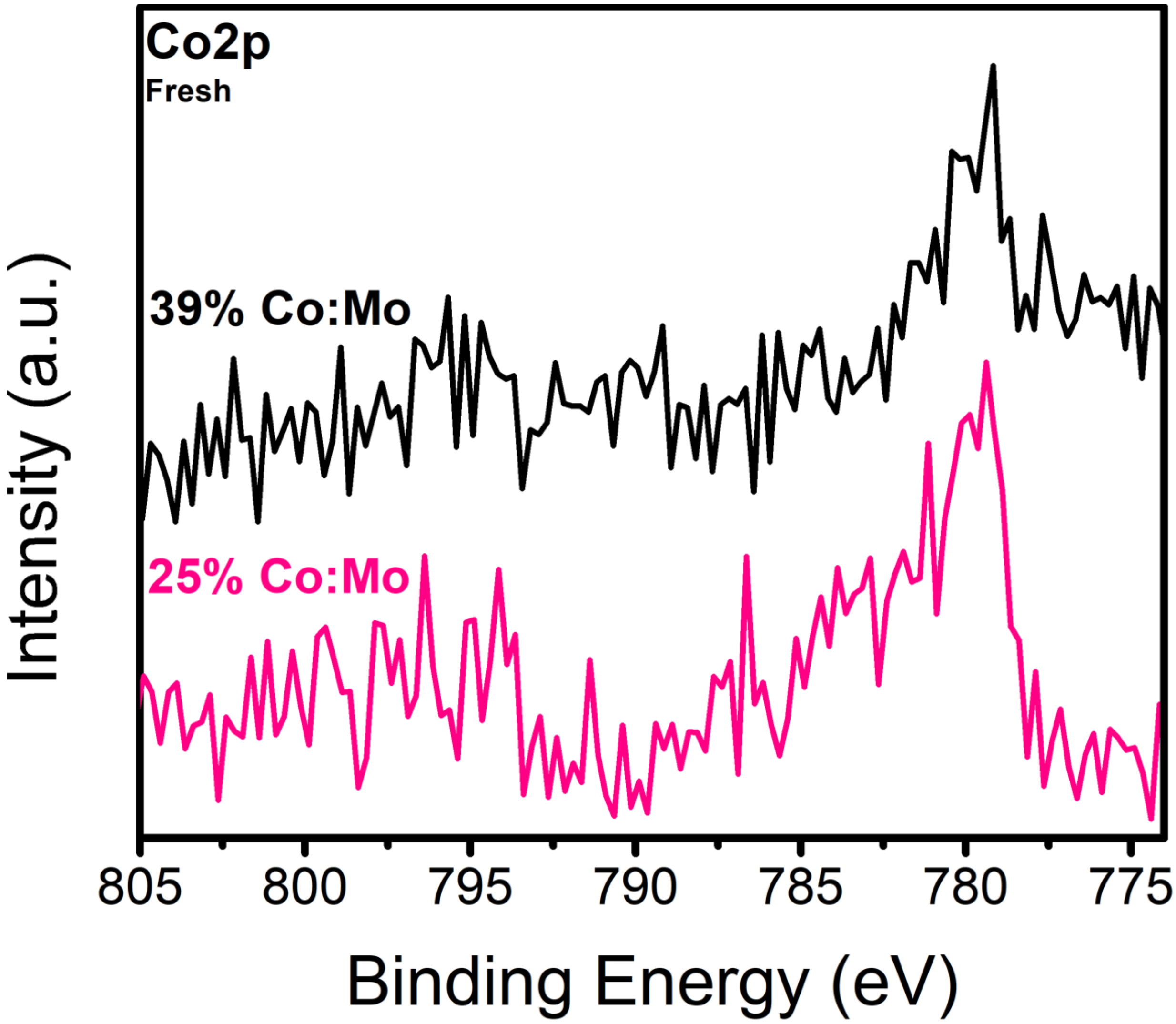

Figure S28. Co 2p orbital XPS of fresh catalyst (ligands removed).

Fourier Transform Infrared Spectroscopy

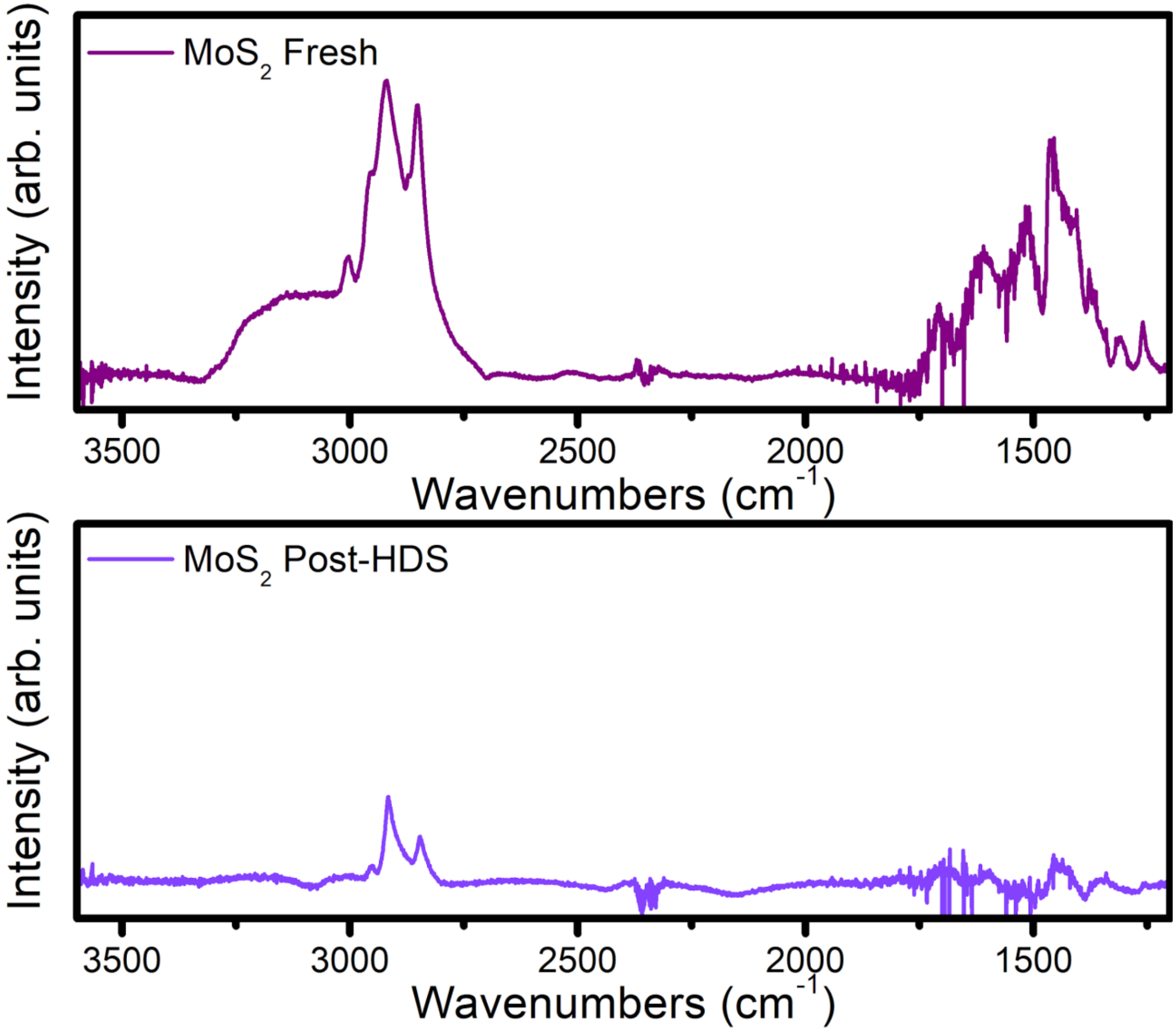

Figure S29. FT-IR of 0% Co:Mo MoS_2 before and after 3 hrs use in HDS. The loss of peak intensity suggests the removal of ligands from the surface of MoS_2 during the reaction. The small peaks between 2300 and 2400 cm^{-1} are artifacts from ambient CO_2 present in the testing chamber subtracted from the background.

HRTEM of Single Nanosheets

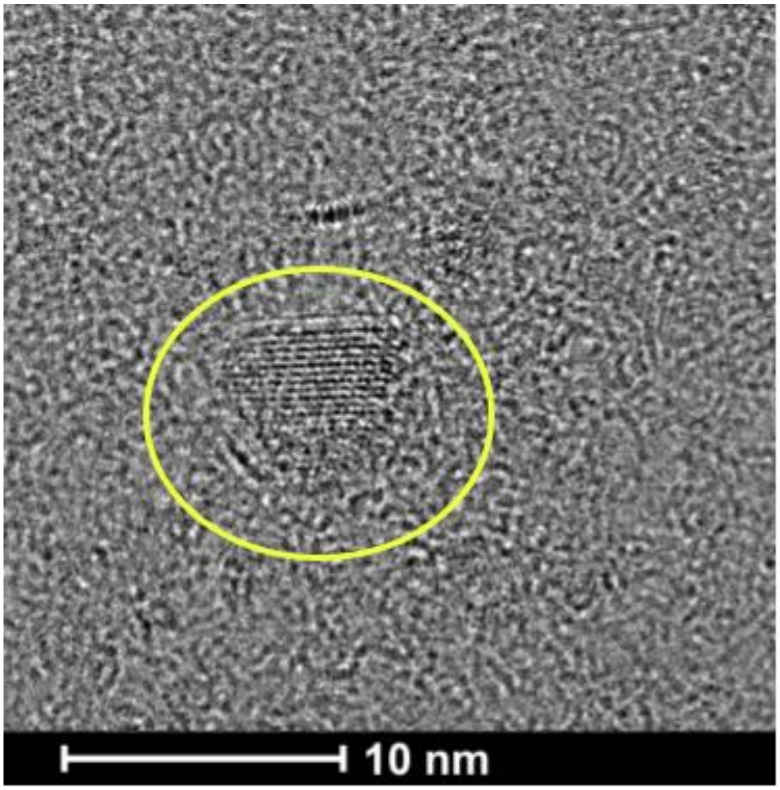

Figure S30. HRTEM of a fresh single 35% Co:Mo MoS_2 nanosheet highlighted within the yellow demonstrating the truncated triangular morphology that we assume for the calculation of edge saturation.

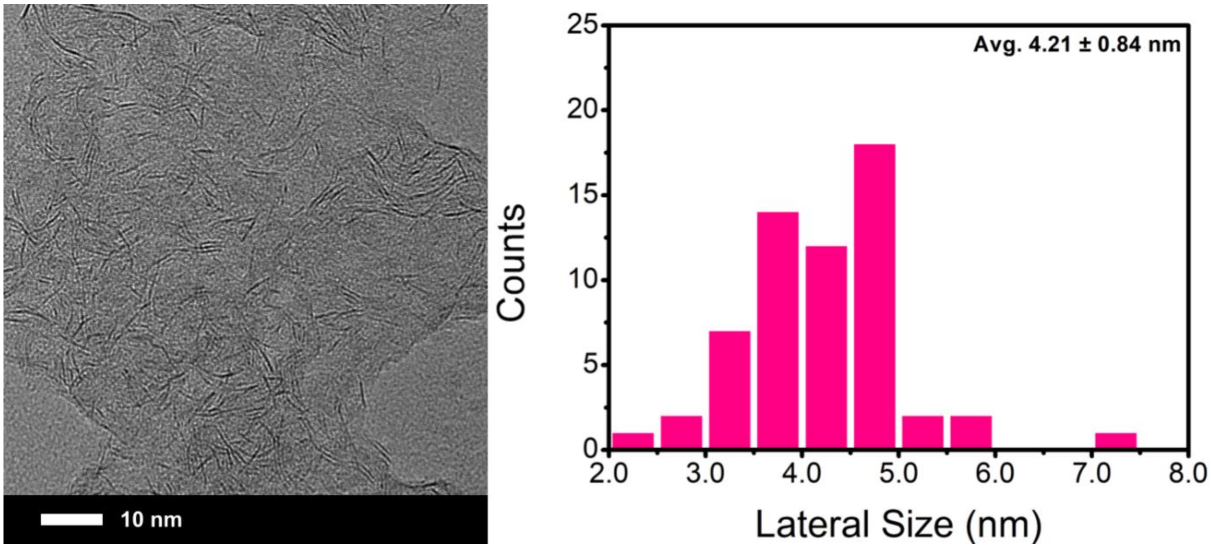

Figure S31. HRTEM of post-HDS 21% Co:Mo MoS_2 nanosheets. The dark lines represent nanosheets turned on their side, which are measured to obtain the average sheet width. Population size = 60 nanosheets.

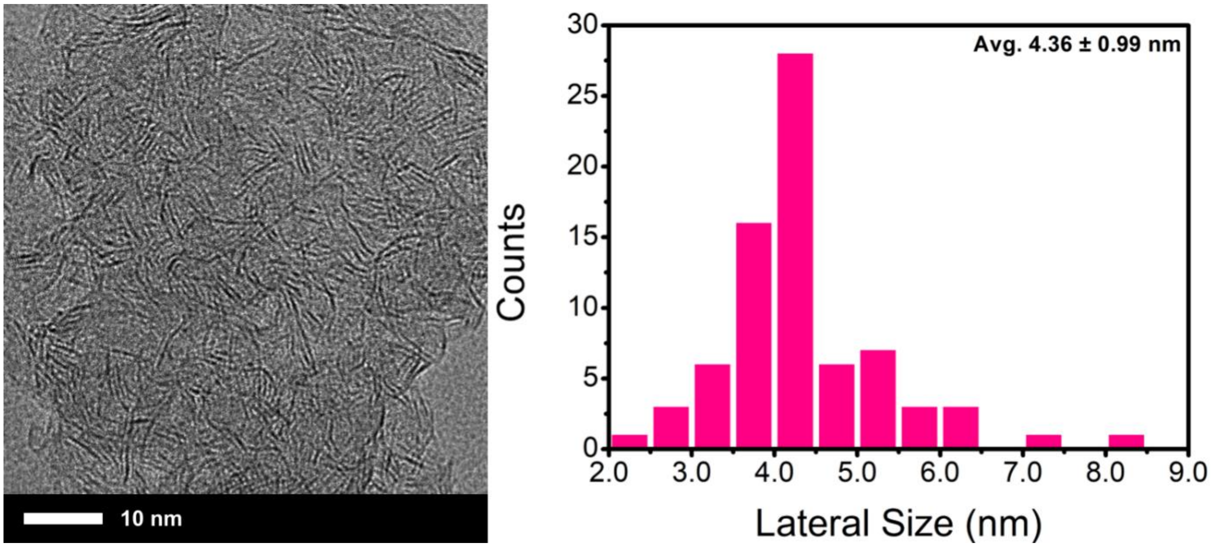

Figure S32. HRTEM of post-HDS 21% Co:Mo MoS_2 nanosheets. Population size = 75 nanosheets.

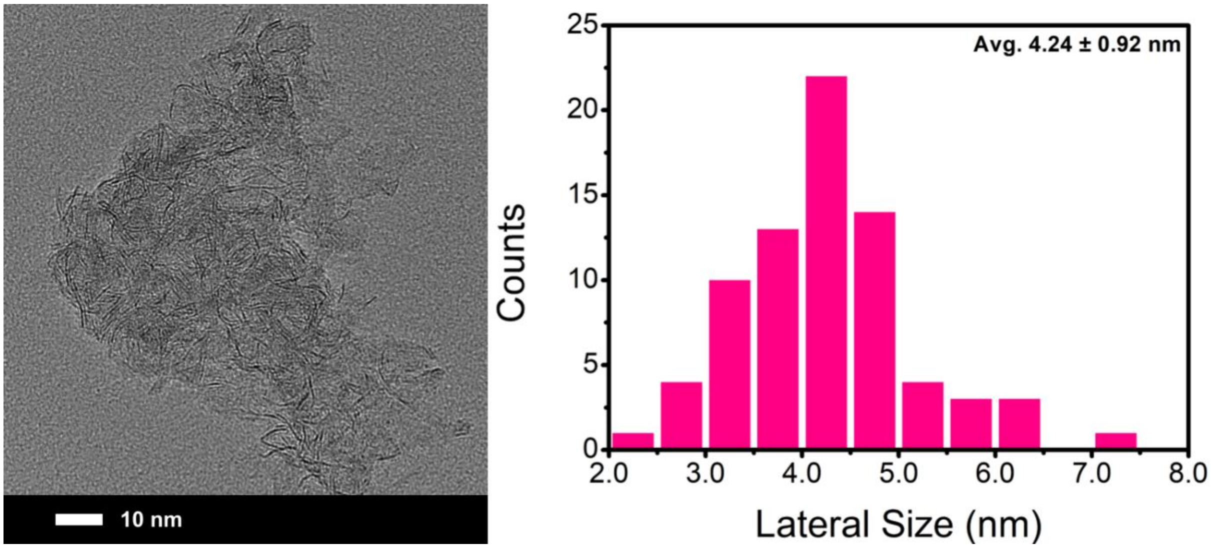

Figure S33. HRTEM of post-HDS 21% Co:Mo MoS_2 nanosheets. Population size = 75 nanosheets.

Calculation of Edge Site Saturation

Three geometries were considered for calculating the saturation point of Co doping on the edges. Each geometry was assumed to be 13 unit cells wide (unit cell = 3.19Å x 3.19Å in *a* and *b* dimensions). This equates to ~4.15nm wide nanosheets, close to the 4.25nm average diameter observed in HRTEM (see Figure 2b in main text).

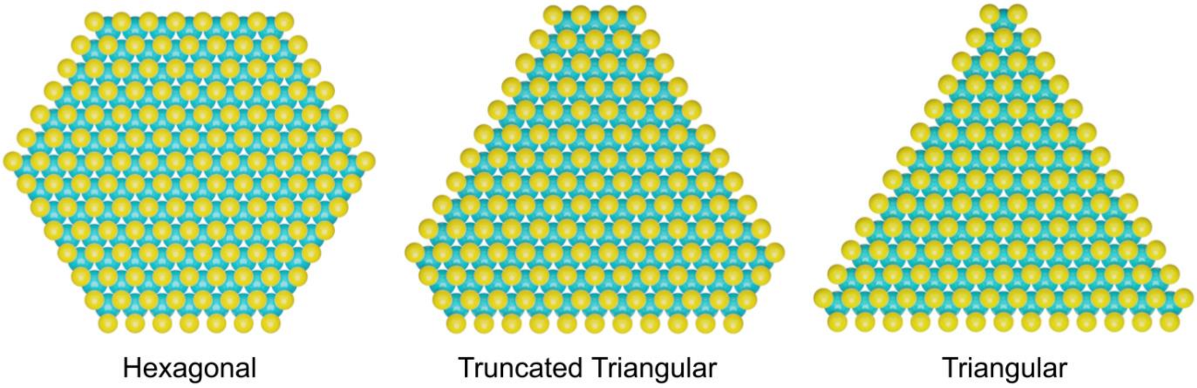

Figure S34. MoS_2 geometries considered for edge saturation calculations.

Hexagonal

A regular hexagonal morphology is assumed with 13 unit cells at its widest, with each side measuring 7 unit cells long. The sides alternate between S-edge and Mo-edge, although for the purposes of calculation we assume one Co can dope per edge unit cell regardless of the side. A hexagon represents the structure with the minimum ratio of edge sites to total Mo atoms.

Truncated Triangular

A hexagonal morphology is assumed, 13 unit cells at its widest. The three Mo-edge sides are assumed to be 11 unit cells long each, alternating with three S-edge sides that are 3 unit cells long each.

Triangular

An equilateral triangle morphology is assumed with 13 unit cells on each side. The sides are all assumed to be Mo-edge. An equilateral triangle represents the structure with the maximum ratio of edge sites to total Mo atoms.

Table S4. Calculation of atom and site counts for considered geometries of MoS_2.

Morphology	***Total Mo Atoms***	***Total Edge Sites***	***Edge Sites/ Total Mo Atoms***
Hexagonal	127	36	28.3%
Truncated Triangular	111	36	32.4%
Triangular	91	36	39.6%

Density Functional Theory

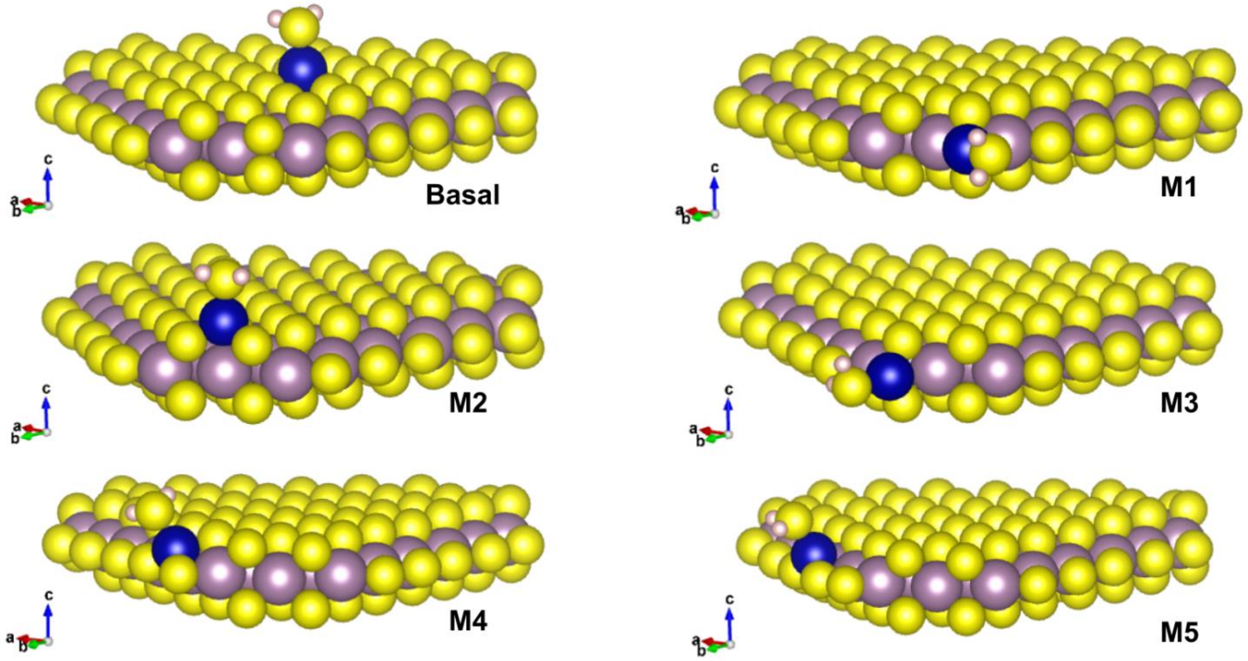

Figure S35. Models of H_2S adsorption on Co-MoS_2 showing the various calculated structures from DFT and depicted in the VESTA software.[8] Co, Mo, S, and H atoms are depicted as blue, mauve, yellow, and white, respectively.

References

1. Nečas, D.; Klapetek, P. Gwyddion: an open-source software for SPM data analysis. *Cent. Eur. J. Phys.* **2012**, *10* (1), 181-188. DOI: 10.2478/s11534-011-0096-2.
2. Ravel, B.; Newville, M. ATHENA, ARTEMIS, HEPHAESTUS: data analysis for X-ray absorption spectroscopy using *IFEFFIT. J. Synchrotron Radiat.* **2005**, *12* (4), 537-541. DOI: 10.1107/S0909049505012719.
3. Muñoz, M.; Argoul, P.; Farges, F. o. Continuous Cauchy wavelet transform analyses of EXAFS spectra: A qualitative approach. *American Mineralogist* **2003**, 88 (4), 694-700. DOI: 10.2138/am-2003-0423.
4. Kim, M.; Park, G. H.; Seo, S.; Bui, V. Q.; Cho, Y.; Hong, Y.; Kawazoe, Y.; Lee, H. Uncovering the Role of Countercations in Ligand Exchange of WSe_2: Tuning the d-Band Center toward Improved Hydrogen Desorption. *ACS App. Mater. Interfaces* **2021**, *13* (9), 11403-11413. DOI: 10.1021/acsami.0c19865.
5. Jain, A.; Ong, S. P.; Hautier, G.; Chen, W.; Richards, W. D.; Dacek, S.; Cholia, S.; Gunter, D.; Skinner, D.; Ceder, G.; Persson, K. A. Commentary: The Materials Project: A materials genome approach to accelerating materials innovation. *APL Mater.* **2013**, *1*, 011002. DOI: 10.1063/1.4812323.
6. Kwon, I. S.; Debela, T. T.; Kwak, I. H.; Park, Y. C.; Seo, J.; Shim, J. Y.; Yoo, S. J.; Kim, J. G.; Park, J.; Kang, H. S. Ruthenium Nanoparticles on Cobalt-Doped 1T' Phase MoS_2 Nanosheets for Overall Water Splitting. *Small* **2020**, *16* (13), e2000081. DOI: 10.1002/smll.202000081.
7. Liu, Q.; Fang, Q.; Chu, W.; Wan, Y.; Li, X.; Xu, W.; Habib, M.; Tao, S.; Zhou, Y.; Liu, D.; Xiang, T.; Khalil, A.; Wu, X.; Chhowalla, M.; Ajayan, P. M.; Song, L. Electron-Doped

1T-MoS_2 via Interface Engineering for Enhanced Eletrocatalytic Hydrogen Evolution. *Chem. Mater*. **2017**, *29* (11), 4738-4744. DOI: 10.1021/acs.chemmater.7b00446.

8. Momma, K.; Izumi, F.; *VESTA 3* for three-dimensional visualization of crystal, volumetric and morphology data. *J. Appl. Cryst.* **2011**, *44*, 1272-1276. DOI: 10.1107/S0021889811038970.
